# Supplementary material for: Biocompatible aggregation-induced emission active polyphosphate-manganese nanosheets with glutamine synthetase-like activity in excitotoxic nerve cells
Source: Nat Commun. 2024 Apr 26;15:3534. doi: 10.1038/s41467-024-47947-5 (PMC11053040; doi:10.1038/s41467-024-47947-5)
Supplement: Supplementary file 1 — Supplementary Information [file 41467_2024_47947_MOESM1_ESM.pdf]

Supplementary Information

Biocompatible Aggregation-Induced Emission Active Polyphosphate-Manganese Nanosheets with Glutamine Synthetase-like Activity in Excitotoxic Nerve Cells

Jing Wang,[1] Xinyang Zhao,[1] Yucheng Tao,[2] Xiuxiu Wang,[1] Li Yan,[4] Kuang Yu,[6] Yi Hsu,[7] Yuncong Chen*[1,4], Jing Zhao,*[1,3,4] Yong Huang,*[5] Wei Wei *[1,2,3,4]

[1]State Key Laboratory of Coordination Chemistry, Chemistry and Biomedicine Innovation Center (ChemBIC), School of Chemistry and Chemical Engineering, Nanjing University, Nanjing 210093, China.

[2]School of Life Sciences, Nanjing University, Nanjing 210093, China.

[3]Shenzhen Research Institute, Nanjing University, Shenzhen, China.

[4]Nanchuang (Jiangsu) Institute of Chemistry and Health, Sino-Danish Ecolife Science Industrial Incubator; Jiangbei New Area, Nanjing, 210000, China

[5]Department of Chemistry, The Hong Kong University of Science and Technology, Clear Water Bay, Kowloon, Hong Kong SAR, China

[6]Tsinghua-Berkeley Shenzhen Institute and Institute of Materials Research (iMR), Tsinghua Shenzhen International Graduate School, Tsinghua University, Shenzhen, Guangdong, P. R. China

[7]Taipei Wego Private Senior High School, Taipei, TWN, China.

E-mail: chenyc@nju.edu.cn, Jingzhao@nju.edu.cn, yonghuang@ust.hk, weiwei@nju.edu.cn

Experimental Section

Reagents and cell lines. Polyphosphate is composed of approximately 45 phosphate groups (polyP$_{45}$) and is available commercially and we purchased it from Sigma. Natural GS was purchased from Sigma (USA). $MnCl_2$ was purchased from Sigma (USA). Hexadecyl trimethyl ammonium bromide (CTAB) was purchased from Mackli. Sodium oleate and 2,2-(ethylenedioxy)bis-(ethylamine) were purchased from Aladdin. 1-Bromo-1,2,2-triphenylethylene were obtained from Mackli. The above reagents were used as received without any further purification. SH-SY5Y was obtained from the China Type Culture Collection (catalog number: SCSP-5014, CSTR:19375.09.3101HUMSCSP5014). PC-12 cells were obtained from the China Type Culture Collection (catalog number: SCSP-517, CSTR:19375.09.3101RATSCSP517). U87 cells were obtained from ATCC (HTB-14). The cell lines were listed by the International Cell Line Authentication Committee as cross-contaminated or misidentified. SH-SY5Y cells were cultured in MEM/F12 + 10% FBS, U87 cells were cultured in MEM + 1% Non-Essential Amino Acids (NEAA) + 1mM Sodium Pyruvate (NaP) + 10% FBS and PC-12 cells were cultured in RPMI-1640+10%HS+5%FBS at 37 °C in a CO_2 incubator (95% relative humidity, 5% CO_2).

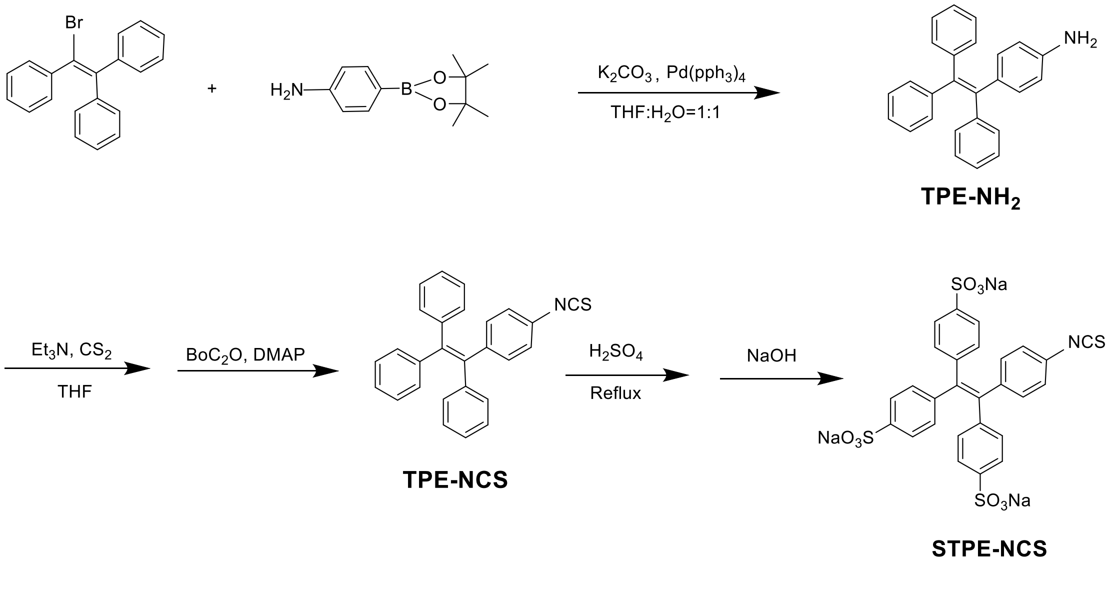

Supplementary Fig 1. Synthetic route to sodium 4,4',4''-(2-(p-tolyl)ethene-1,1,2-

triyl)tribenzenesulfonate (STPE-NCS).

Preparation of TPE-NH_2. 1-Bromo-1,2,2-triphenylethylene (3.35 g, 10.0 mmol 1.0 equiv), (4,4,5,5-Tetramethyl-1,3,2-dioxaborolan-2-yl)aniline (2.30 g, 10.5 mmol 1.05 equiv), potassium carbonate (4.15 g, 30 mmol 3.0 equiv), and tetrakis(triphenylphosphine)palladium (347 mg, 3 mol%) were charged in a 350 mL pressure flask. THF (50 mL) and H_2O (25 mL) were added under a nitrogen atmosphere and the mixture was refluxed overnight. After cooling to room temperature, the mixture was extracted with DCM three times. The combined organic phase was concentrated and 4-(1,2,2-Triphenylethenyl) benzylamine (white solid, 3.0 g, 86%) was afforded by silica gel chromatography (petroleum ether: dichloromethane = 1: 1). ^{1}H NMR (400 MHz, DMSO-*d*$_6$) δ 7.14 (dd, *J* = 8.2, 6.4 Hz, 1H), 7.08 (td, *J* = 6.5, 3.8 Hz, 3H), 7.06 – 6.98 (m, 3H), 6.98 – 6.88 (m, 2H), 6.62 – 6.56 (m, 1H), 6.32 – 6.26 (m, 1H), 5.05 (s, 1H). ^{13}C NMR (101 MHz, DMSO-*d*$_6$) δ 147.76, 144.67, 144.45, 144.42, 141.67, 138.35, 132.10, 131.36, 131.29, 131.20, 130.79, 128.24, 128.13, 128.03, 126.72, 126.47, 126.45, 113.60, 40.65, 40.44, 40.23, 40.02, 39.81, 39.60, 39.39. HRMS (ESI, CH_3CN, m/z, relative intensity): 347.1663 (calcd for [$C_{26}H_{21}N$] +: 347.1674).

Preparation of TPE-NCS. We synthesized TPE-NCS according to the existing route with slight changes[1]. 4-(1,2,2-Triphenylethenyl) benzylamine (1.74 g, 5 mmol 1.0 equiv) in 15 mL dry ethanol was added triethylamine (675 μL, 5 mmol 1.0 equiv) and carbon disulfide (3 mL, 50 mmol 10.0 equiv) at room temperature for 30 min. The mixture was cooled in an ice bath and then di-tert-butyl dicarbonate (Boc_2O) (1.08 g, 4.95 mmol 0.99 equiv) dissolved in dry ethanol (5 mL) was added, followed by the immediate addition of DMAP (6 mg, 1 mol %) in dry ethanol (1 mL). The mixture was kept in an ice bath for 10 min and allowed to reach room temperature for another 30 min. The solvent was concentrated and TPE-NCS (yellow oil, 1.3 g, 67%) was afforded by silica gel chromatography (petroleum ether: ethyl acetate = 20: 1). ^{1}H NMR (400 MHz, DMSO-*d*$_6$) δ 7.21 – 7.07 (m, 11H), 7.04 – 6.89 (m, 8H). ^{13}C NMR (101 MHz, DMSO-*d*$_6$) δ 143.44, 143.26, 143.21, 143.05, 142.10, 139.74, 134.01, 133.82, 133.62,

132.55, 131.13, 131.11, 131.04, 129.44, 129.39, 129.26, 129.19, 128.47, 128.43, 128.29, 127.34, 127.26, 127.20, 125.90, 40.65, 40.44, 40.23, 40.02, 39.81, 39.60, 39.39. HRMS (ESI, CH_3CN, m/z, relative intensity): 389.1235 (calcd for $[C_{27}H_{19}NS]^+$: 389.1238).

Synthesis of water-soluble TPE-NCS (STPE-NCS). We synthesized STPE-NCS according to the existing route with slight changes[2]. TPE-NCS (500 mg, 1.28 mmol) was added to conc. H_2SO_4 (10 mL) in a 75 mL pressure flask and the mixture was stirred and heated at 120 °C for 4 h. The mixture was then slowly added to ethyl acetate at 0 °C and placed in -20 °C refrigerator for 4 h. The precipitate was filtered and dissolved in water, which was neutralized with aqueous NaOH. The solution was then added to acetone, and the precipitate was filtered and washed with acetone three times to afford STPE (light yellow solid, 310 mg, 35%). ^1H NMR (400 MHz, D_2O) δ 7.55 – 7.43 (m, 6H), 7.19 (dq, J = 8.5, 2.0 Hz, 4H), 7.16 – 7.10 (m, 2H), 6.86 – 6.78 (m, 2H), 6.57 – 6.49 (m, 2H). ^{13}C NMR (101 MHz, D_2O) δ 146.64, 146.46, 146.19, 145.65, 142.36, 140.67, 140.47, 140.40, 138.44, 133.21, 132.43, 131.71, 131.69, 131.60, 125.06, 125.02, 124.95, 115.48. HRMS (ESI, CH_3OH, m/z, relative intensity): 622.9462 (calcd for $[C_{27}H_{16}NO_9S_4 - 3H]^-$: 622.9468).

Synthesis of amine-labeled polyP-NH_2. We synthesized polyP-NH_2 according to Wender's route with slight changes[3]. PolyP 45 was dissolved in 1 M MES buffer to a final concentration of 50 mM. 2,2′-(Ethylenedioxy)bis(ethylamine) (20 equiv.) was added to the polyP solutions followed by 1-ethyl-3-(3-dimethylaminopropyl) carbodiimide hydrochloride (EDAC) (20 equiv.) and the pH was adjusted to 6.5 with 1 M HCl. The mixture was heated to 60 °C for 2 h, after which time the pH of the reaction was increased to 10 with 1 M NaOH, and the crude product was purified by precipitation into 45 mL ethanol (3x). The precipitate was collected by centrifugation (6 min at 1100 x g) and lyophilized to dryness. ^1H NMR (400 MHz, Deuterium Oxide) δ 3.77 – 3.54 (m, 16H), 3.20 – 3.10 (m, 4H), 3.09 – 3.00 (m, 4H).^{13}C NMR (101 MHz, D_2O) δ 69.57, 66.50, 39.00. ^{31}P NMR (162 MHz, D_2O) δ 2.67, -0.69, -0.78, -5.78, -5.90,

-20.88, -21.49, -21.51, -21.51, -21.72, -21.98, -22.41, -22.99, -23.73, -23.75, -23.95.

Preparation of STPE-labeled polyP (STPE-polyP). PolyP-NH_2 was dissolved in 0.1 M carbonate buffer (pH 9.0) to a final concentration of 230 mM. STPE (4 equiv) was added and the reaction was stirred for 20 hours at room temperature. The crude product was purified by precipitation into 45 mL of ethanol (3x), collected by centrifugation (15 min at 1100 x g), dissolved in alkaline water (pH ~ 8.0), and then further purified by preparative size exclusion chromatography (Sephadex 25). The samples were lyophilized for 2 days. ^{1}H NMR (400 MHz, D_2O) δ 7.60 – 7.43 (m, 6H), 7.28 – 7.12 (m, 6H), 6.85 (d, J = 8.5 Hz, 2H), 6.55 (d, J = 8.5 Hz, 2H), 3.77 – 3.51 (m, 35H), 3.19 – 3.00 (m, 16H).^{13}C NMR (101 MHz, D_2O) δ 146.55, 146.38, 146.13, 142.25, 140.67, 140.49, 140.42, 138.62, 133.91, 132.43, 131.66, 131.58, 125.05, 125.02, 124.96, 116.00, 69.57, 66.50, 39.00. ^{31}P NMR (162 MHz, D_2O) δ -0.71, -5.58, -21.50, -21.54, -21.71, -21.94, -22.42, -23.02, -23.75, -23.98.

Preparation of STPE-PMNSs. The STPE-PMNSs were constructed with a hierarchical assembly strategy with the assistance of CTAB and sodium oleate at room temperature. A solution of STPE-polyP (0.032 mmol, 220 mg), and CTAB (0.75 mmol, 273.33 mg) in ddH_2O (15 mL, pH 7.4) was prepared. Then, the system was ultrasonically dispersed for 30 min. A solution of $MnCl_2$ (0.735 mmol, 91.9 mg) and sodium oleate (0.8 mmol, 243.2 mg) in ddH_2O (15 mL, pH 6.5) was stirred for 30 min at room temperature. Briefly, the STPE-polyP/CATB solution was added to aqueous Mn^{2+}-oleate aqueous. The obtained mixed solution was stirred for 2 h, and the resulting brown product was obtained by centrifugation and washed with ethanol and ddH_2O. The samples were lyophilized for 2 days.

Characterization of nanomaterials. TEM and AFM images of STPE-PMNSs were obtained from Hitachi HT7700 and Bruker MultiMode 8, respectively. STEM-HAADF images were obtained from Themis Z (Thermo Scientific). Zeta potential and size of STPE-PMNSs were measured on Nano-ZS instrument (Malvern Instruments Limited)

and Brookhaven BI-200SM, respectively. FT-IR was obtained from Nicolet 7000-c. The fluorescence emission spectra were measured on a fluorescence spectrophotometer (Shimadzu, RF-6000). XRD patterns were recorded on an X-ray diffractometer (Bruker D8 Advance) with Cu Kα radiation (λ = 1.54060 Å) and XPS was measured on Thermo ESCALAB 250Xi spectroscope. HRTEM and EDXS were performed on a JEOL JEM-F200. ^{1}H NMR, ^{13}C NMR, and ^{31}P NMR spectra were obtained on Bruker nuclear resonance (400 MHz) spectrometer. HRMS data was collected on Thermo Scientific Xcalibur.

Fluorescence emission spectrum test. For STPE-polyP, 250 mg of STPE-polyP was dissolved in 5 mL of distilled water and set aside. Prepare 11 Eppendorf tubes and sequentially add 500, 450, 400, 350, 300, 250, 200, 150, 100, 50, and 0 μL of absolute ethanol. Then, 50, 100, 150, 200, 250, 300, 350, 400, 450, 500, and 550 μL of STPE-polyP aqueous solution was added. After fully shaking the mixtures, the fluorescence emission spectrum (Ex = 330 nm) was measured. All experiments were independently carried out with three replicates.

For STPE-PMNSs, STPE-PMNSs solutions with concentrations of 100, 90, 80, 70, 60, 50, 40, 30, 20, and 10 μg mL^{-1} were configured, and the fluorescence emission spectra (Ex = 330 nm) were tested after mixing. All experiments were independently carried out with three replicates.

GS-like activity assessment. To assess the activity of the natural GS, we initially prepared a reaction stock solution containing 100 mM imidazole-HCl, 50 mM Glu, 25 mM 2-mercaptoethanol, 20 mM ATP, 20 mM $MgCl_2$, and 125 mM NH_4Cl. Subsequently, a quenching agent containing 0.37 M $FeCl_3$, 0.67 M HCl, and 0.2 M trichloroacetic acid was also prepared. The enzymatic reaction was initiated by adding 50 mg of natural GS to 500 mL of the stock solution. The reaction mixture was then incubated at 37 °C for 15 min, followed by the addition of 750 mL quenching agents. The absorbance of the reaction mixture was measured at 540 nm. The GS-like activity of STPE-PMNSs was assessed in a HEPES buffer solution (100 mM, pH 7.3). A

reaction mixture was prepared containing 20 mM Glu, 20 mM ATP, and 20 mM NH_4^+ ions in the HEPES buffer. The enzymatic reaction was initiated by adding 50 mg of STPE-PMNSs to 500 mL of the reaction mixture. The reaction mixture was then incubated at 37 °C for 15 min, followed by the addition of 750 mL quenching agents. The absorbance of the reaction mixture was measured at 540 nm. Evaluation of Gln content in the reaction system by the standard curve method. All experiments were independently carried out with three replicates.

Dose-response assessment. The dose-response of Glu to Gln converted by STPE-PMNSs was determined using a colorimetric GS assay kit (Sangon Biotechnology, D799578) according to the manufacturer's instructions. In brief, aliquots of 320 μL reactants and 140 μL substrates were mixed with STPE-PMNSs (0-400 μg mL^{-1}). 150 μL was extracted in 96-well plates and incubated for 30 min at 37 °C, and added 50 μL 0.37 M $FeCl_3$, 0.67 M HCl, 0.2 M trichloroacetic acid. The absorbance of the colorimetric substrates was recorded by a microplate reader at 540 nm. Blank controls were also included at each concentration of STPE-PMNSs. The relative enzymatic activities of STPE-PMNSs were calculated as follows:

$$\text{Relative enzymatic activity (\%)} = \frac{OD_x - OD_{BL}}{OD_m - OD_{BL}} \times 100\% \qquad (1)$$

where OD_x is the absorbance of the tested sample; OD_m is the highest absorbance of STPE-PMNS under the optimal conditions; and OD_{BL} is the absorbance of blank controls. All experiments were independently carried out with three replicates.

SOD-like/GPx-like/CAT-like/LDH-like/COX-like activity assessment. The STPE-PMNSs (100 μg mL^{-1}) were in the recommended buffers of each assay kit including SOD (Beyotime Biotechnology, S0101S), GPx (Beyotime Biotechnology, S0058), CAT (Beyotime Biotechnology, S0051), LDH (Beyotime Biotechnology, C0016), and COX (Beijing Solarbio Science & Technology Co., Ltd, BC0945) assay kits. Experiments were according to the corresponding manufacturers' protocol. All experiments were independently carried out with three replicates.

Ability to reuse STPE-PMNSs. At the initial reaction cycle, 100 μg mL^{-1} STPE-PMNSs or GS was added into a 1 mL aqueous solution. After 12 h of reaction at 4 °C, the STPE-PMNSs were separated by an ultrafiltration membrane (MWCO at 3000 Da, UFC500396, Millipore) at 4000 g and 4 °C for 20 min. The pellets of STPE-PMNSs or GS above the membrane were resuspended in an equal volume of solutions for the second reaction cycle. The supernatants collected in each reaction cycle were added 500 μl of 0.37 M $FeCl_3$, 0.67 M HCl, 0.2 M trichloroacetic acid, and then added to 96-well plates (200 μL/well) to detect the absorbance at 540 nm. The suspensions containing 100 μg mL^{-1} STPE-PMNSs or GS were subjected to the same procedures and included as blanks. Six reaction cycles were performed. The percentages of remaining catalytic activities of STPE-PMNSs or GS in each reaction cycle were calculated by the following formula:

$$\text{Catalytic activity (\%)} = \frac{OD_{Rn} - OD_{BL}}{OD_{R1} - OD_{BL}} \times 100\% \quad (2)$$

where OD_{Rn} and OD_{R1} represent the absorbance of substrates in STPE or GS mediated reactions in the nth and first cycles, respectively. OD_{BL} is the absorbance of blank solutions at 540 nm. All experiments were independently carried out with three replicates.

Impacts of temperature, pH, and organic solvent on the activity of STPE-PMNSs. Aliquots of STPE-PMNSs (100 μg mL^{-1}) and GS (100 μg mL^{-1}) were pretreated in ddH_2O at 20-60 °C, 10 mM phosphate buffers at pH 3-11 or different ratios of DMF, DMSO, MeOH, MeCN solutions (0%, 10%, 20%, 30%, 40%, 50%, 60%, 70%, 80%) for 2 h. Then, the treated GS and STPE-PMNSs samples were collected by ultrafiltration (3000 Da) and resuspended in solutions containing Glu, NH_4^+ and ATP for 2 h reactions. After that, the absorbance in 540 nm at different conditions was examined. All experiments were independently carried out with three replicates.

Calculation of the apparent Michaelis-Menten constant of STPE-PMNSs and natural GS. The measurements of the apparent *Km* of STPE-PMNSs were performed

at 37 °C in HEPES buffer (100 mM, pH 7.3). To measure the apparent *Km* of STPE-PMNSs for Glu, we performed the reactions at growing concentrations of Glu, from 0 to 20 mM, while keeping the STPE-PMNSs, NH_4^+ and ATP concentration constant (100 μg mL^{-1}, 20 mM and 20 mM, respectively). An equivalent experiment was carried out to determine the apparent *Km* for NH_4^+ and ATP. For that, we varied the NH_4^+ or ATP concentration from 0 to 20 mM while holding the STPE-PMNSs, Glu, and ATP or NH_4^+ concentrations to 100 μg mL^{-1}, 20 mM, and 20 mM, respectively.

For natural GS, the apparent *Km* was determined in reaction mixtures consisting of 100 mM imidazole-HCl, 40 mM Glu, 25 mM 2-mercaptoethanol, 20 mM ATP, 20 mM $MgCl_2$, and 40 mM NH_4Cl. Stock solutions of imidazole, Glu, and ATP were titrated to pH 7.2. All reagents were stored on ice before assaying. When Glu was varied, NH_4^+ and GS were held constant at 40 mM and 100 μg mL^{-1}, respectively. An equivalent experiment was carried out to determine the apparent *Km* for NH_4^+ and ATP. For that, we varied the NH_4^+ or ATP concentration from 0 to 40 mM while holding the STPE-PMNSs, Glu, and ATP or NH_4^+ concentrations to 100 μg mL^{-1}, 40 mM, and 40 mM, respectively. In both cases enzyme-like first order kinetic behavior was observed.

^{1}H NMR analysis. STPE-PMNSs (1 mg) were added to 1 mL of D_2O containing 40 mM NH_4^+ and 40 mM Glu. After a certain time of reaction, the STPE-PMNSs were separated by an ultrafiltration membrane (MWCO at 3000 Da, UFC500396, Millipore) at 4000 g 4 °C for 20 min. Afterward, 500 μL of supernatant was removed to measure ^{1}H NMR.

Competitive experiments of Glu activation by ATP and polyP. STPE-PMNSs (100 μg mL^{-1}) were added to in HEPES buffer (100 mM, pH 7.3). We performed the catalytic reactions at growing concentrations of ATP, from 0 to 20 mM, while keeping the STPE-PMNSs, NH_4^+ and Glu concentration constant (100 μg mL^{-1} 20 mM and 20 mM, respectively). After reaction 2 h, we measured changes in fluorescence emission at 467 nm. All experiments were independently carried out with three replicates.

Computation method. In the calculation model without ATP, DFT calculations were constructed and implemented in the Vienna ab initio simulation package (VASP. 6.3.0)[4,5]. Using the electron exchange and correlation energy treated within the generalized gradient approximation in the Perdew−Burke−Ernzerhof functional (GGA-PBE)[6], the calculations were performed with a plane-wave basis set defined by a kinetic energy cutoff of 450 eV. The long-range dispersion interactions between adsorbates and surface were treated by applying the DFT-D3 method developed by Grimme et al[7]. The k-point sampling was obtained from the Monkhorst−Pack scheme with a (3×3×1) mesh for optimization and electronic structure. The geometry optimization and energy calculation were finished when the electronic self-consistent iteration and force reached 10–5 eV and 0.02 eV $Å^{-1}$, respectively.

For the calculation model in the presence of ATP, the geometry optimizations of the cluster models were performed at r2SCAN-3c[8] level using ORCA 5.0.3[9] considering the balance between the computational load and the accuracy. The performance of the currently chosen method is verified to be suitable for the adsorption on polar salt and non-polar coinage-metal surfaces with considerably lower cost. The Conductor-like Polarizable Continuum Model (CPCM)[10] was used to describe the solvent effect with taking water as solvent. The edged atoms were freeze during the geometric relaxation. Single point energy calculations were done at PBE/def2-SVP[11-13]+CPCM (H_2O) level with Gaussian 16[14]. The energetic difference (ΔE) is calculated by: $\Delta E = E^{\mathbf{1+ads}} - E^{\mathbf{1}} - E^{\mathbf{ads}}$, where $E^{\mathbf{1+ads}}$ refer to the total energy after adsorbs and $E^{\mathbf{1}}$ and $E^{\mathbf{ads}}$ refers to the energy of **1** and adsorbents, respectively.

Cell culture. Human neuroblastoma cells (SH-SY5Y) were authenticated by a unique STR profile, and the results indicated no cross-contamination. SH-SY5Y cells were immersed in MEM/F12 (GIBCO, NY, USA) containing 10% fetal bovine serum (FBS, GIBCO) and 1% penicillin/streptomycin (GIBCO). All cell culture systems were incubated at 37 °C in a humidified and 5% CO_2 environment.

Cell viability assay. SH-SY5Y cells were seeded in 96-well plates at a density of 1×10^4

cells/well. After 24 h of incubation, 100 μL culture medium containing STPE-PMNSs were added to each well. Following another 24-hour incubation, 10 μL of CCK8 solution was added to each well. After incubating for 4 hours, the absorbance at 450 nm was measured. All experiments were independently carried out with three replicates.

***In Vitro* Cellular Uptake of STPE-PMNSs.** We conducted a quantitative analysis of the internalization of STPE-PMNSs by cells over a defined time interval. SH-SY5Y cells (1.2×10^6 cells mL^{-1}) were seeded in 6-well tissue culture plates and grown to ~90% confluence. Growth media in plates was replaced with media containing STPE-PMNSs. Cells were incubated with nanomaterials for 0, 3 h, 6 h, 12 h, 18 h, and 24 h at 37 °C. Finally, the medium was removed from the wells, and the cells were washed three times with PBS to clear away nanomaterials outside of the cells. Next, 200 μL of 1% Triton X-100 in 0.1M NaOH solution was added to lyse the cells. After treatment, Mn content in the lysates was determined using ICP–OES. All experiments were independently carried out with three replicates.

ICP-OES/MS Measurement. Transfer 50 μL of prepped cell lysate into a 1.5 mL Eppendorf tube, followed by the addition of 100 μL of concentrated nitric acid for digestion at 95℃ for 1 hour. After cooling, introduce 50 μL of H_2O_2 and maintain at 95℃ for 30 minutes. Subsequently, adjust the volume to 1 mL with ddH_2O and measurement of Mn content on an inductively coupled plasma optical emission spectrometry/mass spectrometer.

Intracellular imaging of STPE-PMNSs. For CLSM, SH-SY5Y cells were first seeded onto 2-cm culture dishes at a density of 8.0×10^4 cells mL^{-1} for 24 h. Then the culture medium was changed with as-prepared DMEM containing STPE-PMNSs (25 μg mL^{-1}). The cells were incubated with nanomaterials for 6 h at 37 °C, and then the medium was removed from the dish. After rinsing with PBS three times to remove residual nanomaterials, the cells were incubated with 1 mM 4-AP (induces cells to produce Glu)

for 4 h at 37 °C. Subsequently, the culture medium was discarded, and after washing 3 times with PBS, the cells were monitored using a confocal laser scanning microscope with a living cell CO_2 culture system. (CLSM, OLYMPUS SpinSR). All experiments were independently carried out with three replicates.

Bio-TEM imaging. SH-SY5Y cells were cultivated to obtain 1.0 × 10^7 cells. Then, the cells were treated with 25 μg mL^{-1} STPE-PMNSs for 24 h. After rinsing three times with PBS to remove the residual STPE-PMNSs, cells were collected and transferred to fixative, stained, sliced and imaged by TEM (Hitachi HT7700) according to previous reports[15].

Apoptosis analysis. SH-SY5Y cells were seeded in 6-well tissue culture plates at a density of 1.5 ×10^5 cells mL^{-1} and set up in CK, 4-AP, STPE-PMNSs, and STPE-PMNSs + 4-AP groups, respectively, and after 24 h of incubation, the cells were treated in the STPE-PMNSs group and STPE-PMNSs + 4-AP group with 25 μg mL^{-1} of STPE-PMNSs for 24 h, after which the cells were washed three times with PBS, and 4-AP was added to the STPE-PMNSs + 4-AP group for 4 h. The cells were collected, washed, and incubated with *Annexin V-FITC* and *PI* according to the instructions of the *Annexin V-FITC* Apoptosis Detection Kit (KeyGEN). Then, the cells were further washed and analyzed by flow cytometry (BD FACSCalibur). All experiments were independently carried out with three replicates.

Glu and Gln levels. SH-SY5Y cells were seeded in 6-well tissue culture plates at a density of 1.5 ×10^5 cells mL^{-1} and set up in CK, 4-AP, STPE-PMNSs, and STPE-PMNSs + 4-AP groups, respectively, and after 24 h of incubation, the cells were treated in the STPE-PMNSs group and STPE-PMNSs + 4-AP group with 25 μg mL^{-1} of STPE-PMNSs for 24 h, after which the cells were washed three times with PBS, and 4-AP was added to the STPE-PMNSs + 4-AP group for 4 h. The cells were collected, and then the cell lysates were obtained with a Whole Cell Lysis Assay (KeyGEN BioTECH Co., Ltd. Nanjing, China). The Glu and Gln contents were determined using the

corresponding content assay kit. Both of their contents were determined by comparing the absorbance value with the calibration plot for standard solutions. The absorbance values were measured at 340 nm and 450 nm. All experiments were independently carried out with three replicates.

Western blot analysis. SH-SY5Y cells were seeded in 6-well tissue culture plates at a density of 1.5 $\times 10^5$ cells mL^{-1} and set up in CK, 4-AP, STPE-PMNSs, and STPE-PMNSs + 4-AP groups, respectively, and after 24 h of incubation, the cells were treated in the STPE-PMNSs group and STPE-PMNSs + 4-AP group with 25 μg mL^{-1} of STPE-PMNSs for 24 h, after which the cells were washed three times with PBS, and 4-AP was added to the STPE-PMNSs + 4-AP group for 4 h. The cells were collected, and then the cell lysates were obtained with a Whole Cell Lysis Assay. The protein concentration was determined by the Super-Bradford Protein Assay Kit (CWBiotech, Inc., Beijing, China). Briefly, the extracts were first separated by 10% SDS–PAGE and transferred to a polyvinylidene difluoride membrane (Bio-Rad, CA, USA). The membrane was blocked with 5% BSA in TBST at 25 °C for 1 h and then incubated with antibodies at 4 °C overnight. The expression of α-actin was used as the internal standard. Primary antibodies against the following proteins were used: α-actin (1:1000 for WB), Bcl-2 (1:1000 for WB), and Bak (1:1000 for WB). The appropriate secondary antibodies (1:1000 for WB) were purchased from CST. Details are shown in the Source Data. All experiments were independently carried out with three replicates.

Ca^{2+} content measurement. SH-SY5Y cells were seeded in 6-well tissue culture plates at a density of 1.5 $\times 10^5$ cells mL^{-1} and set up in CK, 4-AP, STPE-PMNSs, and STPE-PMNSs + 4-AP groups, respectively, and after 24 h of incubation, the cells were treated in the STPE-PMNSs group and STPE-PMNSs + 4-AP group with 25 μg mL^{-1} of STPE-PMNSs for 24 h, after which the cells were washed three times with PBS, and 4-AP was added to the STPE-PMNSs + 4-AP group for 4 h. The cytosolic Ca^{2+} concentration was determined with Fluo-4 AM (Beyotime Biotechnology Co., Ltd, Shanghai, China) according to the manufacturer's procedure. The Ca^{2+} signals were analyzed by flow

cytometry. All experiments were independently carried out with three replicates.

Statistics and reproducibility. The Investigators were not blinded to allocation during experiments and outcome assessment. All multiple comparisons have been corrected before adjusted P values are presented. The tests used are indicated in figure legends.

Results

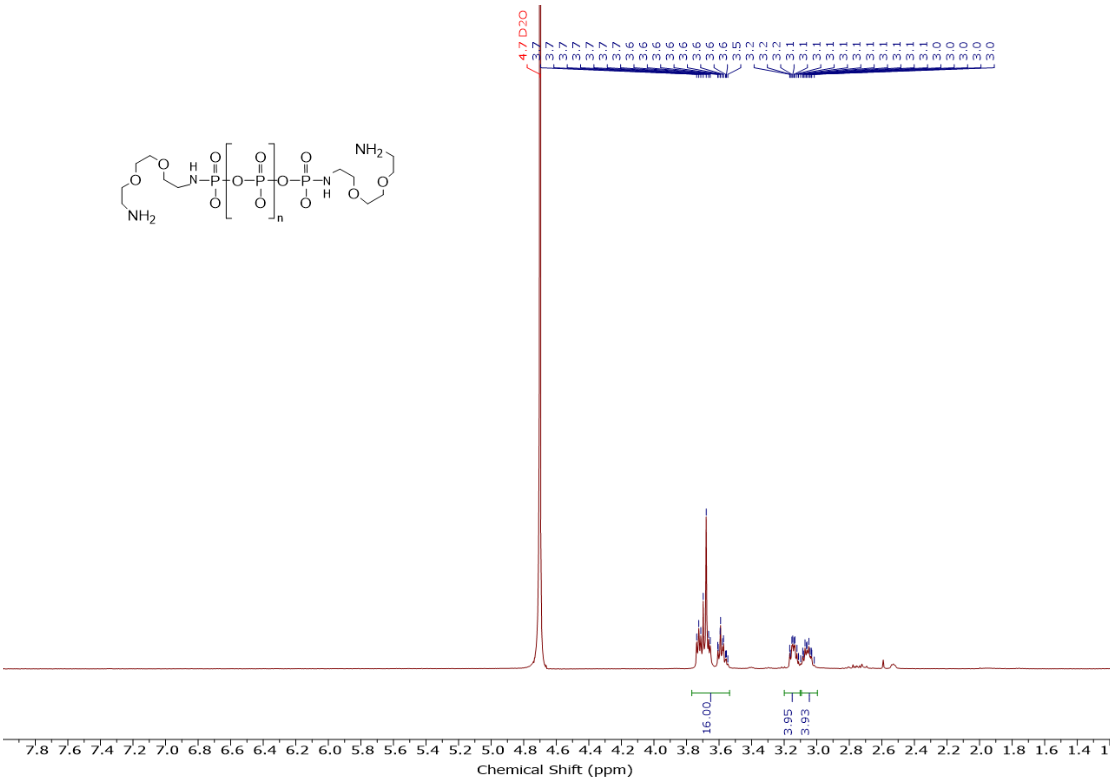

Supplementary Fig. 2 ^{1}H NMR spectrum of polyP-NH_2.

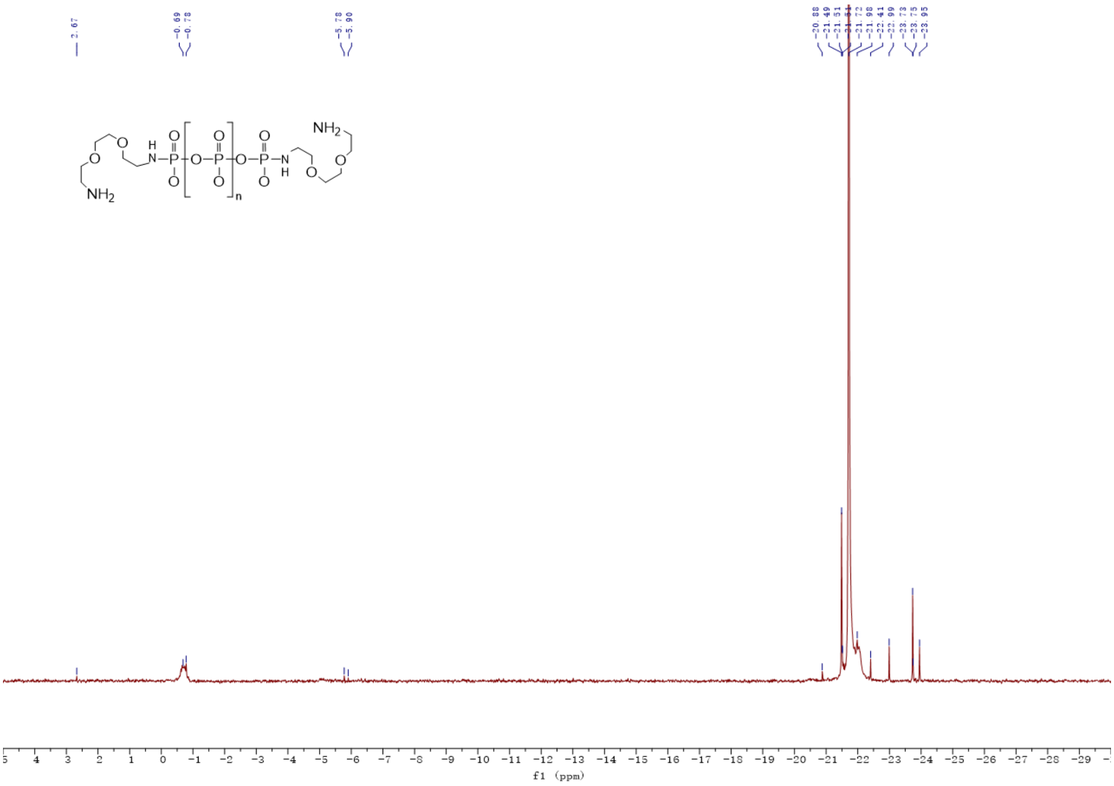

Supplementary Fig. 3 ^{31}P NMR spectrum of polyP-NH_2.

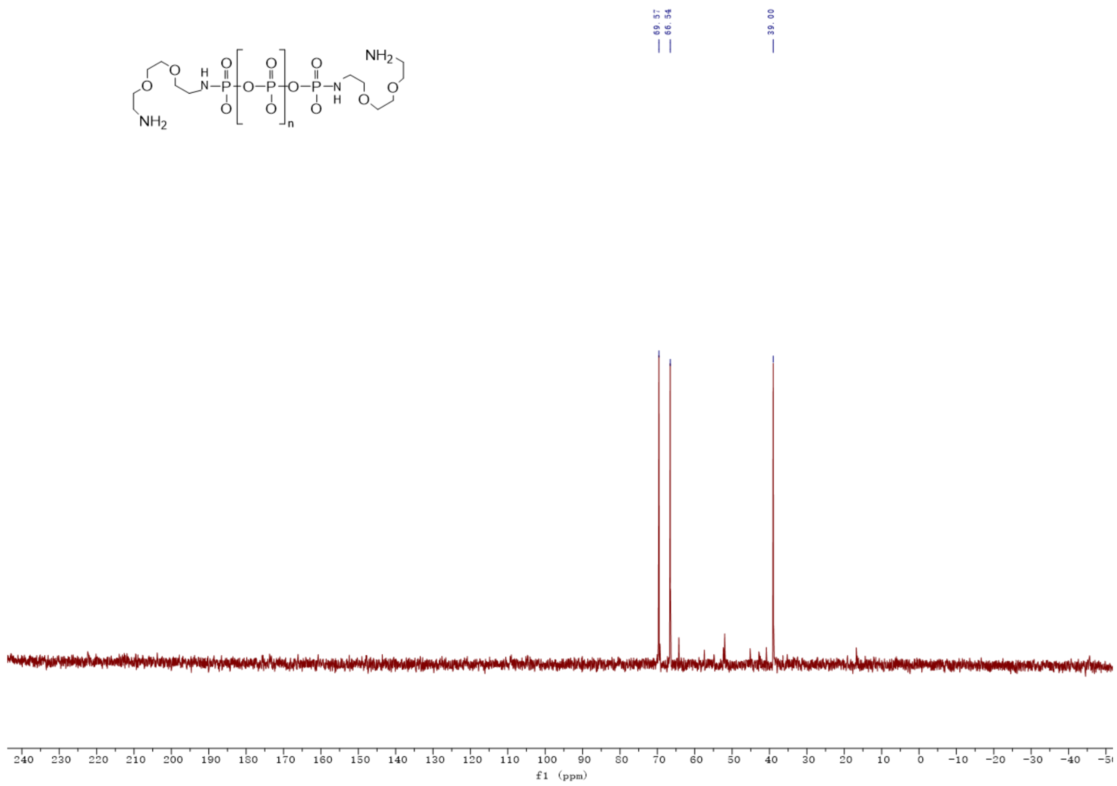

Supplementary Fig. 4 ^{13}C NMR spectrum of polyP-NH_2.

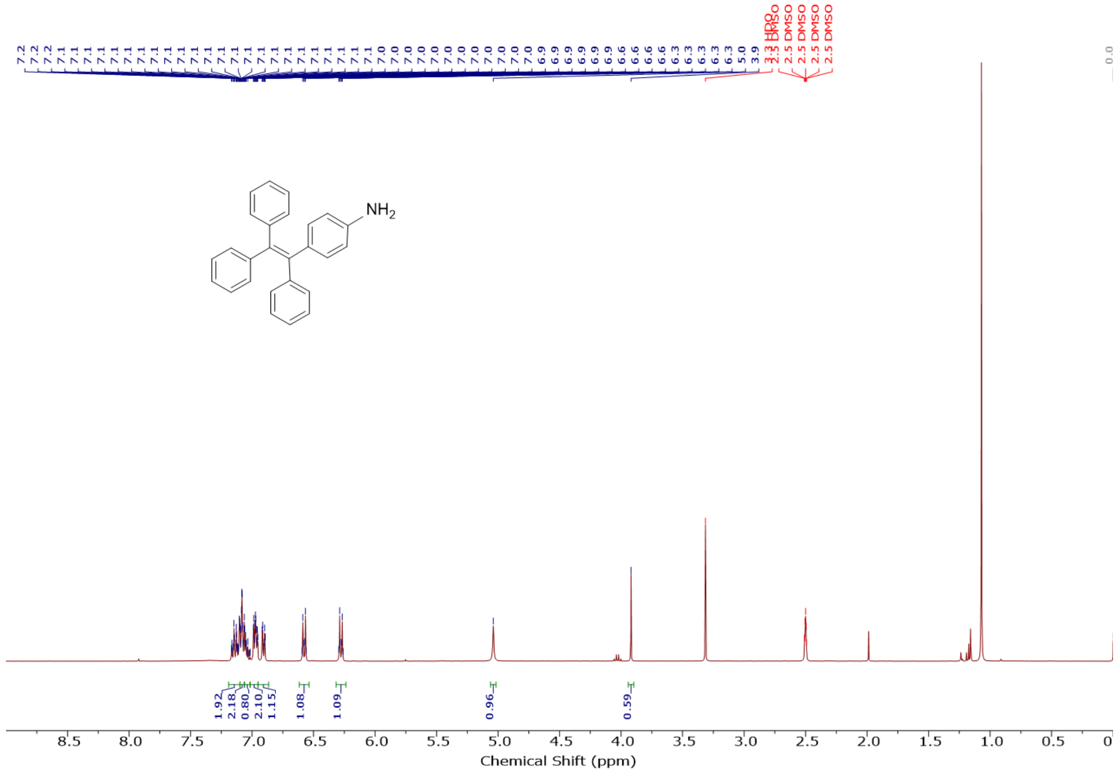

Supplementary Fig. 5 ^{1}H NMR spectrum of TPE-NH_2.

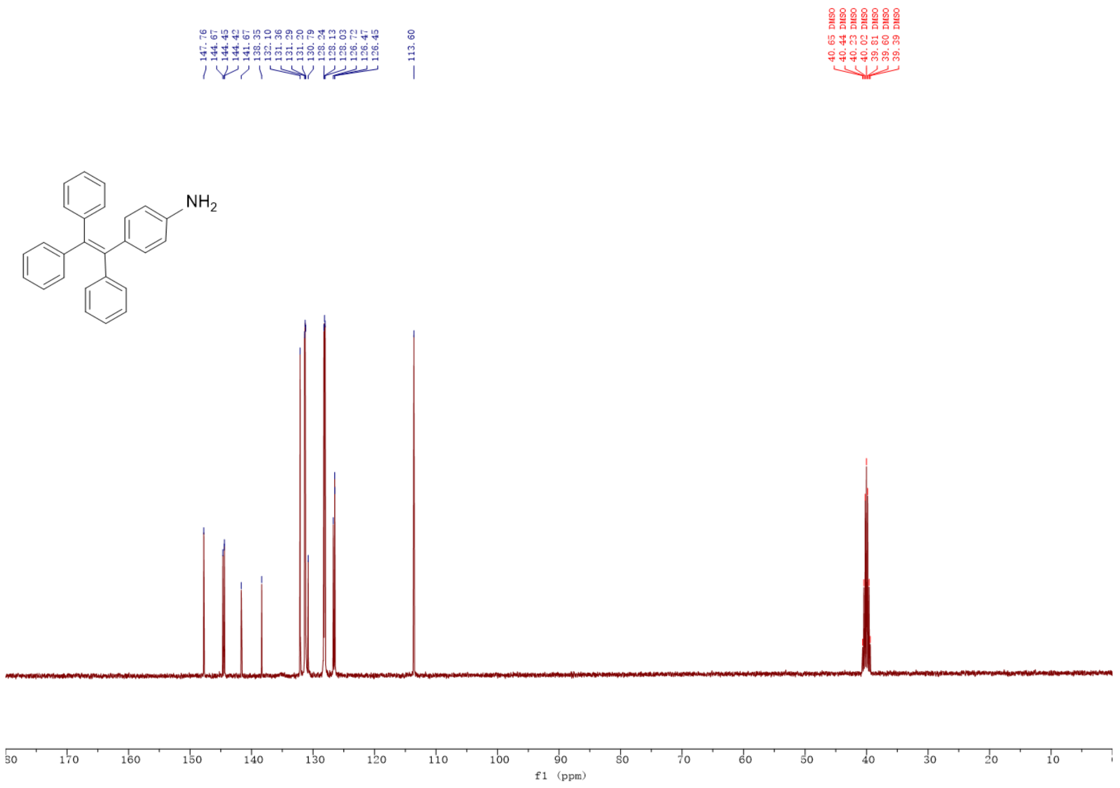

Supplementary Fig. 6 ^{13}C NMR spectrum of TPE-NH_2.

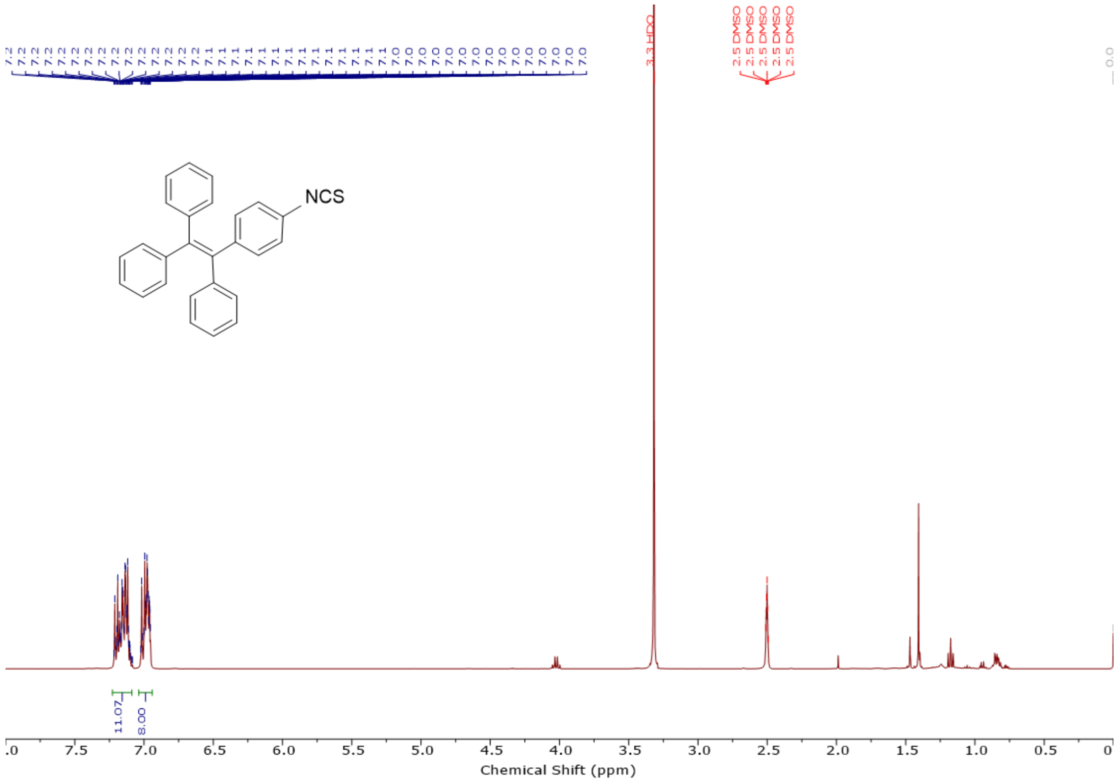

Supplementary Fig. 7 ^{1}H NMR spectrum of TPE-CNS.

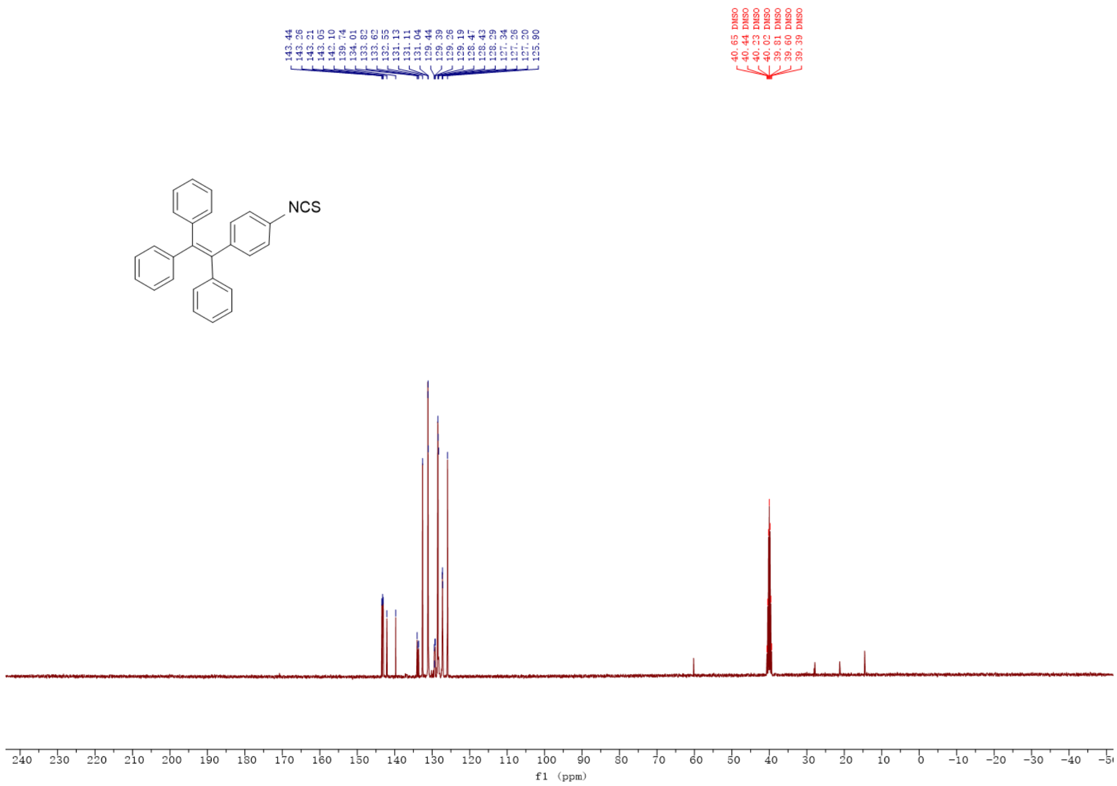

Supplementary Fig. 8 ^{13}C NMR spectrum of TPE-CNS.

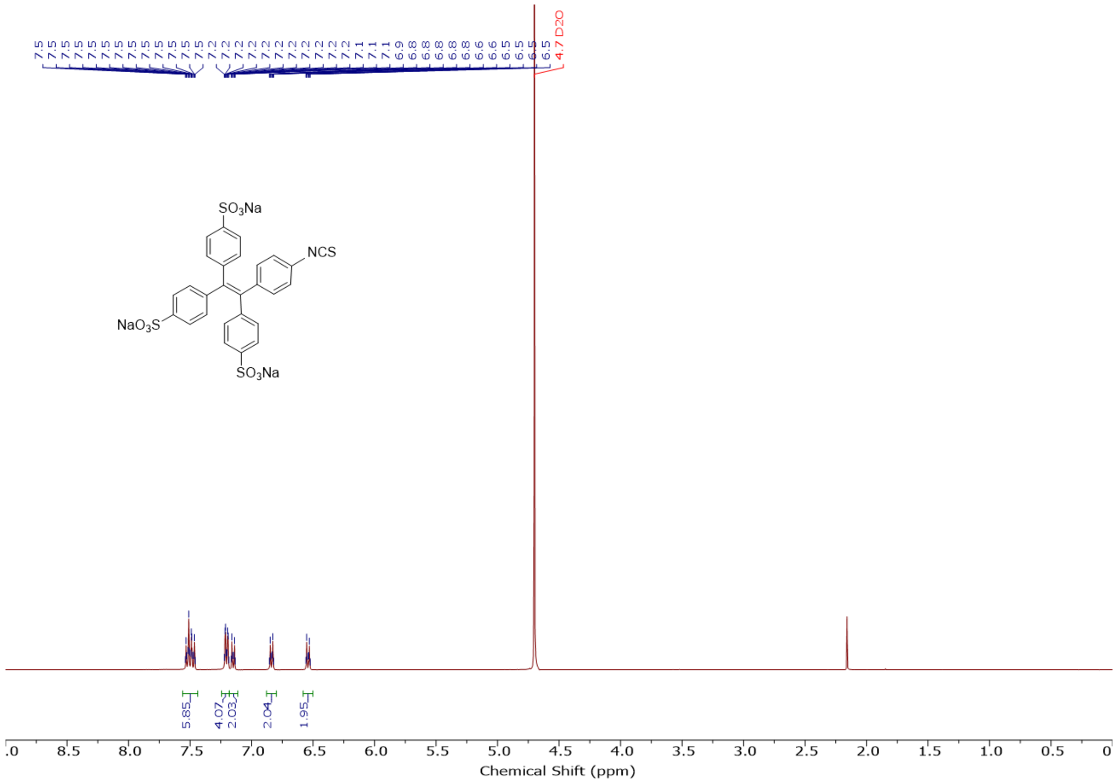

Supplementary Fig. 9 ^{1}H NMR spectrum of STPE.

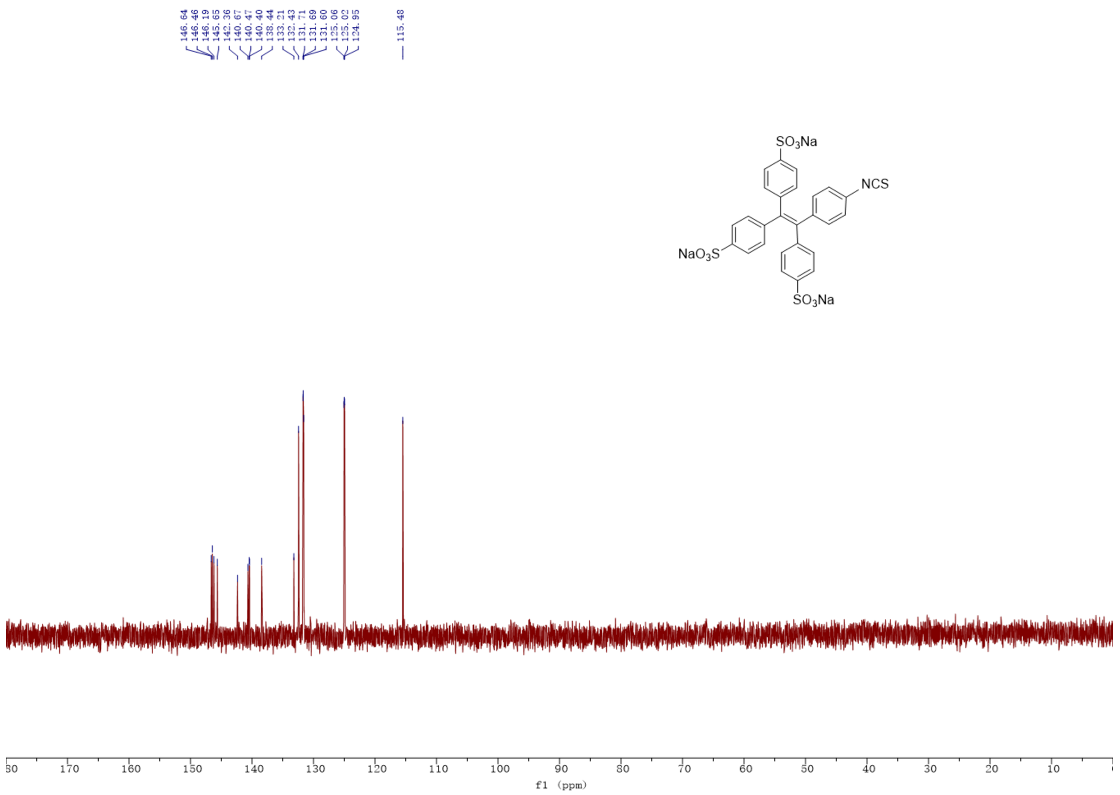

Supplementary Fig. 10 ^{13}C NMR spectrum of STPE.

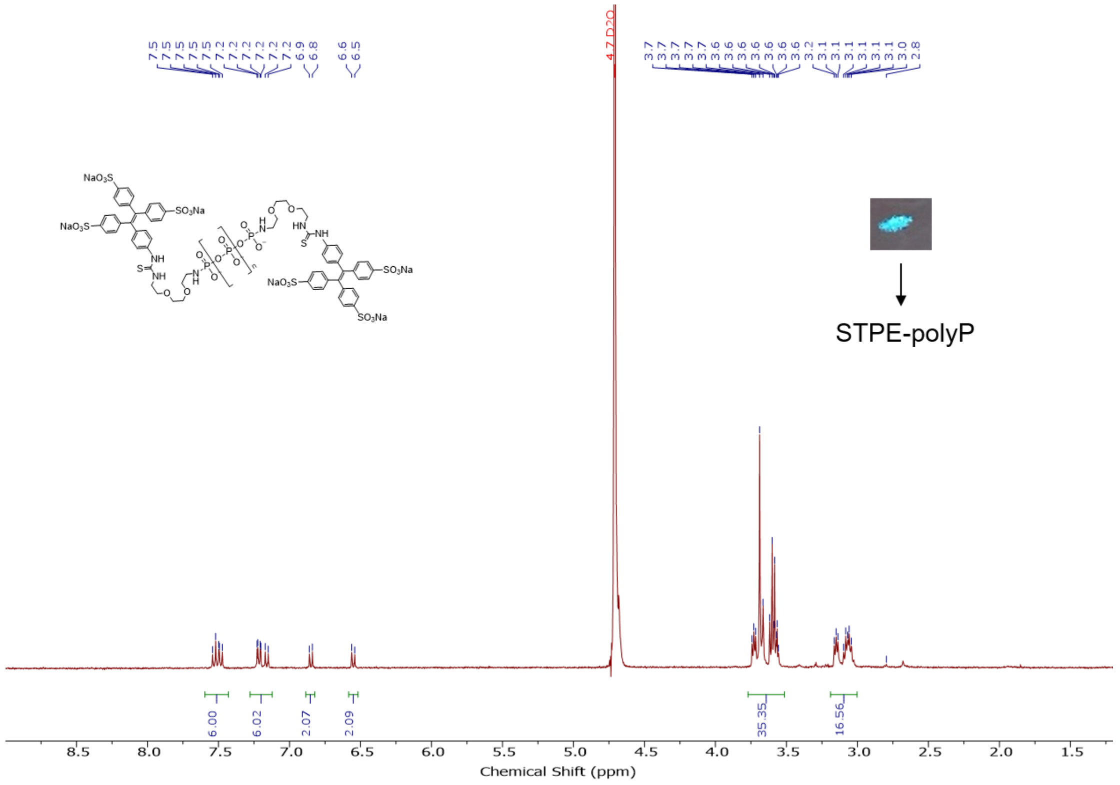

Supplementary Fig. 11 ^{1}H NMR spectrum of STPE-polyP.

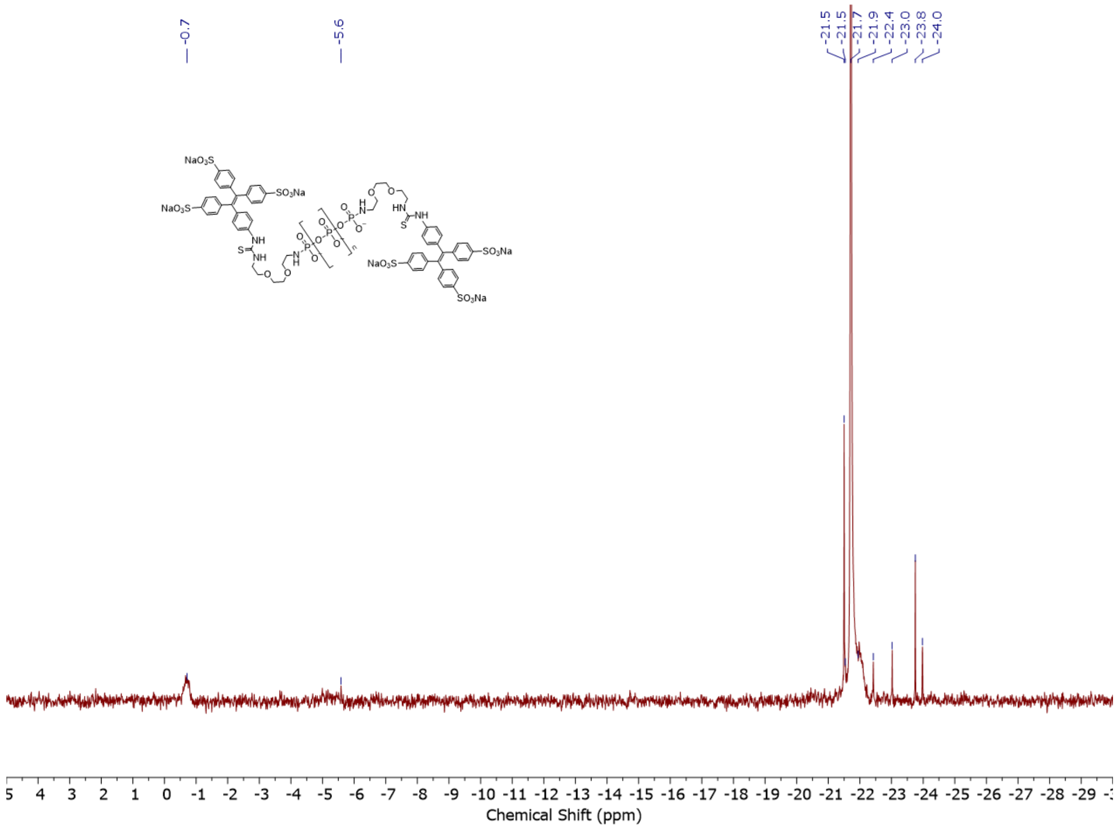

Supplementary Fig. 12 ^{31}P NMR spectrum of STPE-polyP.

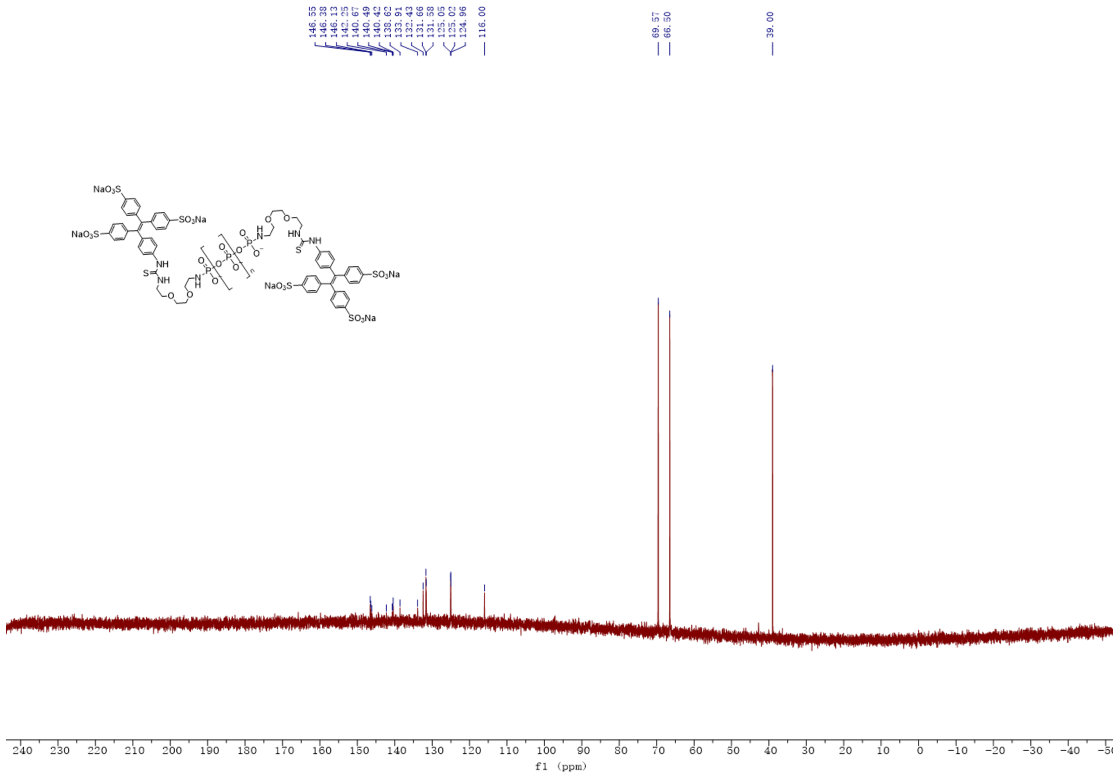

Supplementary Fig. 13 ^{13}C NMR spectrum of STPE-polyP.

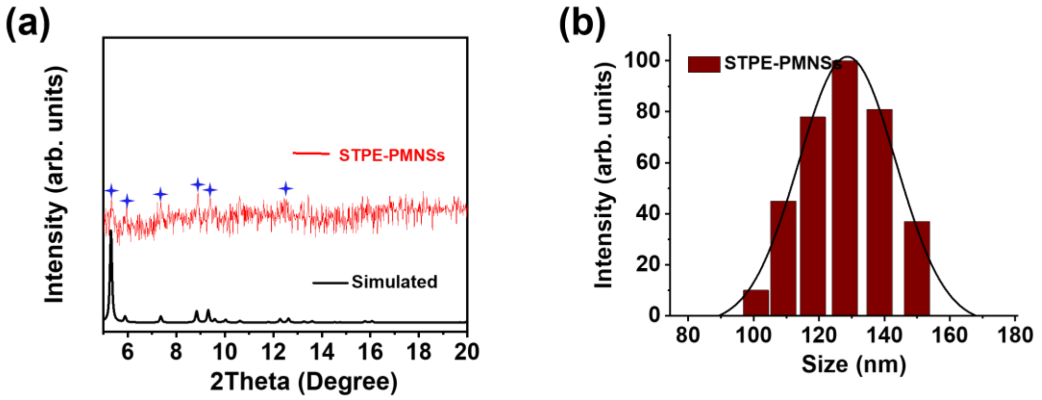

Supplementary Fig. 14 (a) XRD results of the STPE-PMNSs. (b) DLS analysis of the STPE-PMNSs. Experiments were repeated three times with similar results.

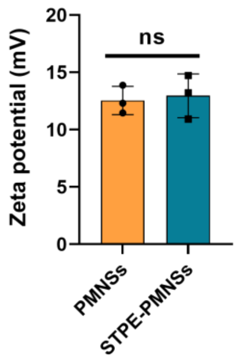

Supplementary Fig. 15 Comparison of zeta potential between PMNSs and STPE-PMNSs. Data points presented in this figure represent three (n=3) independent experiments for each experimental group and are displayed as mean ± standard deviation. *p* values were determined by a two-sided *t-test*. *p* value is 0.7699.

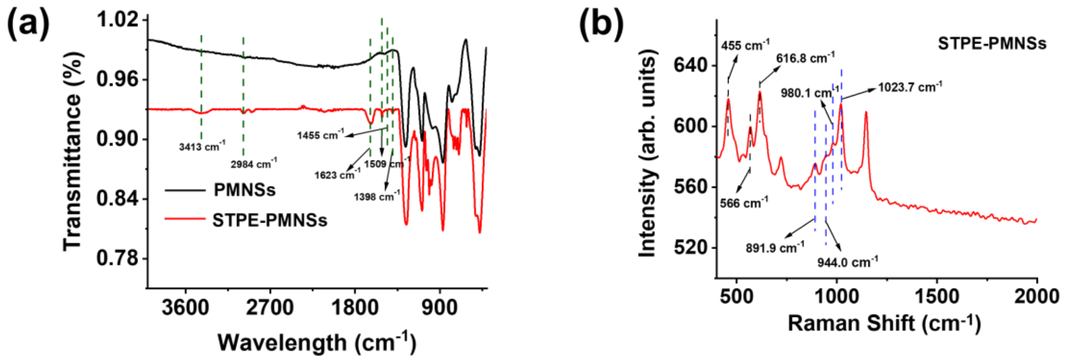

Supplementary Fig. 16 (a) Comparison of FT-IR spectroscopy between PMNSs and STPE-PMNSs. (b) Raman analysis of STPE-PMNSs. Experiments were repeated three

times with similar results.

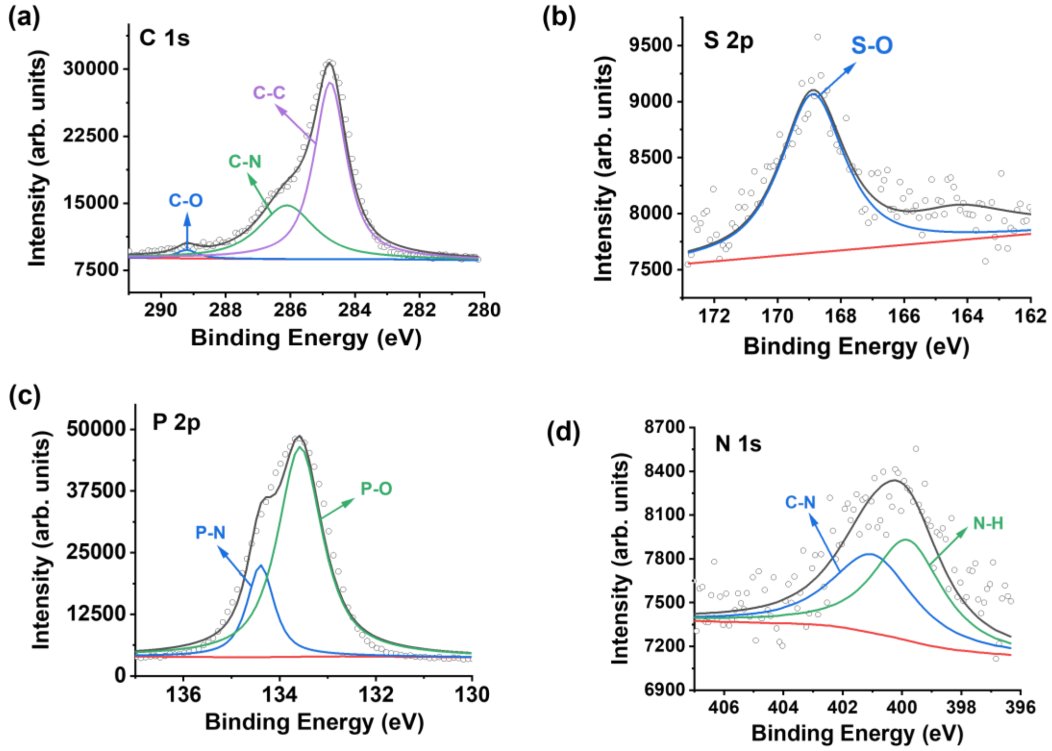

Supplementary Fig. 17 XPS analysis for C *1s* (a), S *2p* (b), P *2p* (c), N *1s* (d) of STPE-PMNSs. Experiments were repeated three times with similar results.

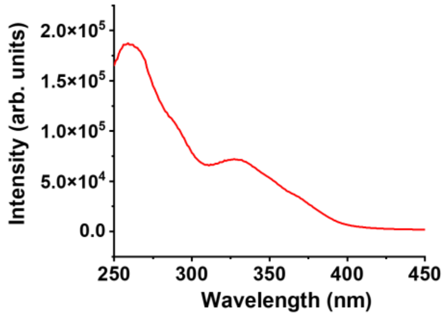

Supplementary Fig. 18 The excitation spectrum of STPE-PMNSs.

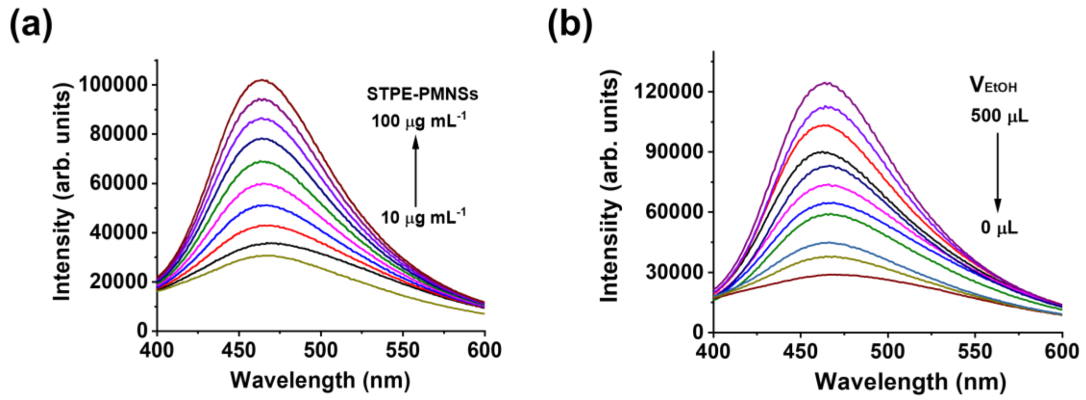

Supplementary Fig. 19 (a) The fluorescence emission spectrum of STPE-PMNSs (Ex = 330 nm). (b) The fluorescence emission spectrum of STPE-polyP (Ex = 330 nm). STPE-polyP exhibits non-emissive behavior in its solution state. To investigate its fluorescence emission spectrum in the aggregated state, the property of precipitation in ethanol is utilized for testing purposes. Experiments were repeated three times with similar results.

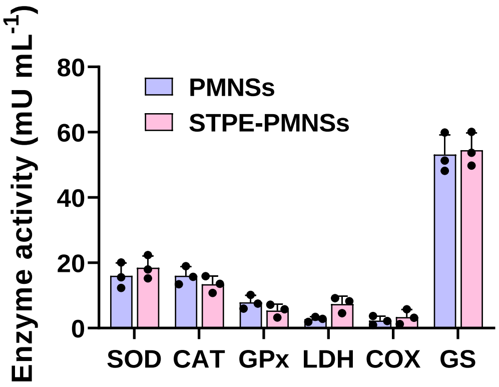

Supplementary Fig. 20 Enzyme activity of STPE-PMNSs for SOD, CAT, GPx, LDH, COX, GS. Data points presented in this figure represent three (n=3) independent

experiments for each experimental group and are displayed as mean ± standard deviation.

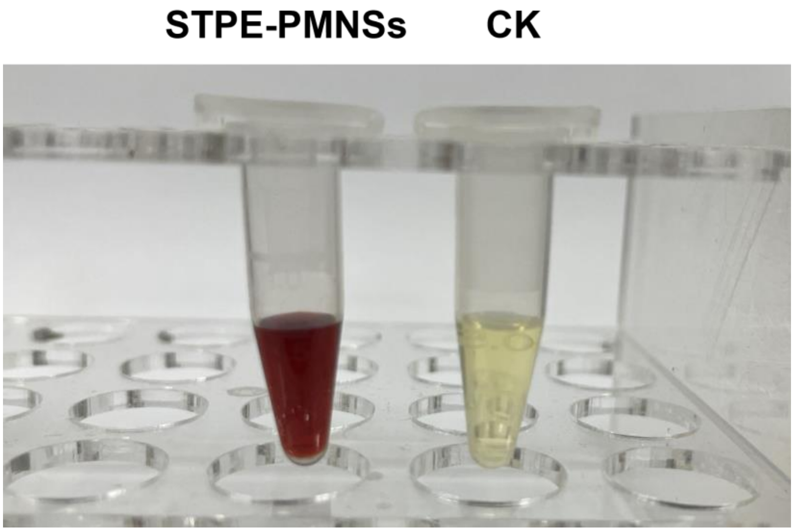

Supplementary Fig. 21 GS activity determination in the absence of ATP (STPE-PMNSs: 500 μg mL^{-1}). Experiments were repeated three times with similar results.

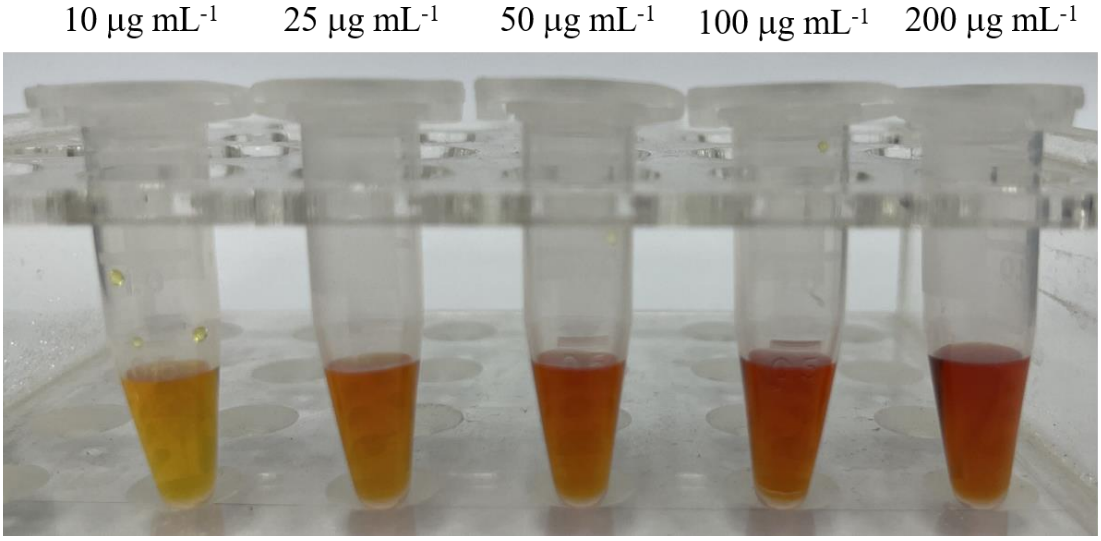

Supplementary Fig. 22 GS activity determination by colorimetric assay kits. Experiments were repeated three times with similar results.

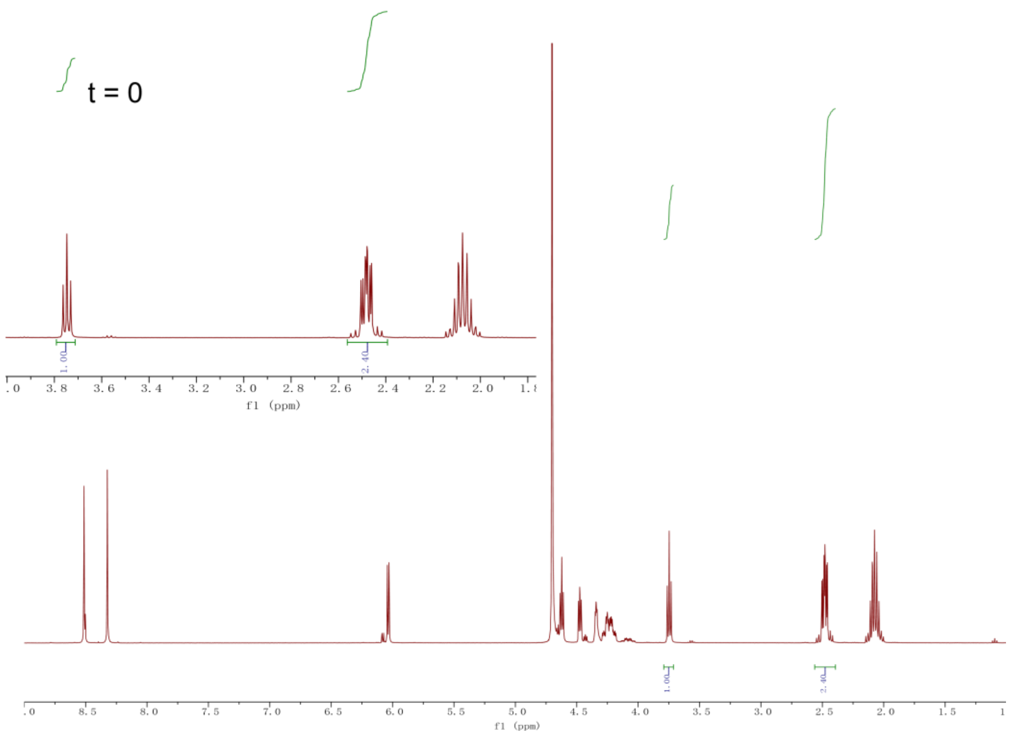

Supplementary Fig. 23 ^{1}H NMR spectrum monitoring the relative levels of Glu and Gln after reacting 0 min in the presence of ATP (20 mM).

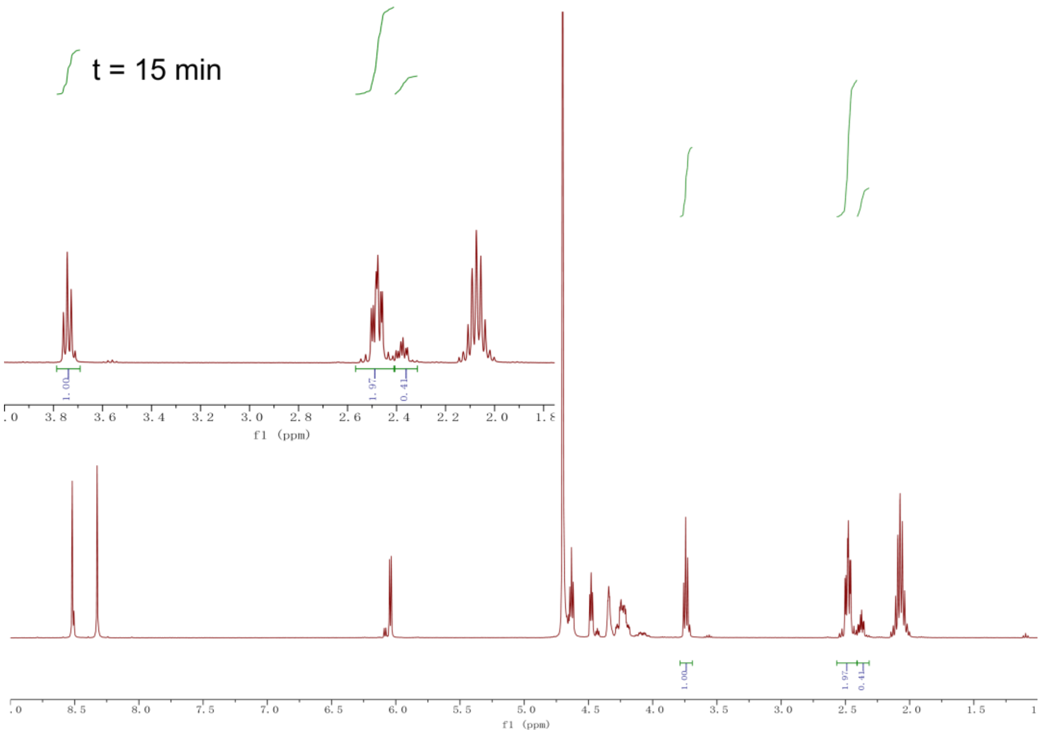

Supplementary Fig. 24 ^{1}H NMR spectrum monitoring the relative levels of Glu and Gln after reacting 15 min in the presence of ATP (20 mM).

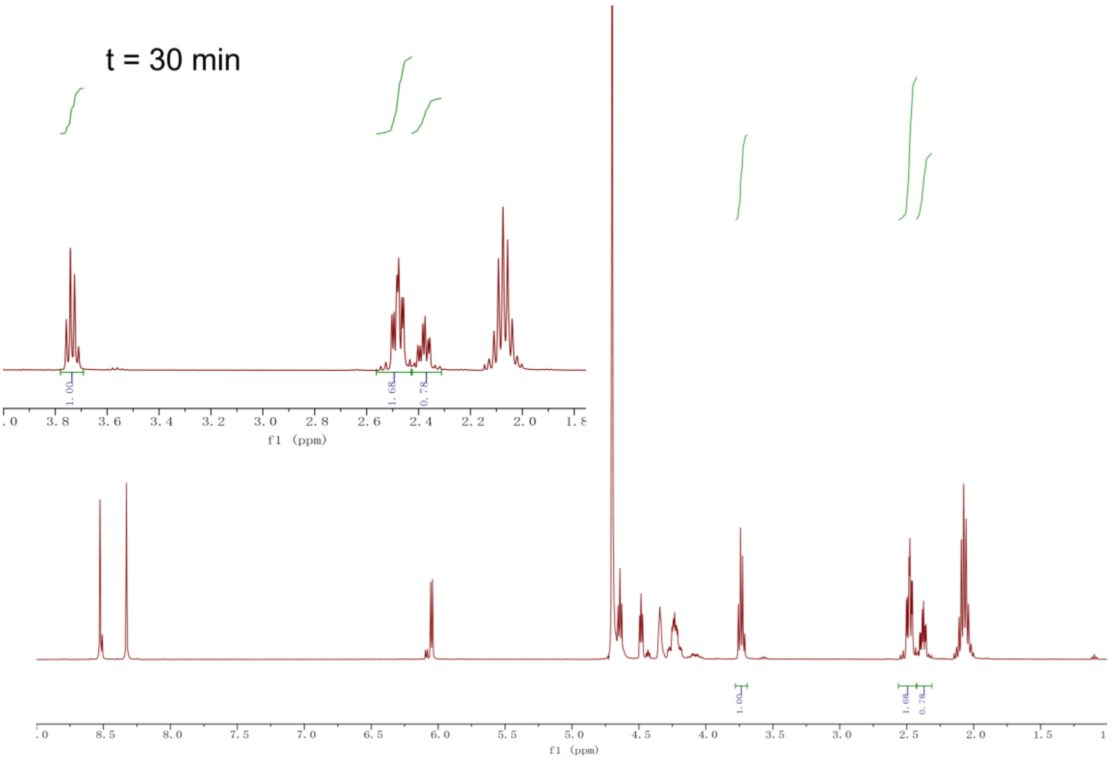

Supplementary Fig. 25 ^{1}H NMR spectrum monitoring the relative levels of Glu and Gln after reacting 30 min in the presence of ATP (20 mM).

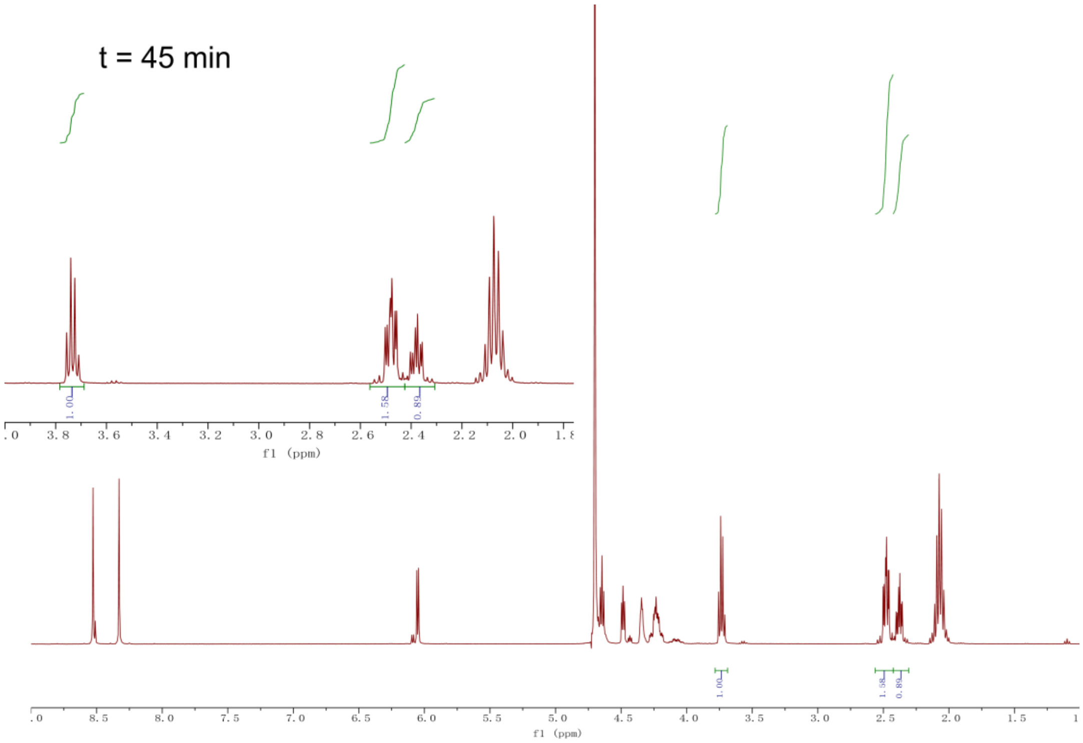

Supplementary Fig. 26 ^{1}H NMR spectrum monitoring the relative levels of Glu and Gln after reacting 45 min in the presence of ATP (20 mM).

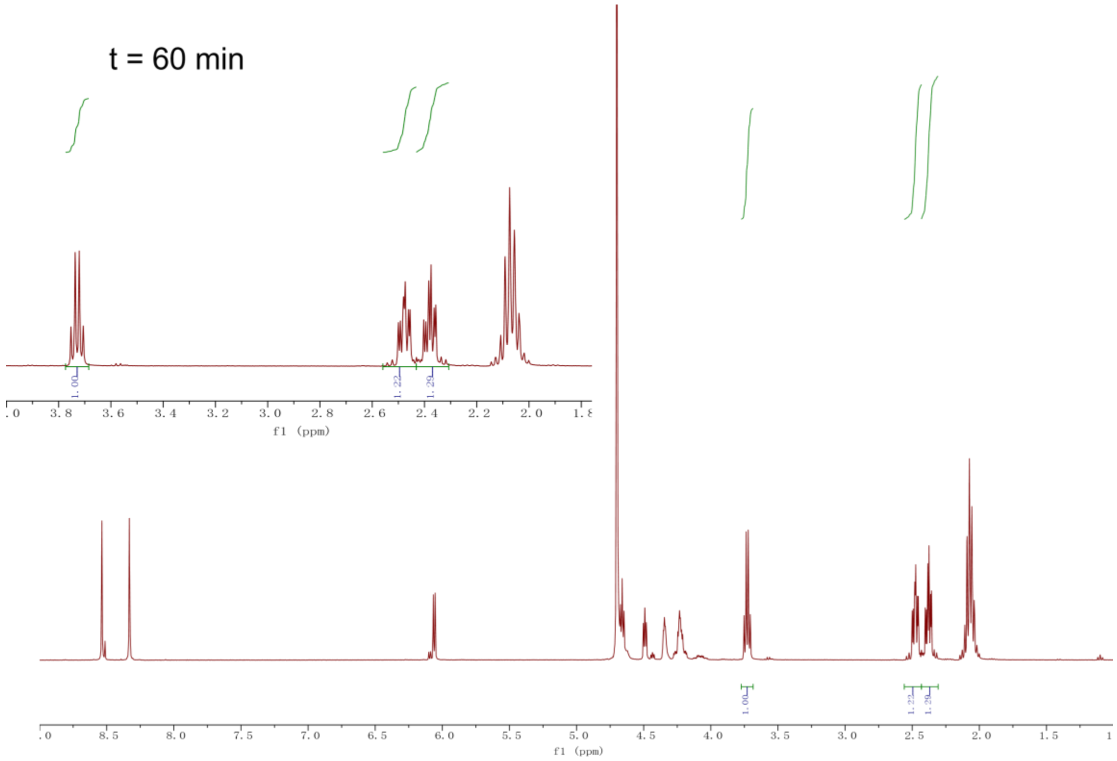

Supplementary Fig. 27 ^{1}H NMR spectrum monitoring the relative levels of Glu and Gln after reacting 60 min in the presence of ATP (20 mM).

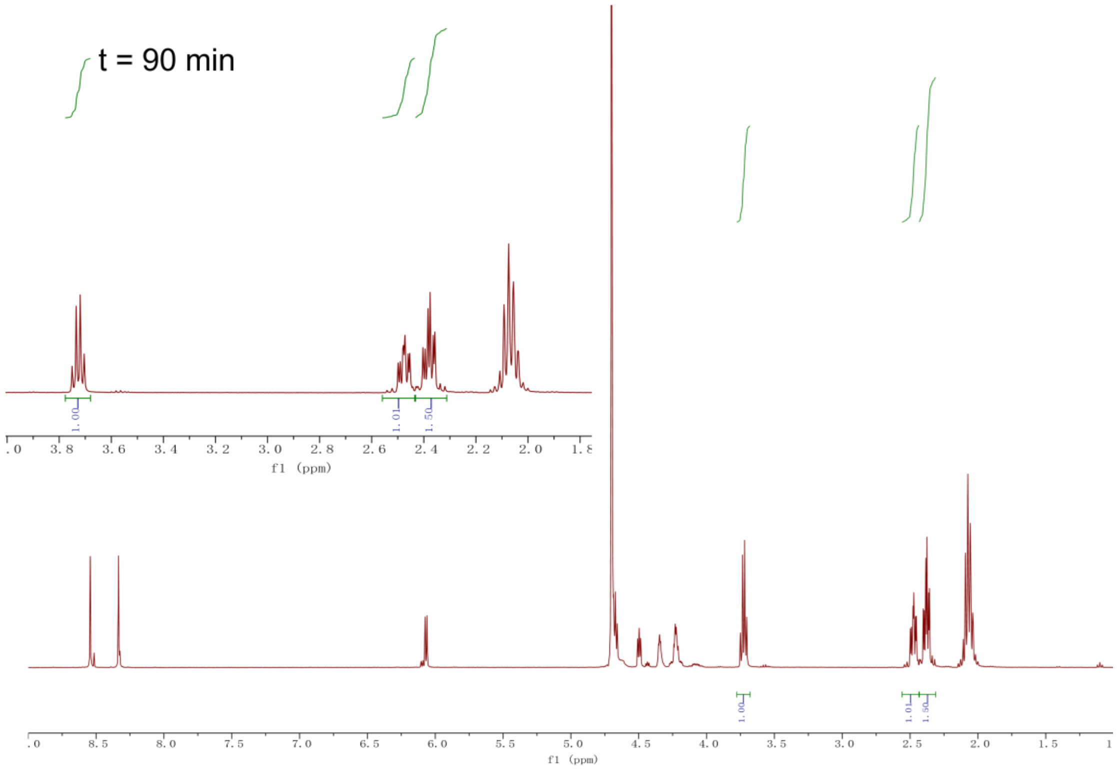

Supplementary Fig. 28 ^{1}H NMR spectrum monitoring the relative levels of Glu and Gln after reacting 90 min in the presence of ATP (20 mM).

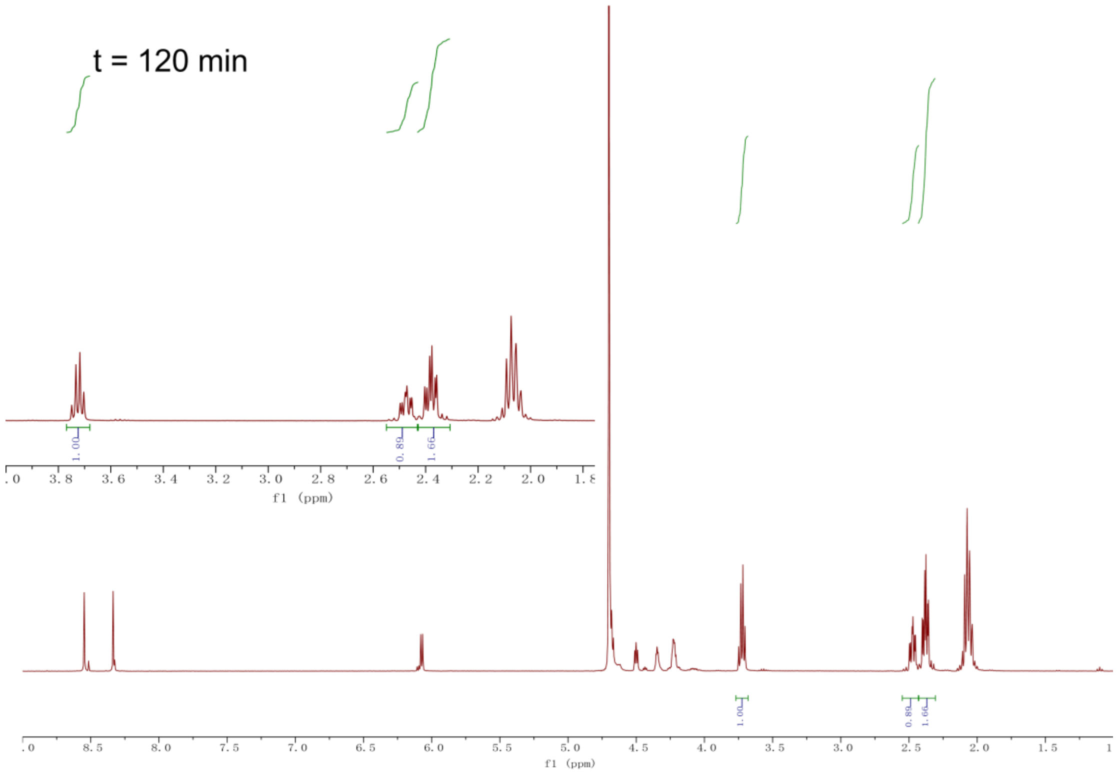

Supplementary Fig. 29 ^{1}H NMR spectrum monitoring the relative levels of Glu and Gln after reacting 120 min in the presence of ATP (20 mM).

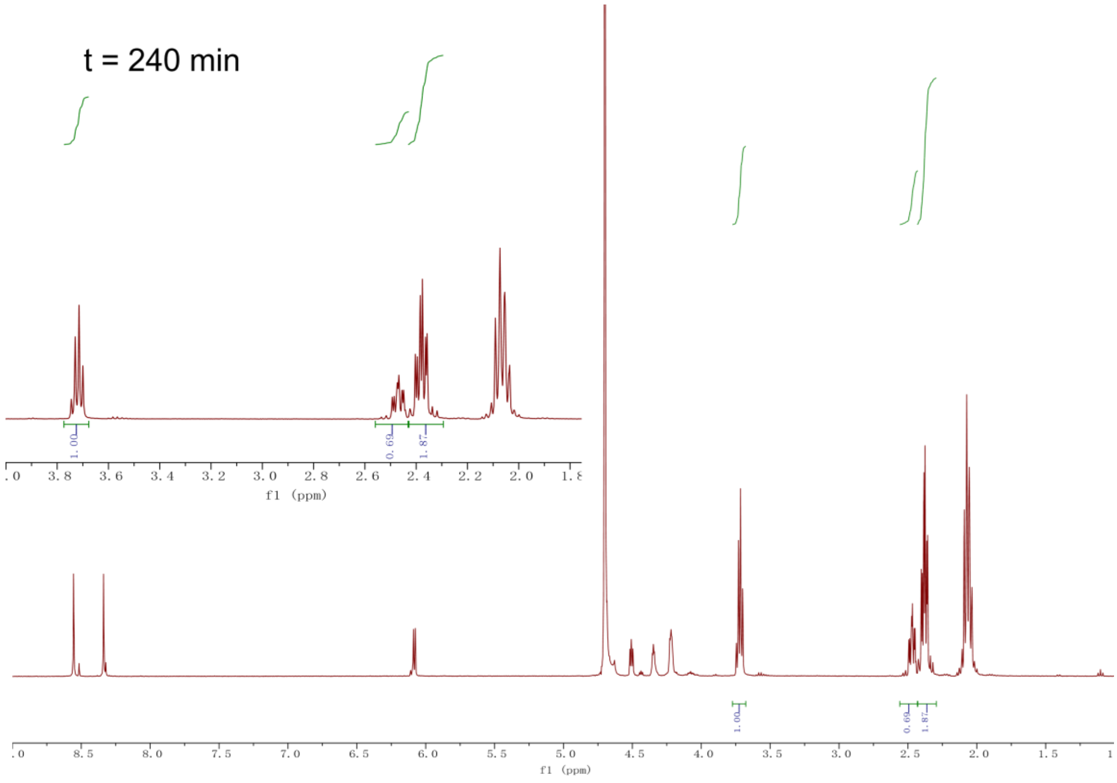

Supplementary Fig. 30 ^{1}H NMR spectrum monitoring the relative levels of Glu and Gln after reacting 240 min in the presence of ATP (20 mM).

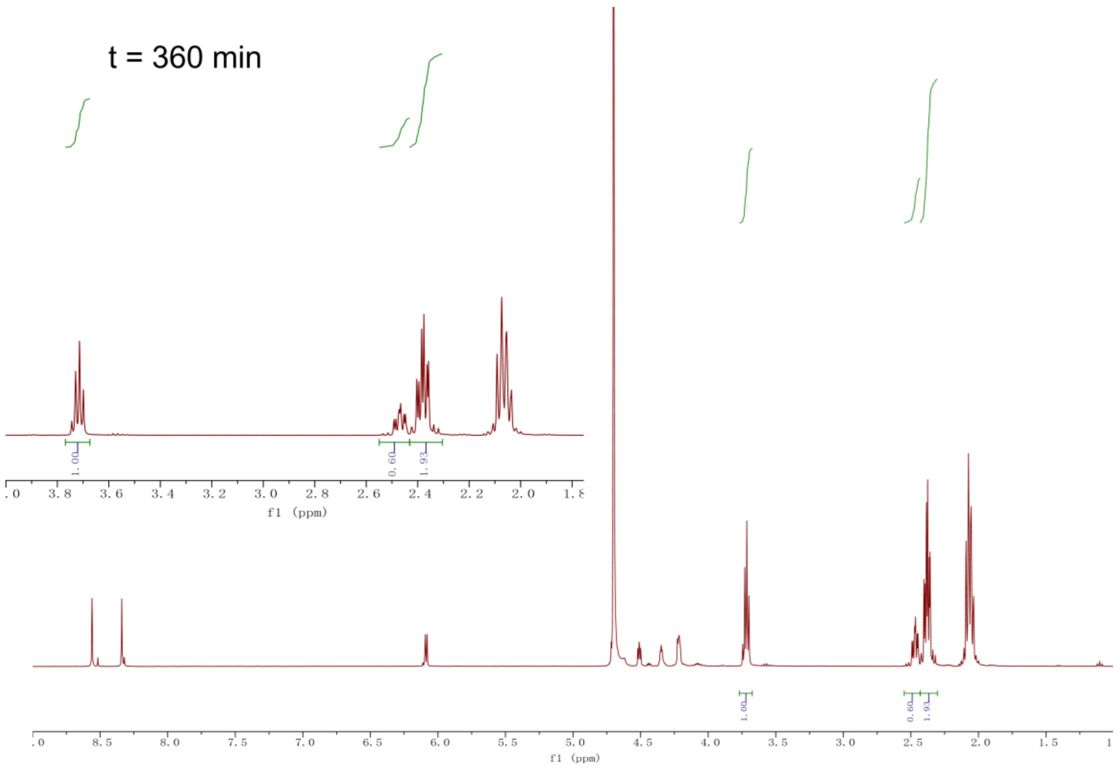

Supplementary Fig. 31 ^{1}H NMR spectrum monitoring the relative levels of Glu and Gln after reacting 360 min in the presence of ATP (20 mM).

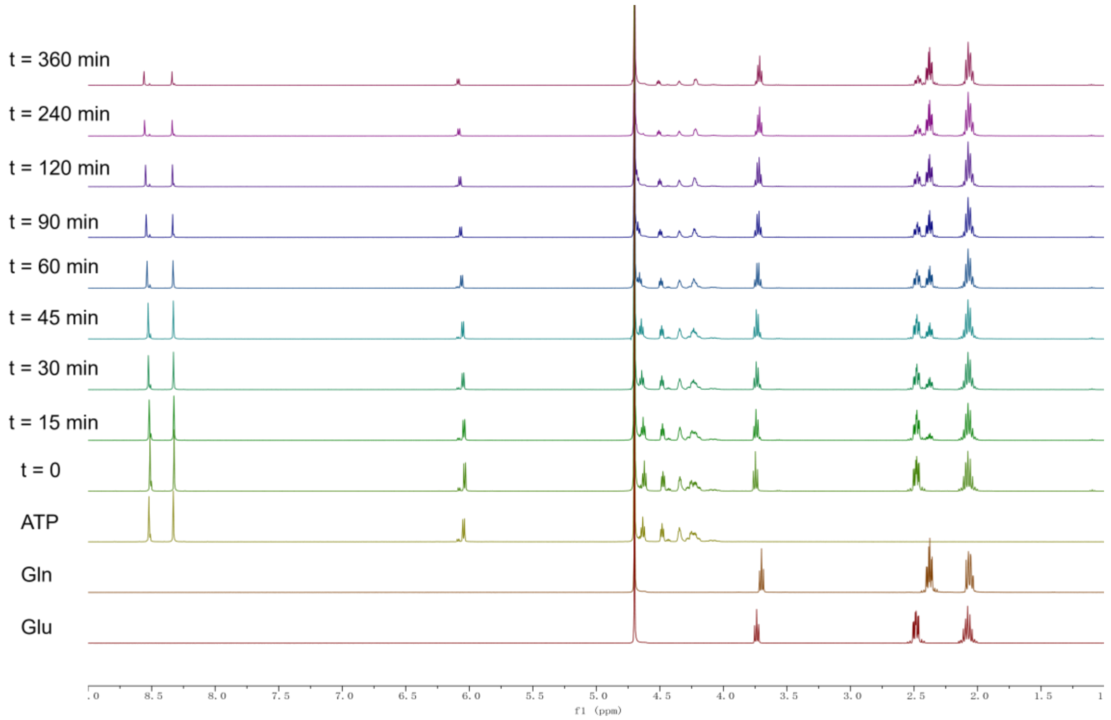

Supplementary Fig. 32 Merged ^{1}H NMR spectra from Supplementary Fig. 23 to Supplementary Fig. 31.

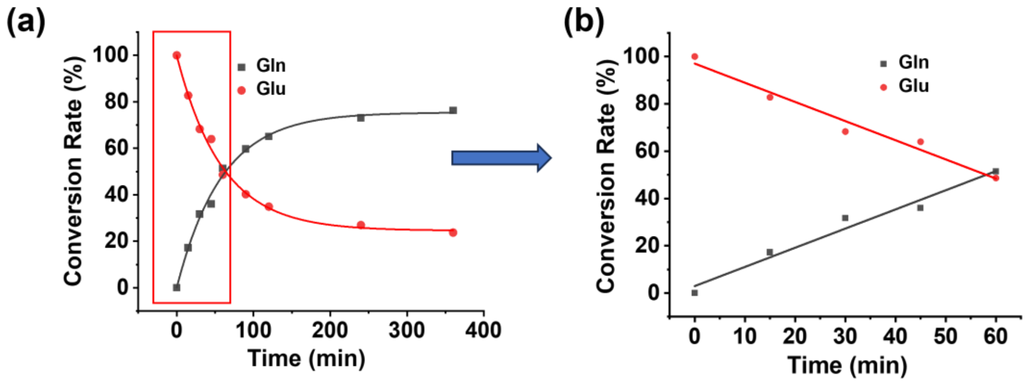

Supplementary Fig. 33 The results of NMR integrations from Supplementary Fig. 23-Supplementary Fig. 31. The conversion rates of Glu and Gln in the catalytic system (a) and a linear relation in the 0-60 min (b).

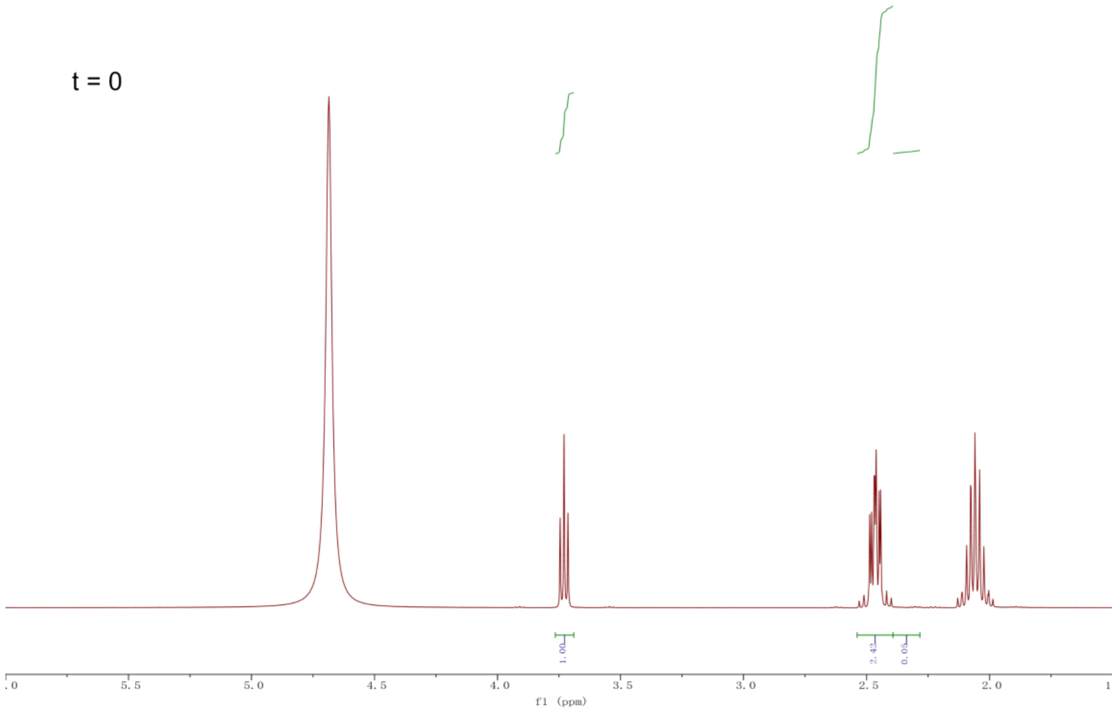

Supplementary Fig. 34 ^{1}H NMR spectrum monitoring the relative levels of Glu and Gln after reacting 0 min in the absence of ATP.

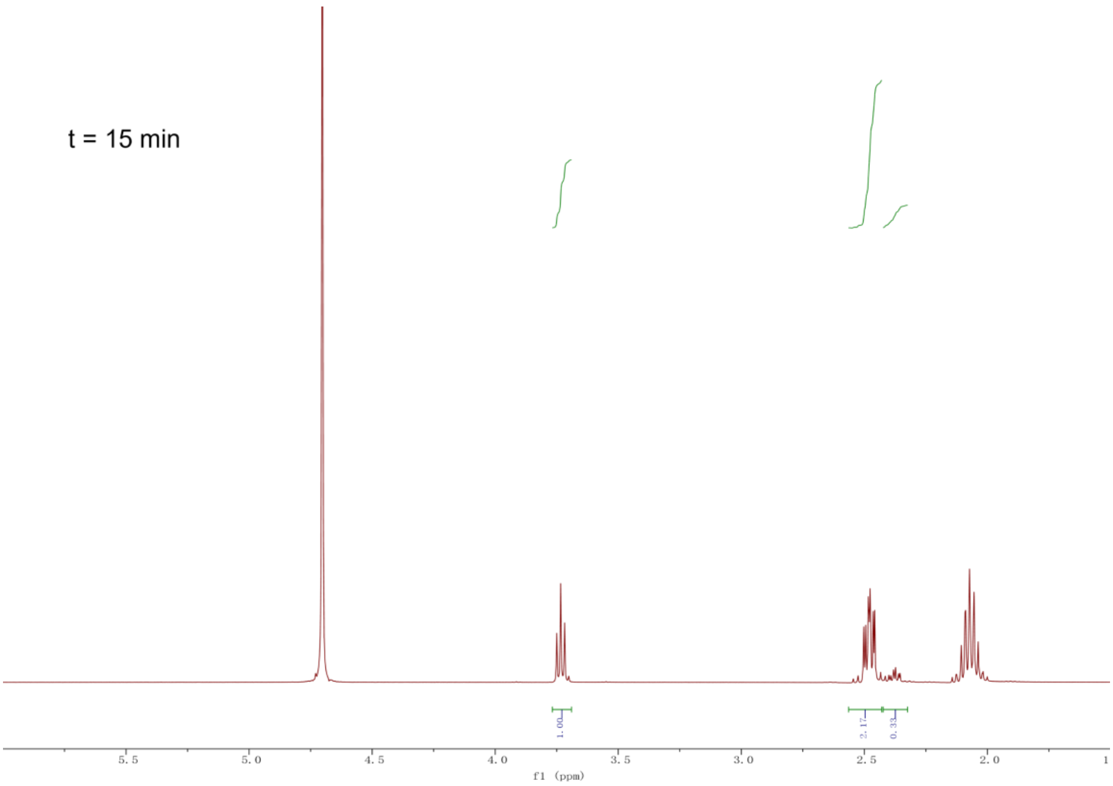

Supplementary Fig. 35 ^{1}H NMR spectrum monitoring the relative levels of Glu and Gln after reacting 15 min in the absence of ATP.

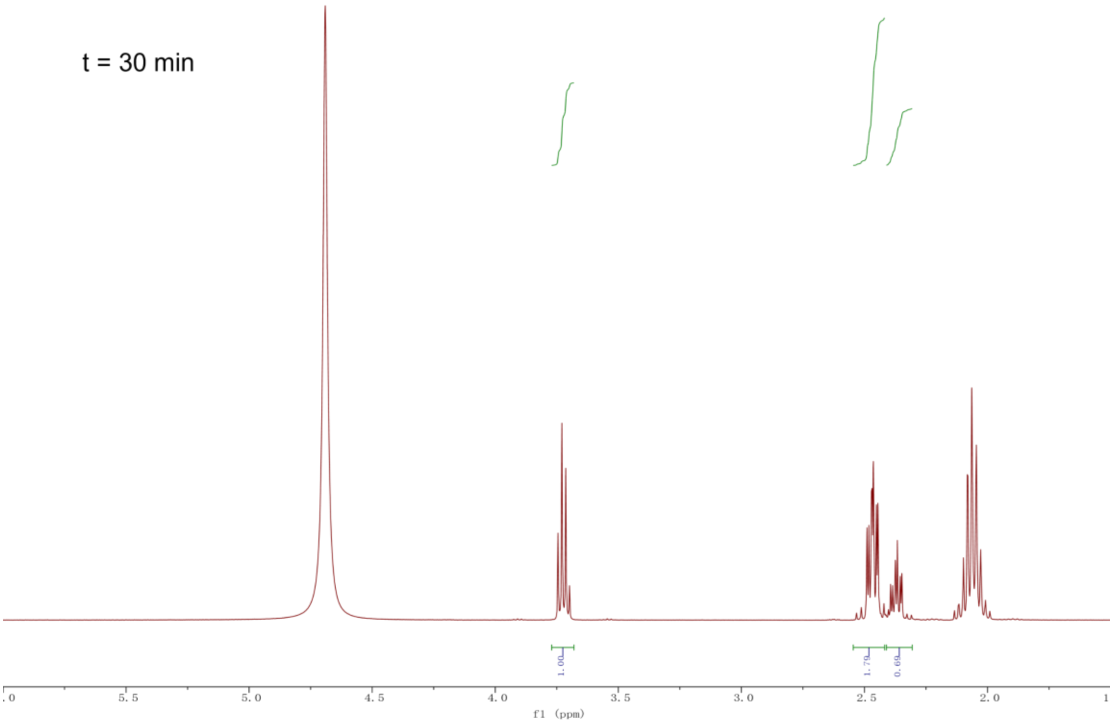

Supplementary Fig. 36 ^{1}H NMR spectrum monitoring the relative levels of Glu and Gln after reacting 30 min in the absence of ATP.

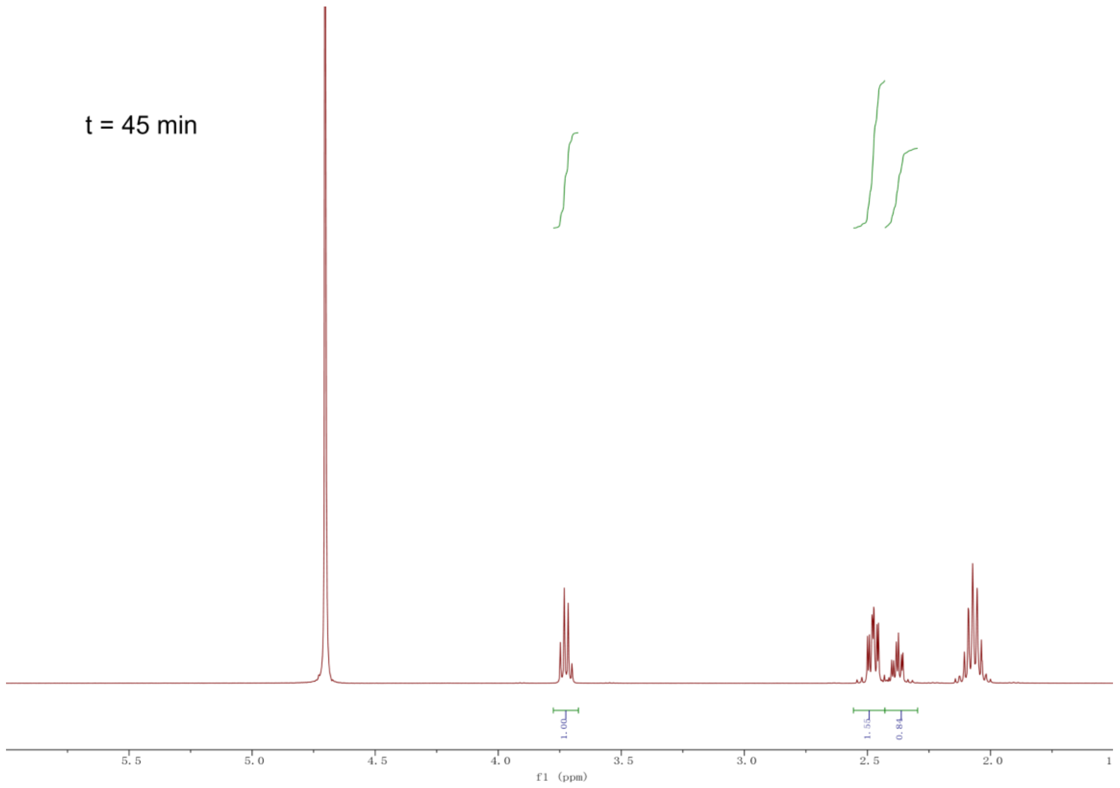

Supplementary Fig. 37 ^{1}H NMR spectrum monitoring the relative levels of Glu and Gln after reacting 45 min in the absence of ATP.

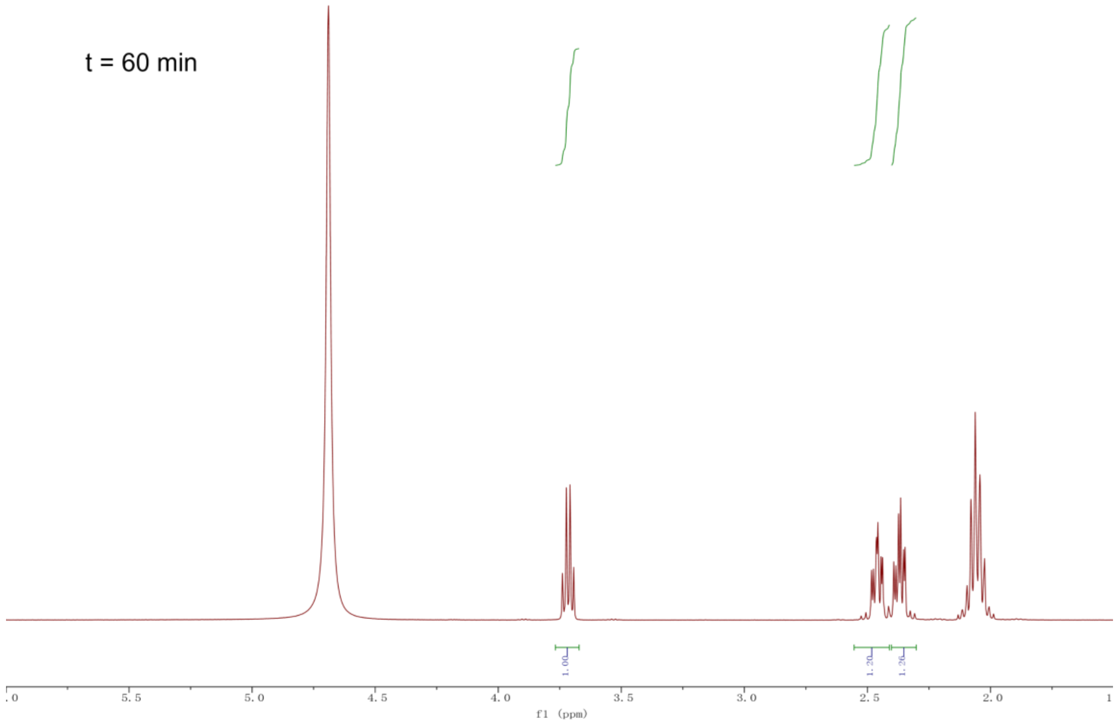

Supplementary Fig. 38 ^{1}H NMR spectrum monitoring the relative levels of Glu and Gln after reacting 60 min in the absence of ATP.

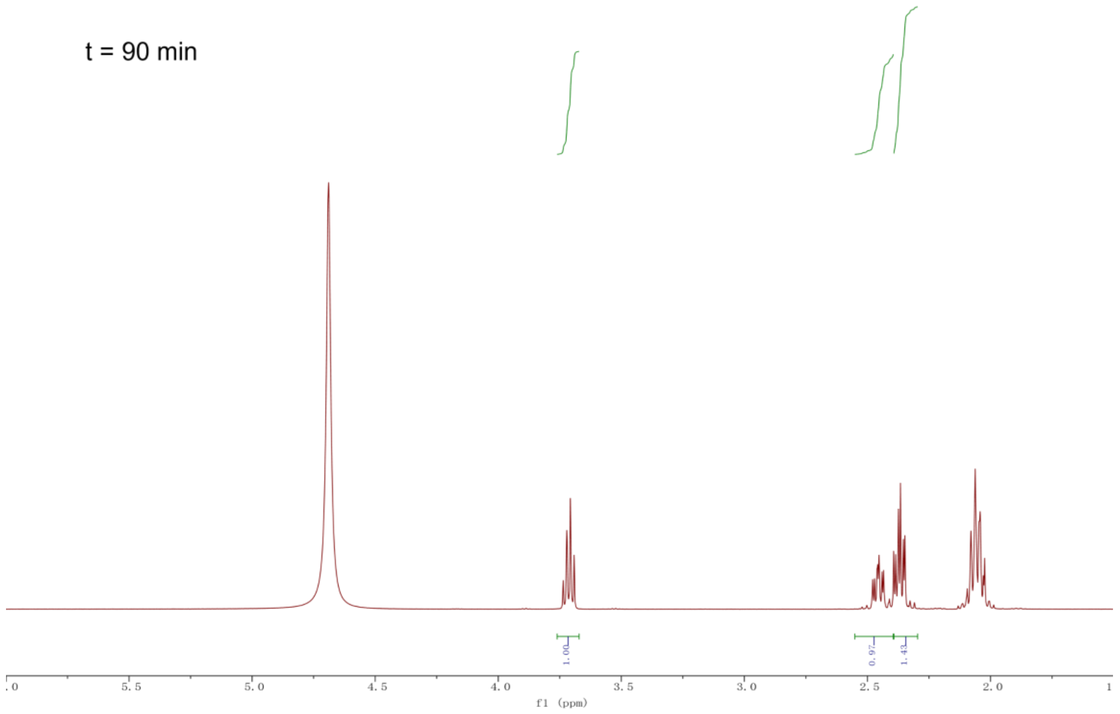

Supplementary Fig. 39 ^{1}H NMR study monitoring the relative levels of Glu and Gln after reacting 90 min in the absence of ATP.

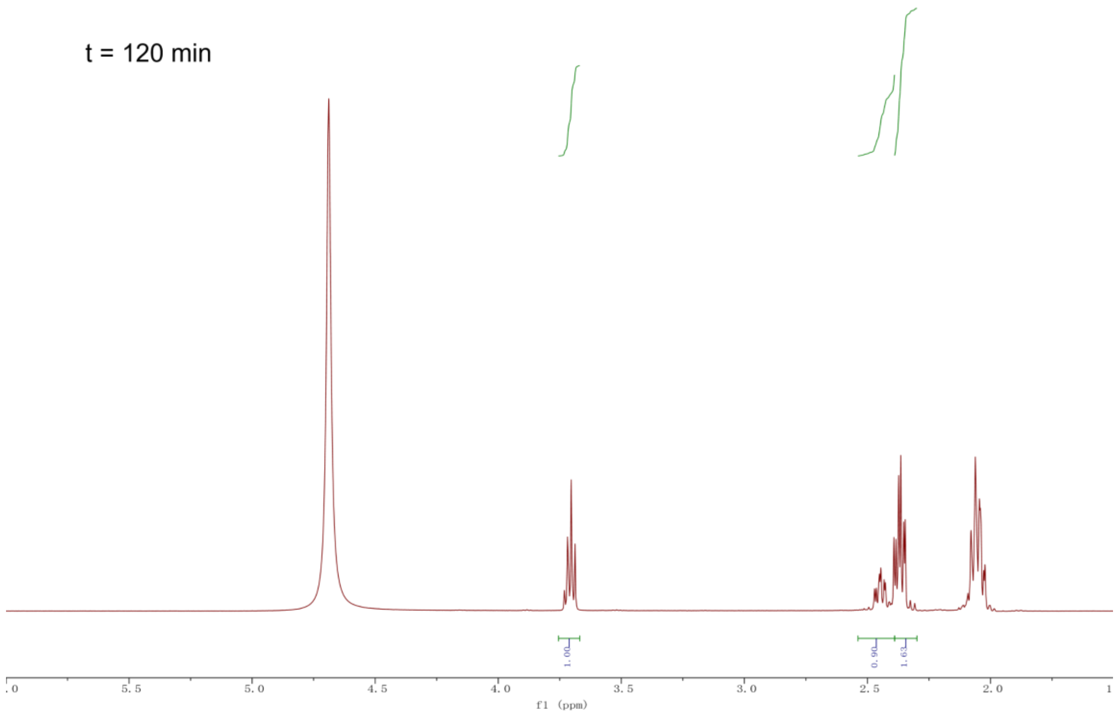

Supplementary Fig. 40 ^{1}H NMR spectrum monitoring the relative levels of Glu and Gln after reacting 120 min in the absence of ATP.

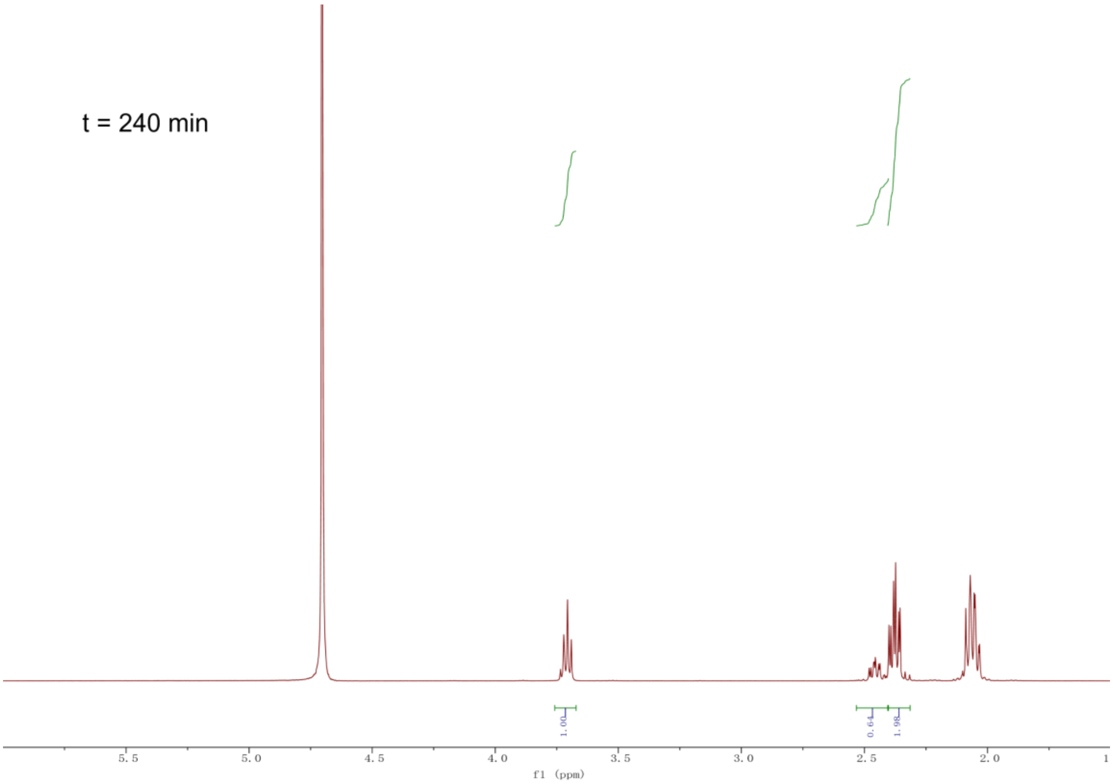

Supplementary Fig. 41 ^{1}H NMR spectrum monitoring the relative levels of Glu and Gln after reacting 240 min in the absence of ATP.

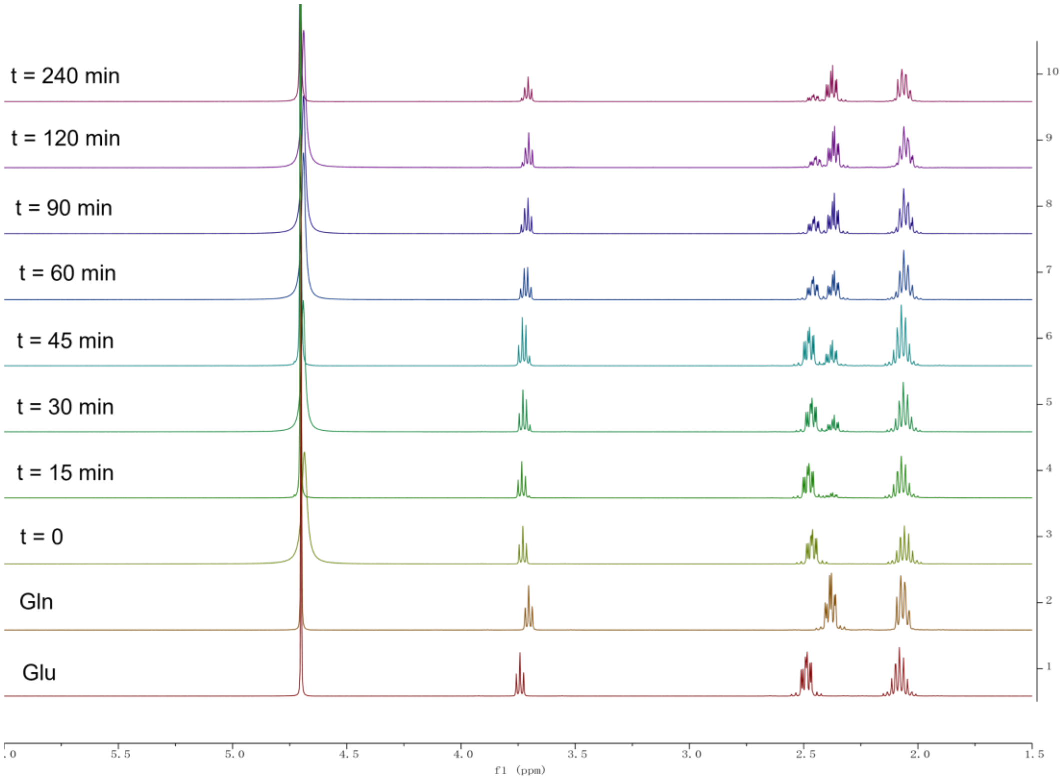

Supplementary Fig. 42 Merged ^{1}H NMR spectra from Supplementary Fig. 34 to Supplementary Fig. 41.

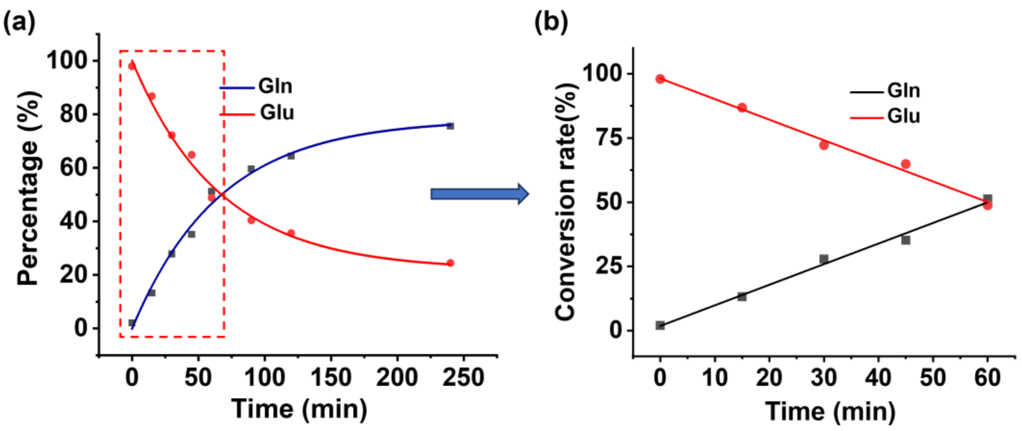

Supplementary Fig. 43 The results of NMR integrations from Supplementary Fig. 34 - Supplementary Fig. 41. The conversion rates of Glu to Gln in the catalytic system (a) and a linear relation in the 0-60 min (b).

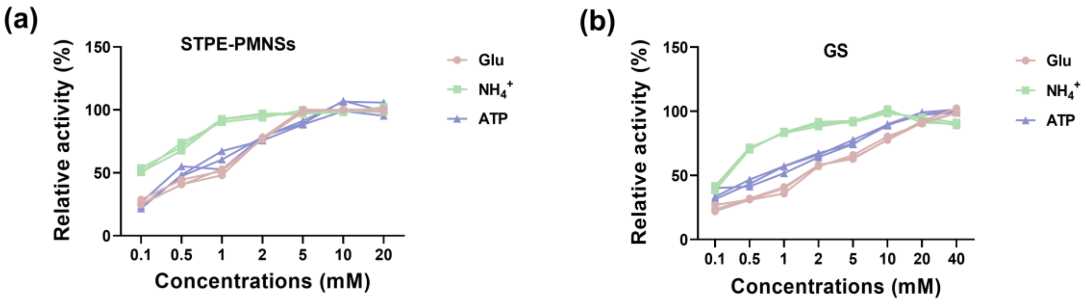

Supplementary Fig. 44 The impacts of Glu, NH_4^+, and ATP concentration on the GS activities of STPE-PMNSs (a) and natural GS (b). Glu (0.1-40 mM for natural GS, 0.1-20 mM for STPE-PMNSs), NH_4^+ (0.1-40 mM for natural GS, 0.1-20 mM for STPE-PMNSs), ATP (0.1-40 mM for natural GS, 0.1-20 mM for STPE-PMNSs) were reacted in 1 mL reaction system (pH = 7.0) under the conditions of 100 μg mL^{-1} STPE-PMNSs or natural GS. Data points presented in this figure represent three (n=3) independent experiments.

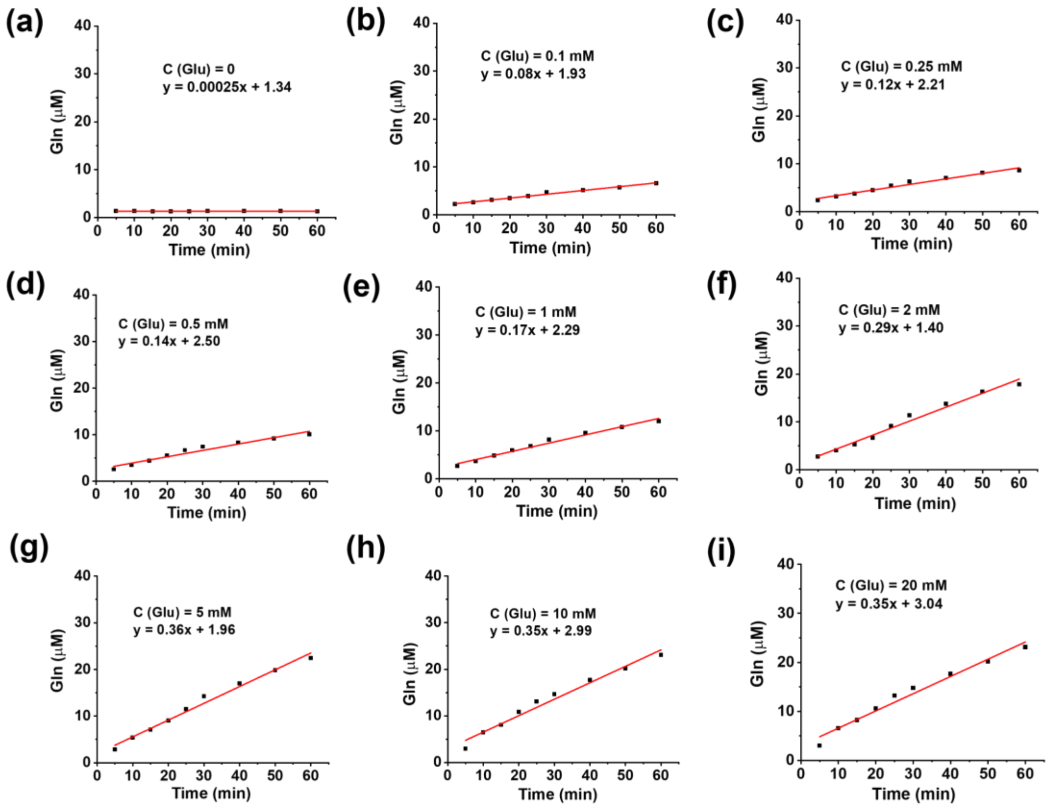

Supplementary Fig. 45 The reaction rate of STPE-PMNSs in different Glu concentrations.

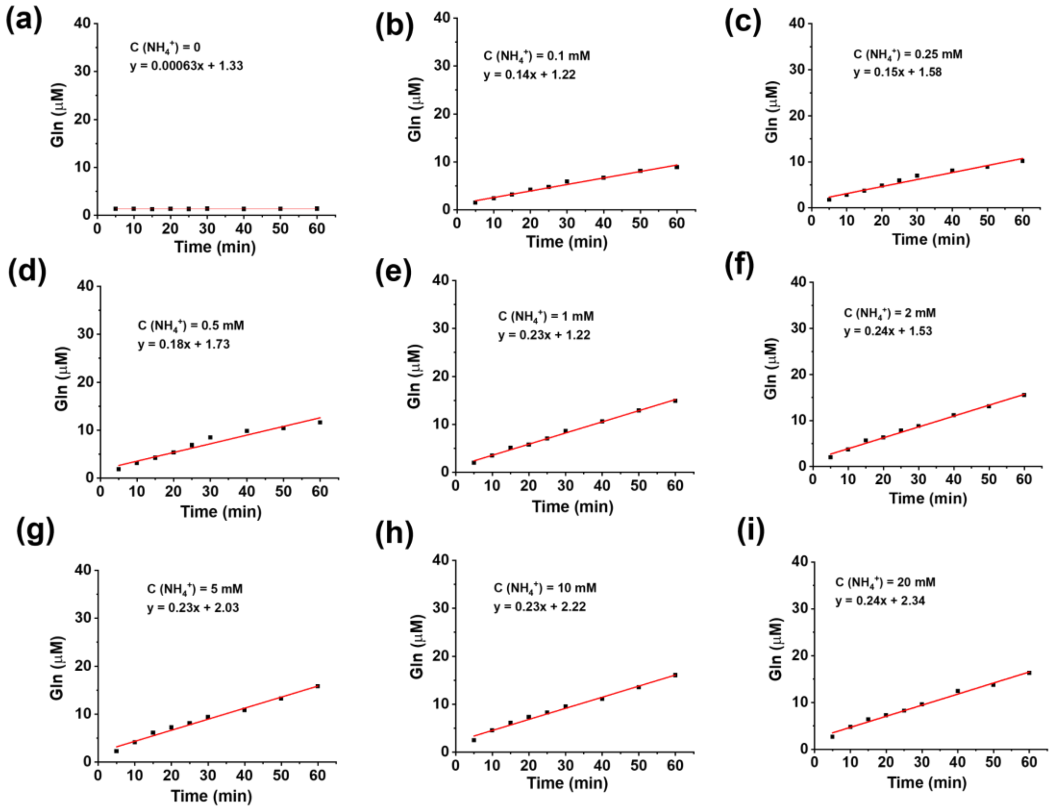

Supplementary Fig. 46 The reaction rate of STPE-PMNSs in different NH_4^+ concentrations.

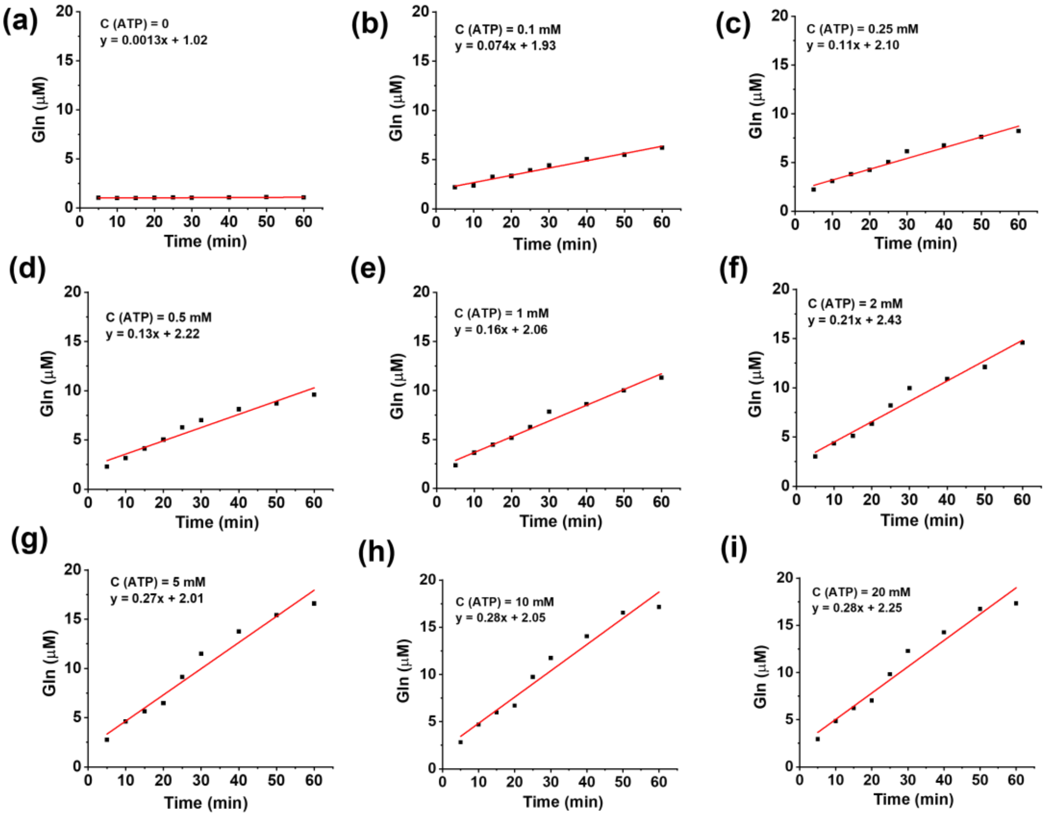

Supplementary Fig. 47 The reaction rate of STPE-PMNSs in different ATP concentrations.

Supplementary Table 1. Summary of reaction rate of STPE-PMNSs in different Glu, NH_4^+ and ATP concentrations.

Glu (mM)	Reaction rate (nmol/min/mg)	NH_4^+ (mM)	Reaction rate (nmol/min/mg)	ATP (mM)	Reaction rate (nmol/min/mg)
0	2.5×10^{-3}	0	6.3×10^{-3}	0	1.3×10^{-3}
0.1	0.8	0.1	1.4	0.1	0.7
2.5×10^{-1}	1.2	2.5×10^{-1}	1.5	2.5×10^{-1}	1.1
0.5	1.4	0.5	1.8	0.5	1.3
1.0	1.7	1.0	2.3	1.0	1.6
2.0	2.9	2.0	2.4	2.0	2.1
5.0	3.6	5.0	2.3	5.0	2.7
10.0	3.5	10.0	2.3	10.0	2.8
20.0	3.5	20.0	2.4	20.0	2.8

The unit of reaction rate is nmol/min per mg of STPE-PMNSs.

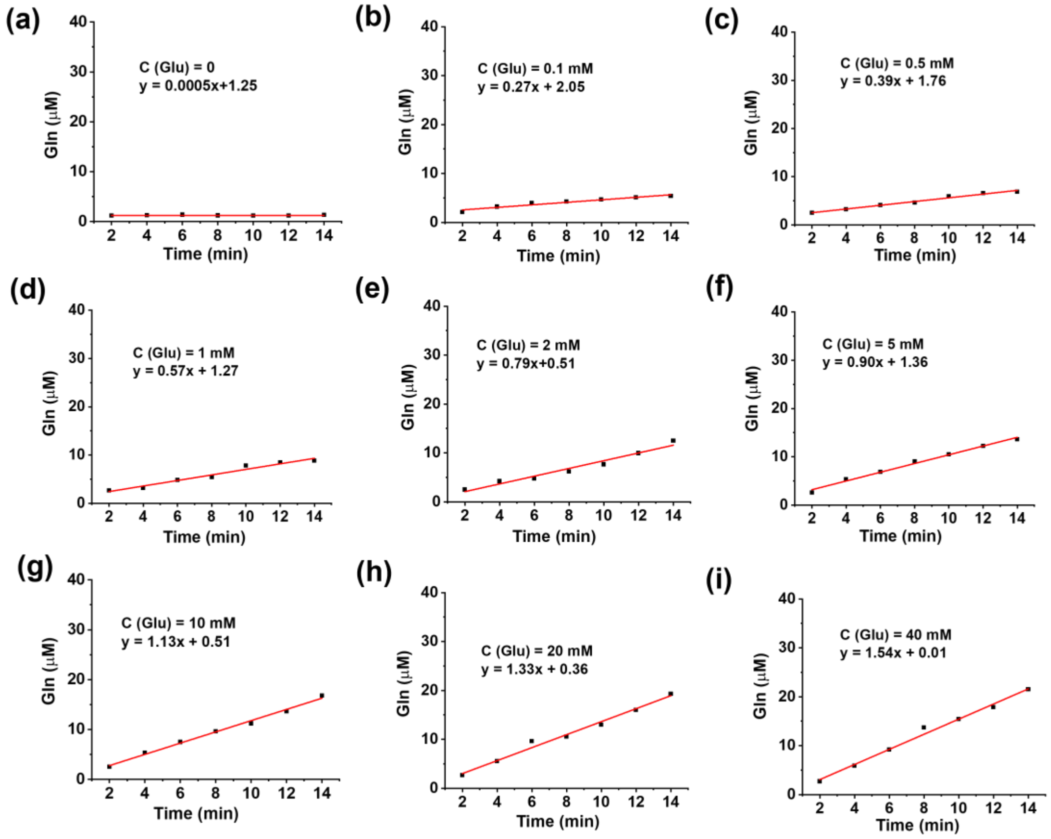

Supplementary Fig. 48 The reaction rate of natural GS in different Glu concentrations.

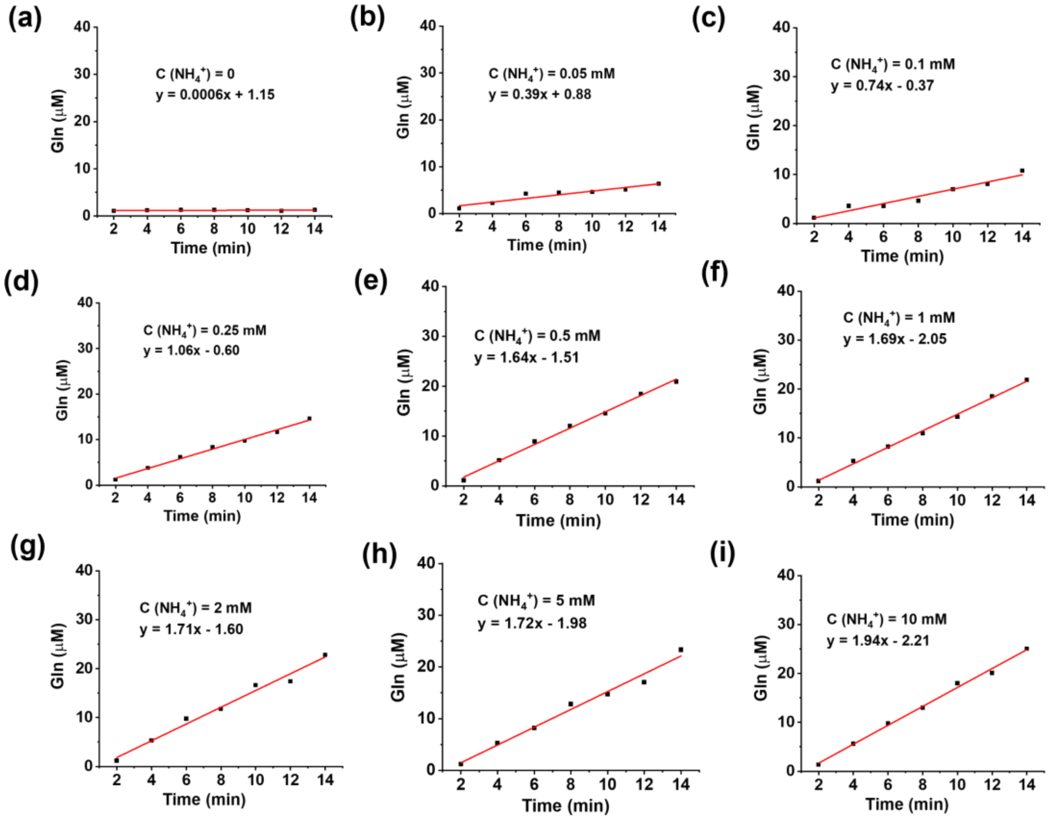

Supplementary Fig. 49 The reaction rate of natural GS in different NH_4^+ concentrations.

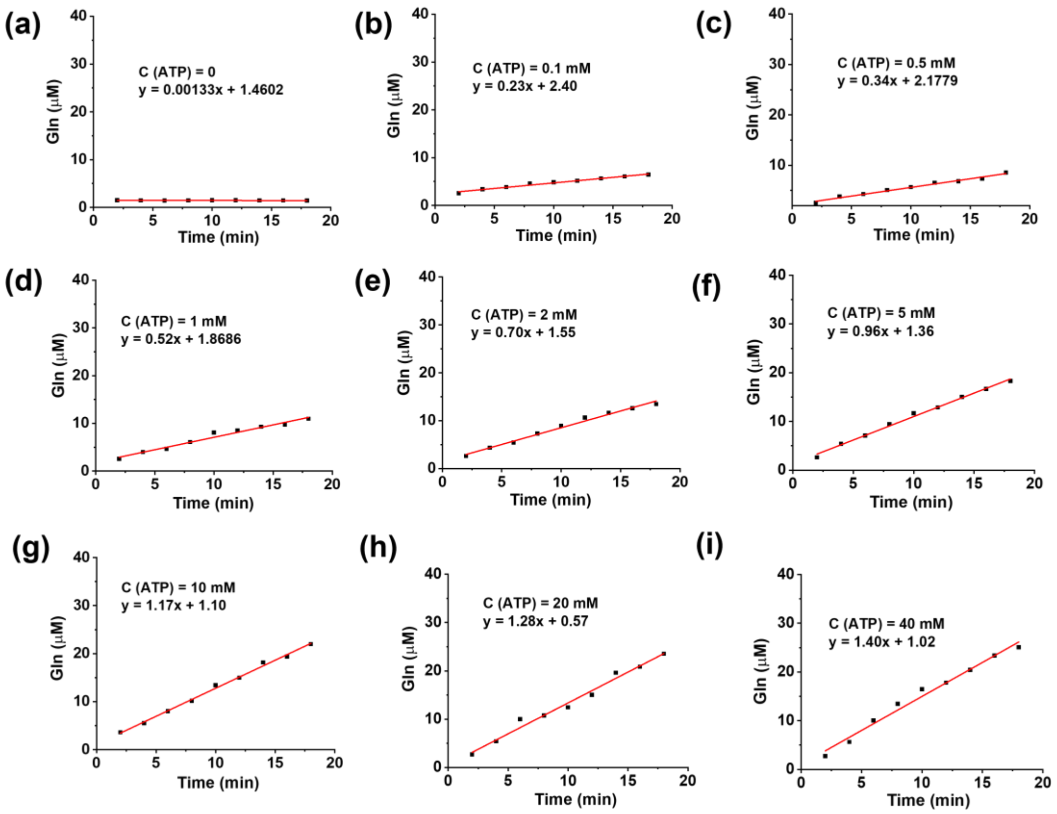

Supplementary Fig. 50 The reaction rate of natural GS in different ATP concentrations.

Supplementary Table 2. Summary of reaction rate of natural GS in different Glu, NH_4^+ and ATP concentrations.

Glu (mM)	Reaction rate (nmol/min/mg)	NH_4^+ (mM)	Reaction rate (nmol/min/mg)	ATP (mM)	Reaction rate (nmol/min/mg)
0	0.5 x 10^{-2}	0	0.6 x 10^{-2}	0	1.3 x 10^{-3}
0.1	2.7	0.5 x 10^{-1}	3.9	0.1	2.3
0.5	3.9	0.1	7.4	0.5	3.4
1.0	5.7	2.5 x 10^{-1}	10.6	1.0	5.2
2.0	7.9	0.5	16.4	2.0	7.0
5.0	9.0	1.0	16.9	5.0	9.6
10.0	11.3	2.0	17.1	10.0	11.7
20.0	13.3	5.0	17.2	20.0	12.8
40.0	15.4	10.0	19.4	40.0	14.0

The unit of reaction rate is nmol/min per mg of natural GS.

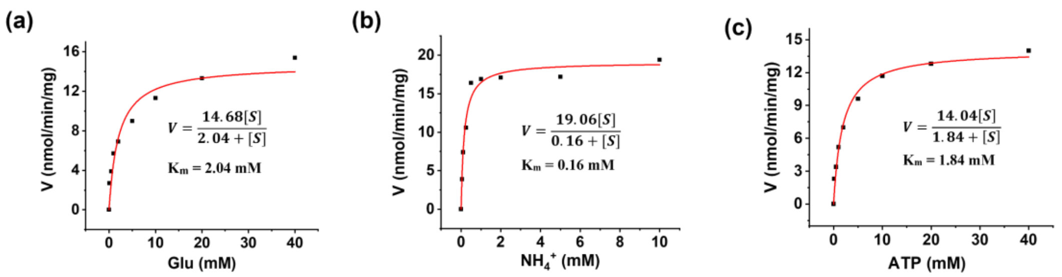

Supplementary Fig. 51 Michaelis–Menten curves of natural GS with Glu (a), NH_4^+ (b) and ATP (c) as substrate.

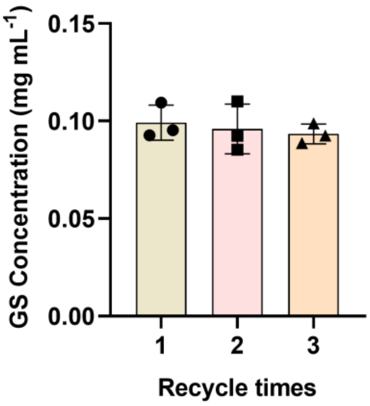

Supplementary Fig. 52 Natural GS concentration before each cycle. Data points presented in this figure represent three (n=3) independent experiments for each experimental group and are displayed as mean ± standard deviation.

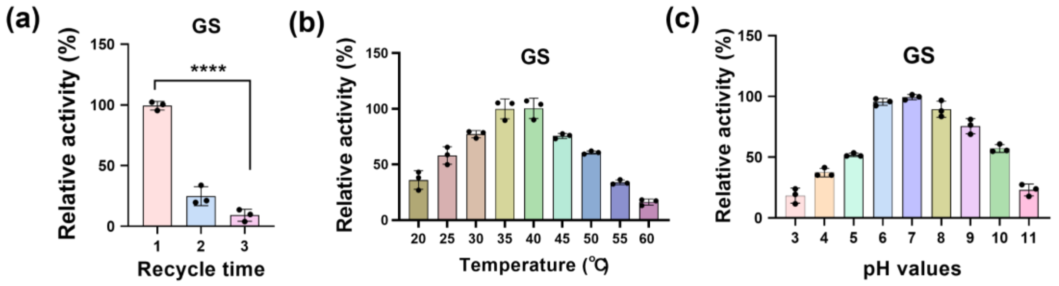

Supplementary Fig. 53 (a) Reusable capabilities of natural GS. (b) Impacts of temperature on the catalytic activity of natural GS. (c) Impacts of pH on the catalytic activity of natural GS. Data points presented in this figure represent three (n=3) independent experiments for each experimental group and are displayed as mean ± standard deviation. *p* values of (a) were determined by a two-sided *t-test*. $^{****}p<0.0001$.

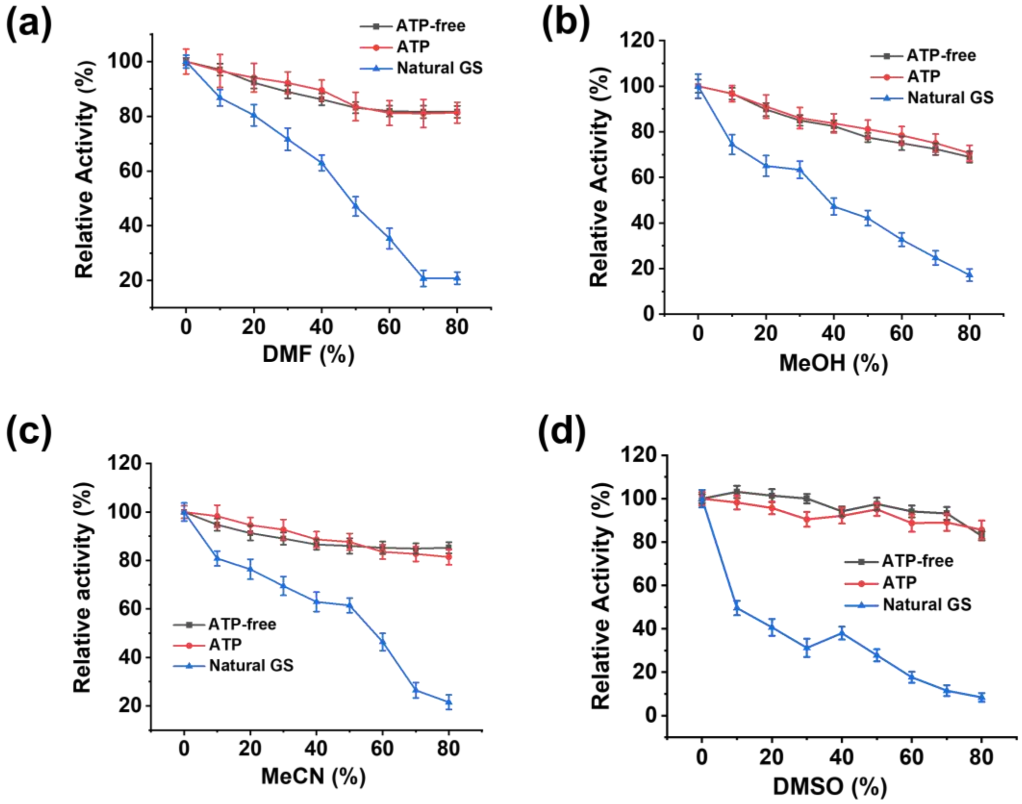

Supplementary Fig. 54 Impacts of organic solvents DMF (a), MeOH (b), MeCN (c), and DMSO (d) on the catalytic activity of STPE-PMNSs and natural GS. Data presented in this figure represent three (n=3) independent experiments for each experimental group and are displayed as mean ± standard deviation.

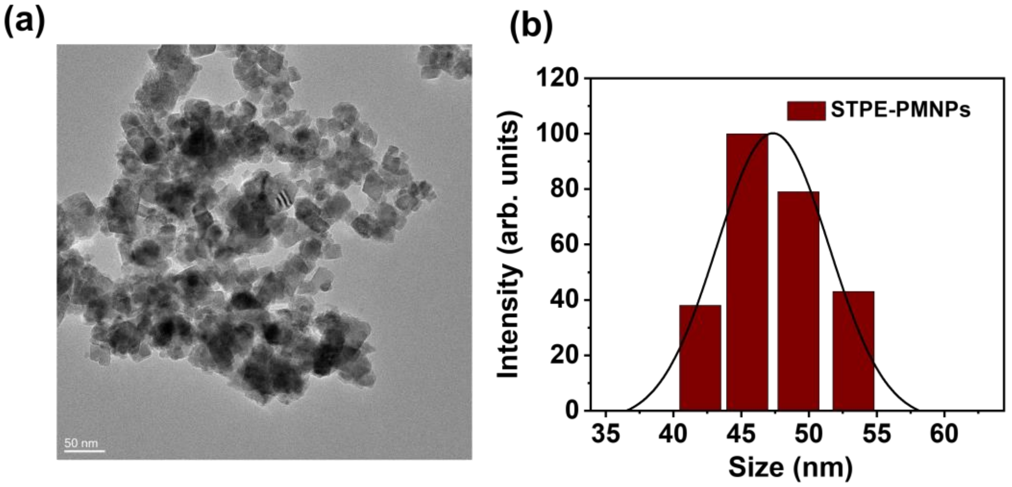

Supplementary Fig. 55 (a) The TEM image of STPE-PMNPs. (b) DLS analysis of STPE-PMNPs. Experiments were repeated three times with similar results.

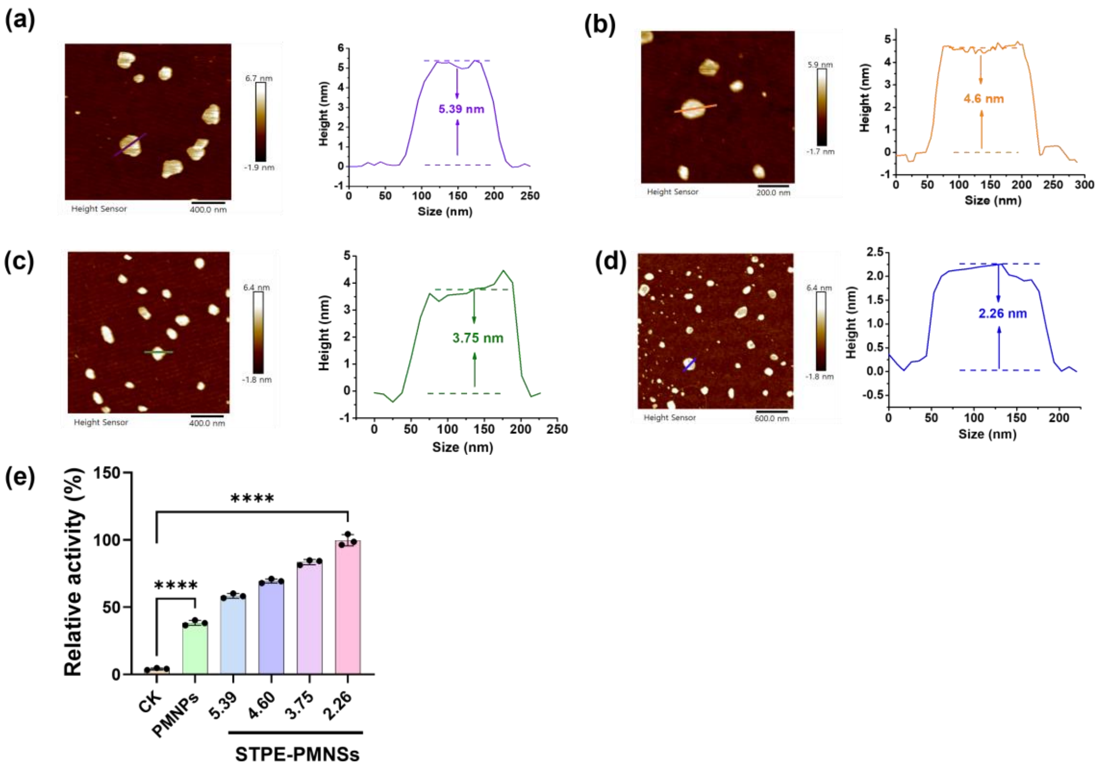

Supplementary Fig. 56 (a-d) AFM images and corresponding height profiles of nanosheets with different thicknesses were obtained by gradient centrifugation. (e) Relative GS-like activity of STPE-PMNSs with different thicknesses corresponding (a-d). Data points presented in this figure represent three (n=3) independent experiments for each experimental group and are displayed as mean ± standard deviation. *p* values of (e) were determined by a two-sided *t-test*. $^{****}p$ <0.0001. Experiments of a-d were repeated three times with similar results.

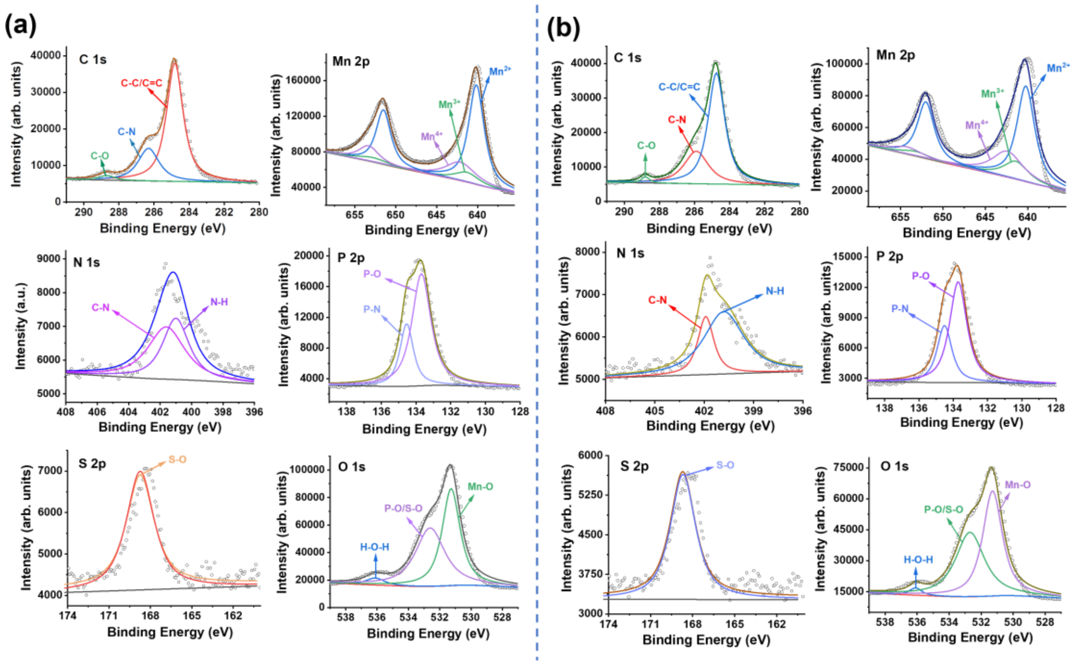

Supplementary Fig. 57 XPS analysis of post-reaction STPE-PMNSs after reaction 2 h in the presence (20 mM) (a) or absence (b) of ATP. Experiments were repeated three times with similar results.

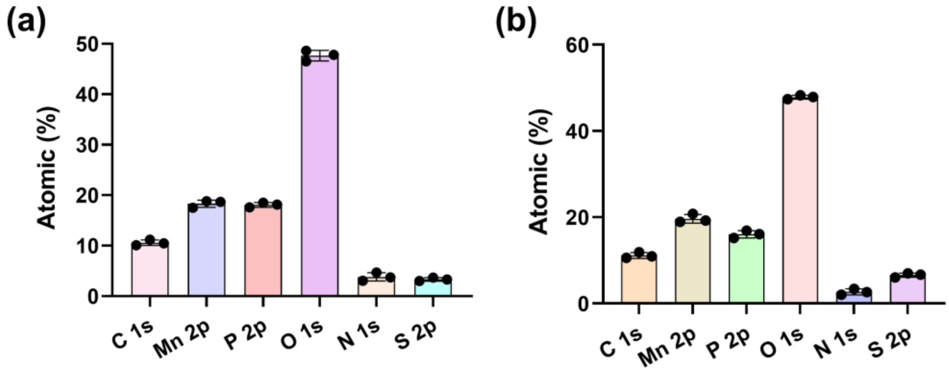

Supplementary Fig. 58 The atomic percentage from XPS of STPE-PMNSs in the presence (a) or absence (b) of ATP. Data points presented in this figure represent three (n=3) independent experiments for each experimental group and are displayed as mean ± standard deviation.

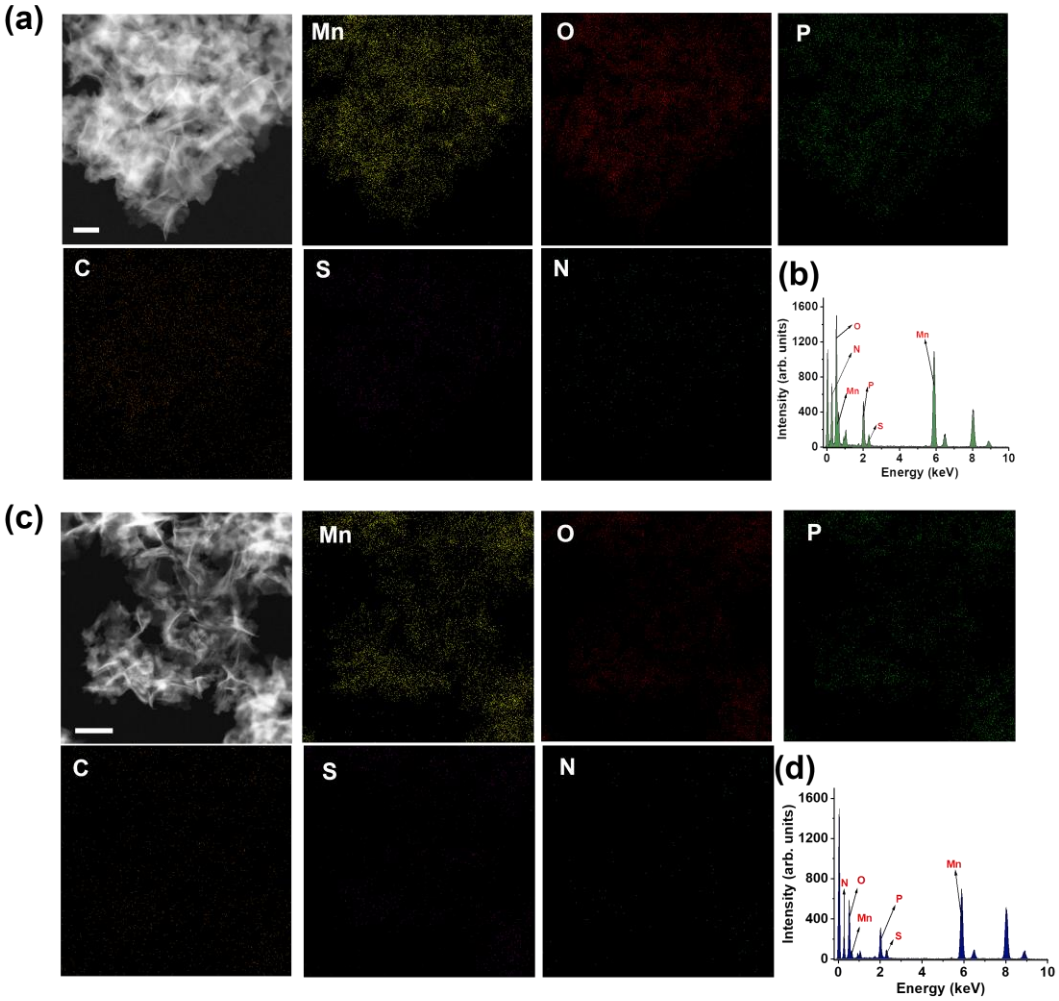

Supplementary Fig. 59 (a) STEM images and elemental mapping of STPE-PMNSs after reaction for 2 h in the presence of ATP (20 mM). Scale bar: 30 nm. (b) Element analyses of STPE-PMNSs by EDS mapping corresponding to (a). (c) STEM images and elemental mapping of STPE-PMNSs after reaction for 2 h in the absence of ATP. Scale bar: 50 nm. (d) Element analyses of STPE-PMNSs by EDS mapping corresponding to (c). Experiments were repeated three times with similar results.

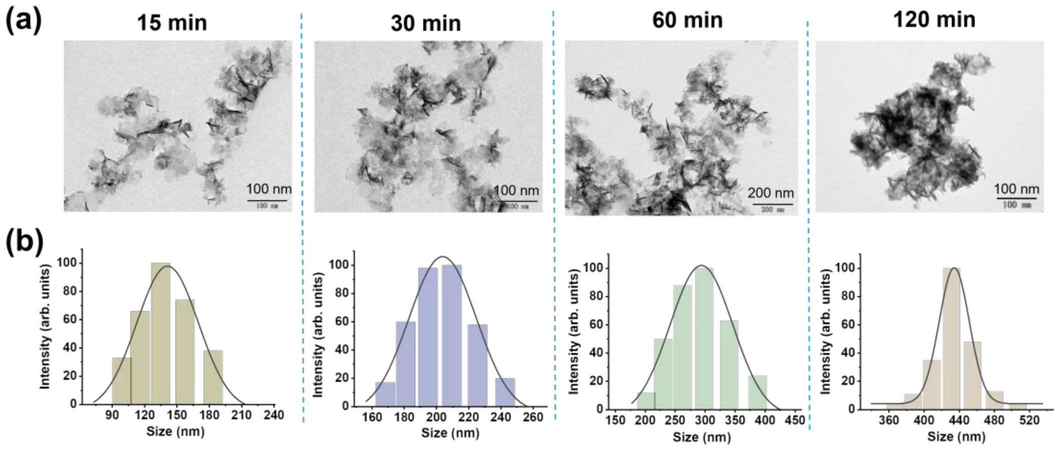

Supplementary Fig. 60 The TEM image (a) and DLS analysis (b) of STPE-PMNSs after reaction at different time points. STPE-PMNSs (100 μg mL^{-1}) promoted the conversion of Glu to Gln in 5 mL HEPES buffer (100 mM, pH 7.3) without ATP. After different reaction times, nanosheets were collected by centrifugation at 4000 g for 10 min and characterized using TEM and DLS. Experiments were repeated three times with similar results.

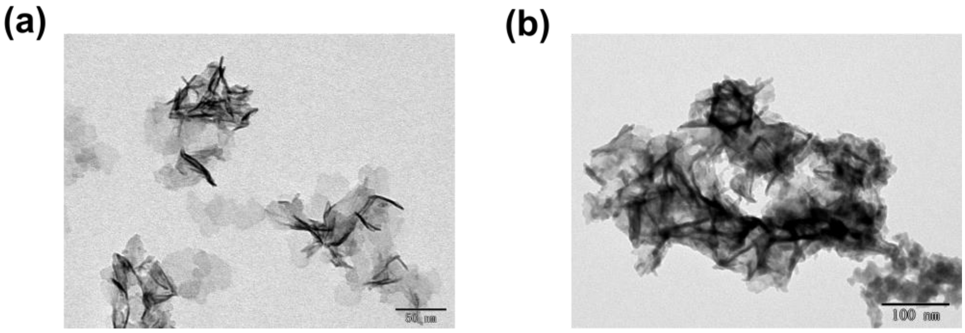

Supplementary Fig. 61 The TEM images of STPE-PMNSs before (a) and after (b) 2 h reaction in the absence of ATP. STPE-PMNSs (100 μg mL^{-1}) promoted the conversion of Glu to Gln in 5 mL HEPES buffer (100 mM, pH 7.3). After reaction 2 h, nanosheets were collected by centrifugation at 4000 g for 10 minutes and subsequently characterized using TEM. Experiments were repeated three times with similar results.

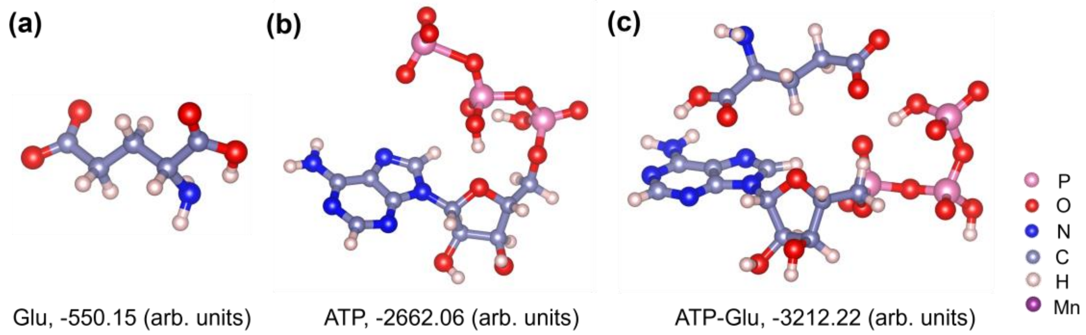

Supplementary Fig. 62 The optimized configurations and energies of ATP (a), Glu (b), and ATP-Glu (c) in the absence of STPE-PMNSs.

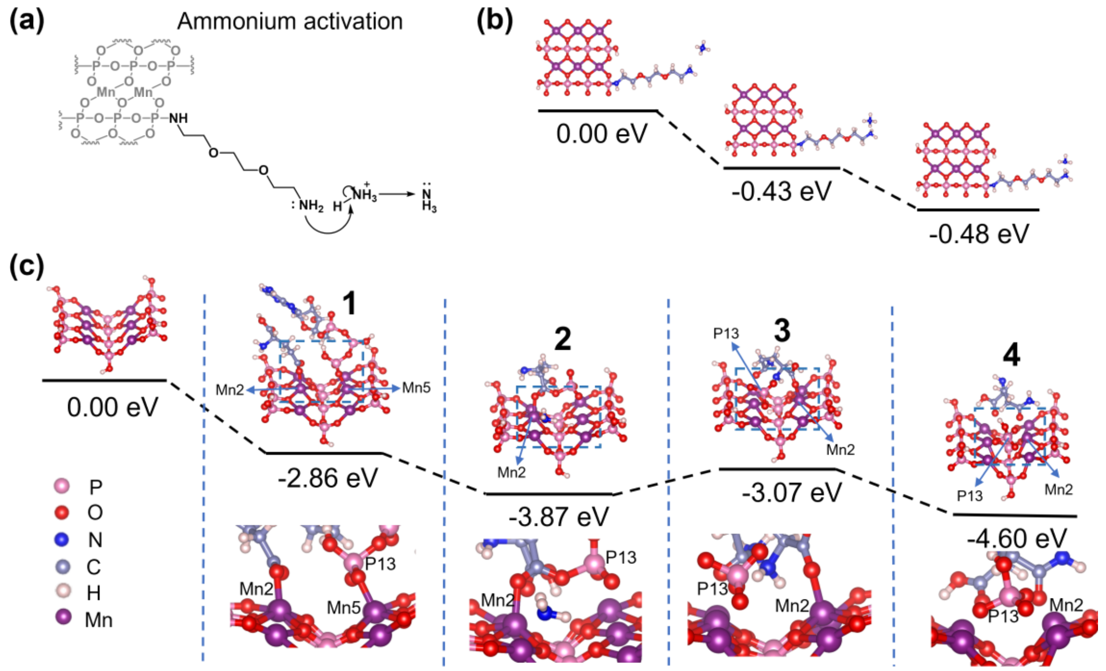

Supplementary Fig. 63 The activation of NH_4^+ (a) and the energy profile for the NH_4^+ activation in the presence of ATP (b). (c) The energy profile for STPE-PMNSs catalyzed the conversion of Glu to Gln in the presence of ATP.

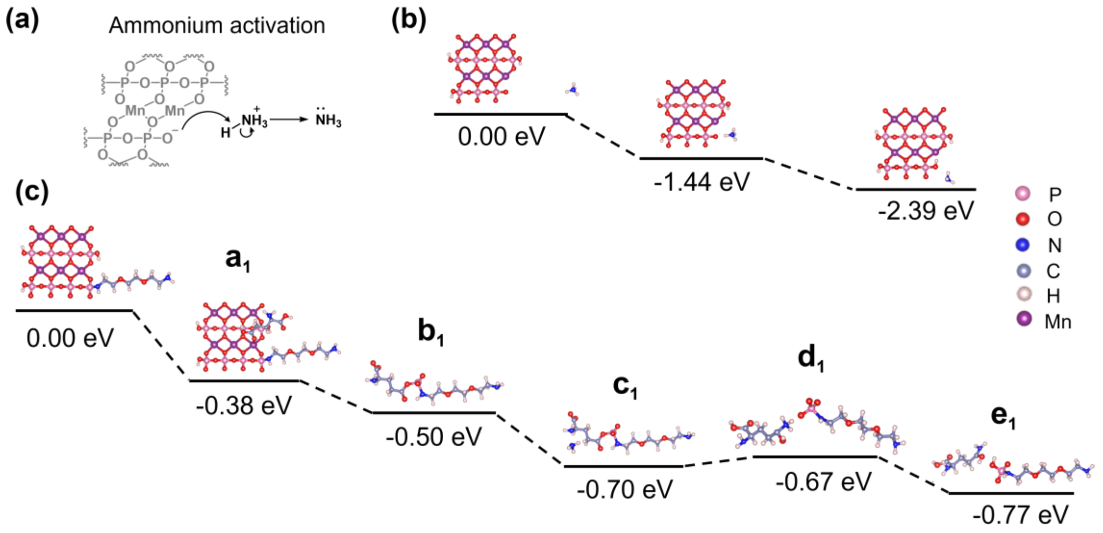

Supplementary Fig. 64 The activation of NH_4^+ (a) and the energy profile for the NH_4^+ activation (b) in the absence of ATP. (c) The energy profile for STPE-PMNSs promoted the conversion of Glu to Gln in the absence of ATP.

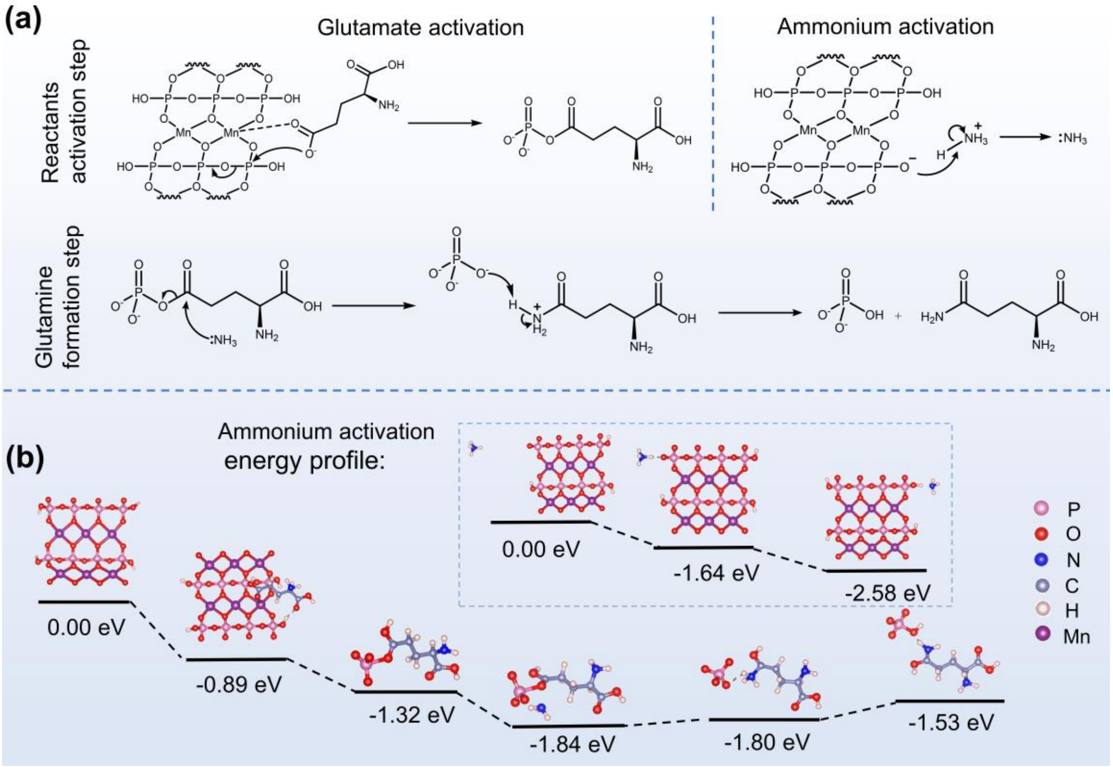

Supplementary Fig. 65 Reaction mechanism and energies of phosphate group exposed STPE-PMNSs catalyzing the conversion of Glu to Gln. (a) Glu (left) and NH_4^+ (right) activation and Gln formation step (down). (b) The energy profile for

STPE-PMNSs promoted the conversion of Glu to Gln. The insert is an energy profile for the activation of NH_4^+.

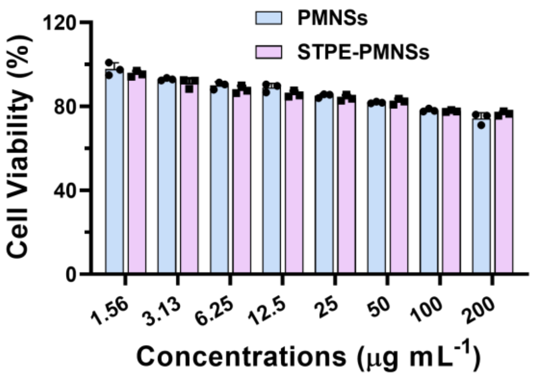

Supplementary Fig. 66 The Cell viability of SH-SY5Y cells treated with STPE-PMNSs and PMNSs for 24 h. Data points presented in this figure represent three (n=3) independent experiments for each experimental group and are displayed as mean ± standard deviation.

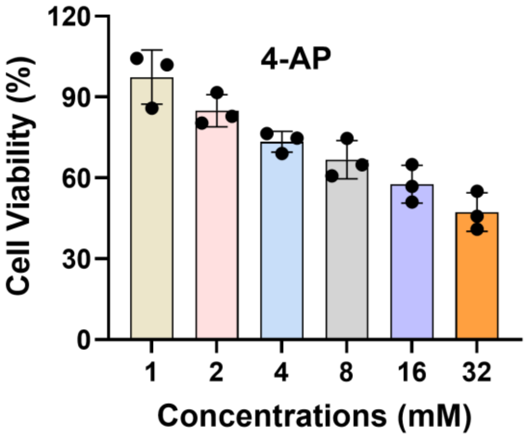

Supplementary Fig. 67 Cell viability of SH-SY5Y cells treated with 1, 2, 4, 8, 16, and 32 mM 4-AP for 24 h. Data points presented in this figure represent three (n=3) independent experiments for each experimental group and are displayed as mean ± standard deviation.

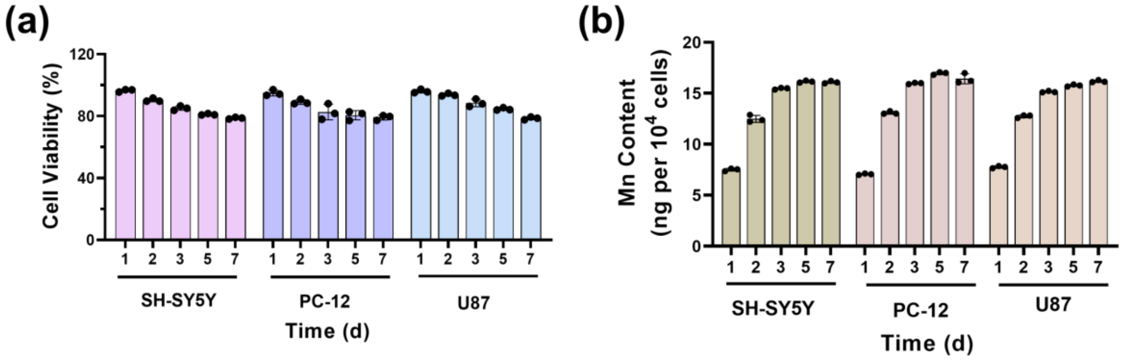

Supplementary Fig. 68 (a) Cell viability of SH-SY5Y, PC-12, U87 cells treated with 25 μg mL^{-1} STPE-PMNSs for 1, 2, 3, 5, and 7 d. (b) ICP-OES results of SH-SY5Y, PC-12, U87 cells treated with 25 μg mL^{-1} STPE-PMNSs for 1, 2, 3, 5, and 7 d. Data points presented in this figure represent three (n=3) independent experiments for each experimental group and are displayed as mean ± standard deviation.

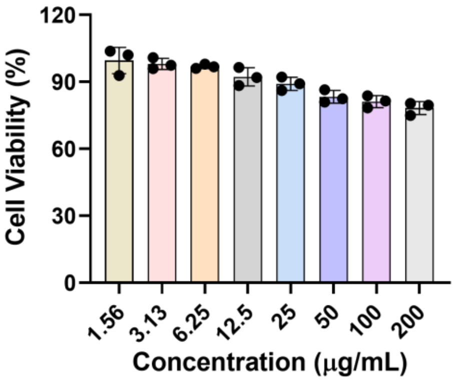

Supplementary Fig. 69 The Cell viability of SH-SY5Y cells treated with STPE-PMNSs for 24 h after 4-AP treatment 4 h. Data points presented in this figure represent three (n=3) independent experiments for each experimental group and are displayed as mean ± standard deviation.

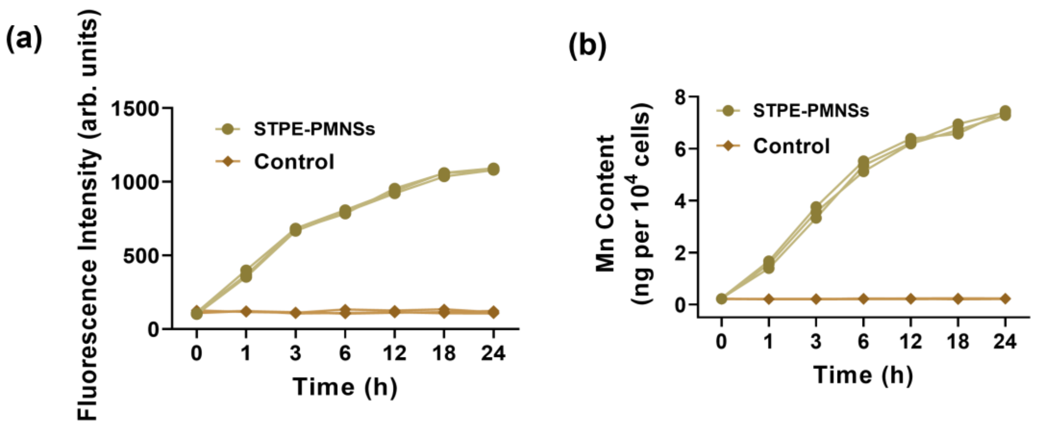

Supplementary Fig. 70 Internalization of STPE-PMNSs by cells at specific time intervals. (a) fluorescence signal changes within the cells over various periods. (b) ICP-OES results of the SH-SY5Y cells uptake of STPE-PMNSs. Data points presented in this figure represent three (n=3) independent experiments.

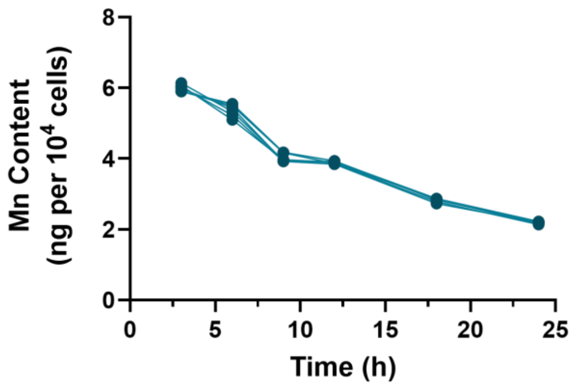

Supplementary Fig. 71 ICP-MS analysis of Mn content. SH-SY5Y cells were cultured in a 6-well plate and treated with STPE-PMNSs (25 μg mL^{-1}) for 12 h. The cells were washed with PBS three times to remove the remaining STPE-PMNSs on the cell surface. After washing, the cells were cultured for 0, 3, 6, 9, 12, and 24 h, the cells were collected and the Mn content was measured using ICP-MS. Data points presented in this figure represent three (n=6) independent experiments.

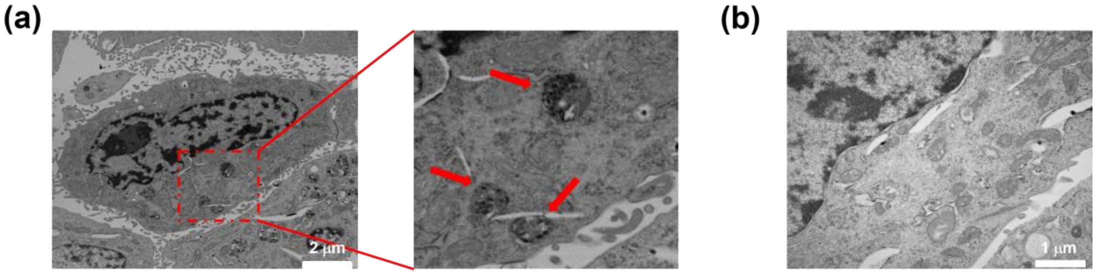

Supplementary Fig. 72 Bio-TEM images of SH-SY5Y cells treated (a) and untreated (b) with STPE-PMNSs. Experiments were repeated three times with similar results.

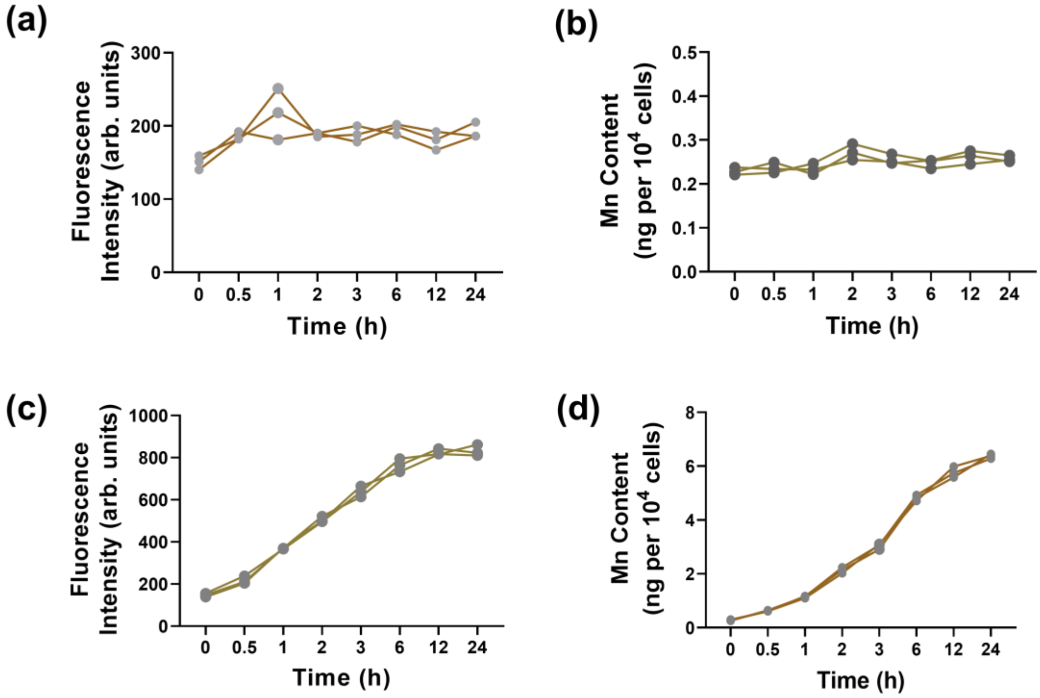

Supplementary Fig. 73 After starvation for 12 h, fluorescence intensity (a) and Mn content (b) of the cells treated with STPE-PMNSs (25 μg mL^{-1}) at different time points in a serum-free medium. After normal culture 12 h, fluorescence intensity (c) and Mn content (d) of the cells treated with STPE-PMNS (25 μg mL^{-1}) at different time points in a serum-free medium. Data points presented in this figure represent three (n=3) independent experiments.

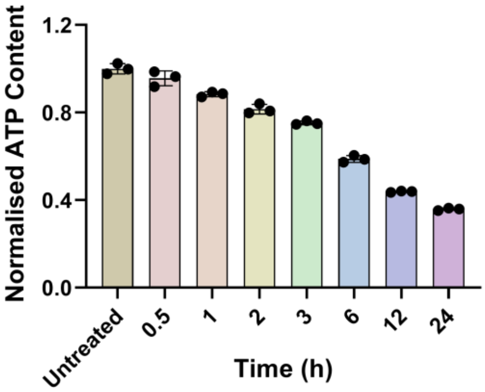

Supplementary Fig. 74 After normal culture 12 h, intracellular ATP levels of cells treated with STPE-PMNSs (25 μg mL^{-1}) at different time points in a serum-free medium. Data points presented in this figure represent three (n=3) independent experiments for each experimental group and are displayed as mean ± standard deviation.

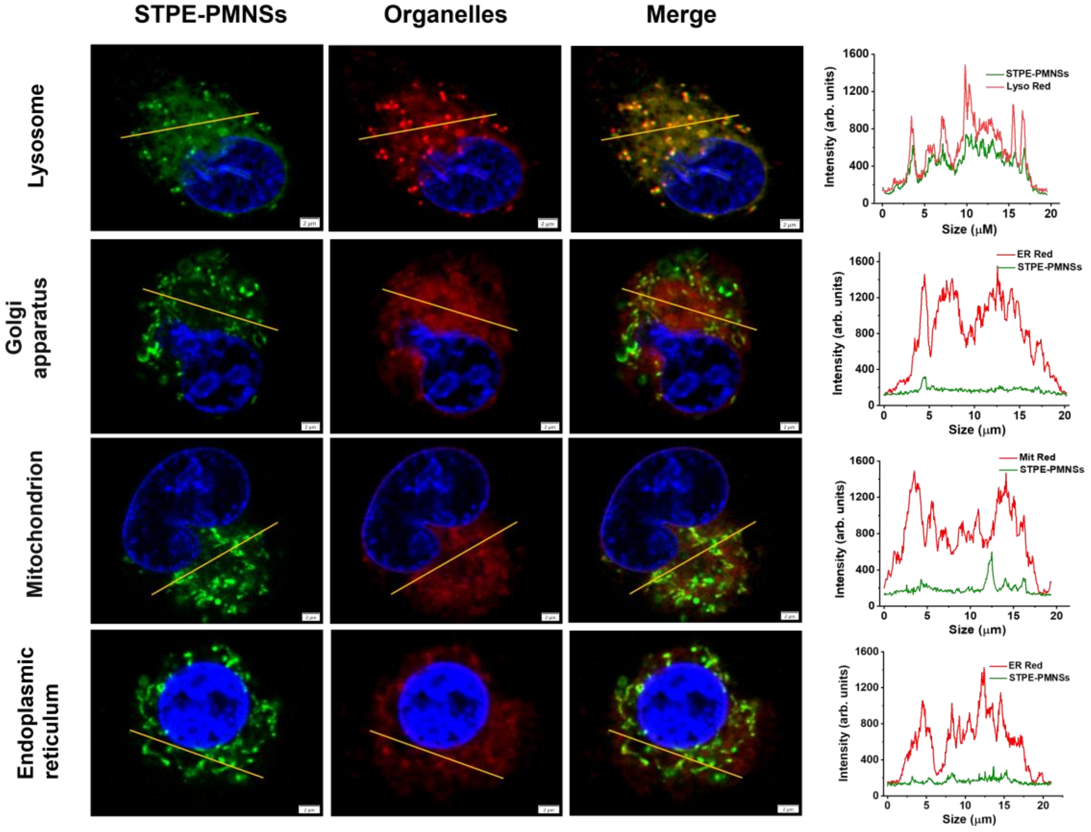

Supplementary Fig. 75 The subcellular distribution of STPE-PMNSs. Scale bar: 2 μm. Experiments were repeated three times with similar results.

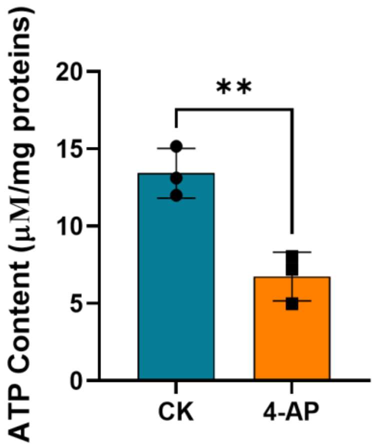

Supplementary Fig. 76 ATP content in SH-SY5Y cells after 4-AP (2 mM) was stimulated. Data points presented in this figure represent three (n=3) independent experiments for each experimental group and are displayed as mean ± standard deviation. *p* values were determined by a two-sided *t-test*. $^{****}p$ <0.0068.

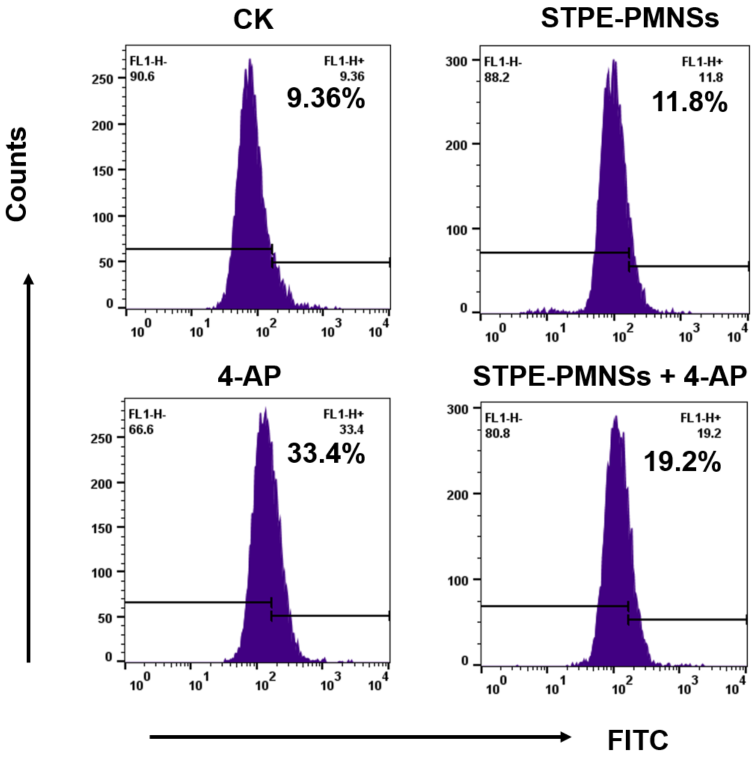

Supplementary Fig. 77 The content of intracellular Ca^{2+} in different treatment groups was analyzed by flow cytometry. Experiments were repeated three times with similar results.

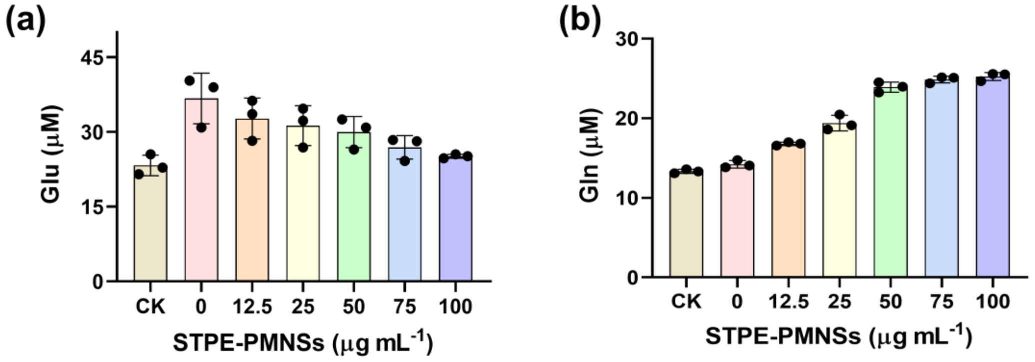

Supplementary Fig. 78 The intracellular dose-response of GS-like activity of STPE-PMNSs. Contents of Glu (a) and Gln (b) after treatment with different concentrations of STPE-PMNSs. Data points presented in this figure represent three (n=3) independent experiments for each experimental group and are displayed as mean ± standard deviation.

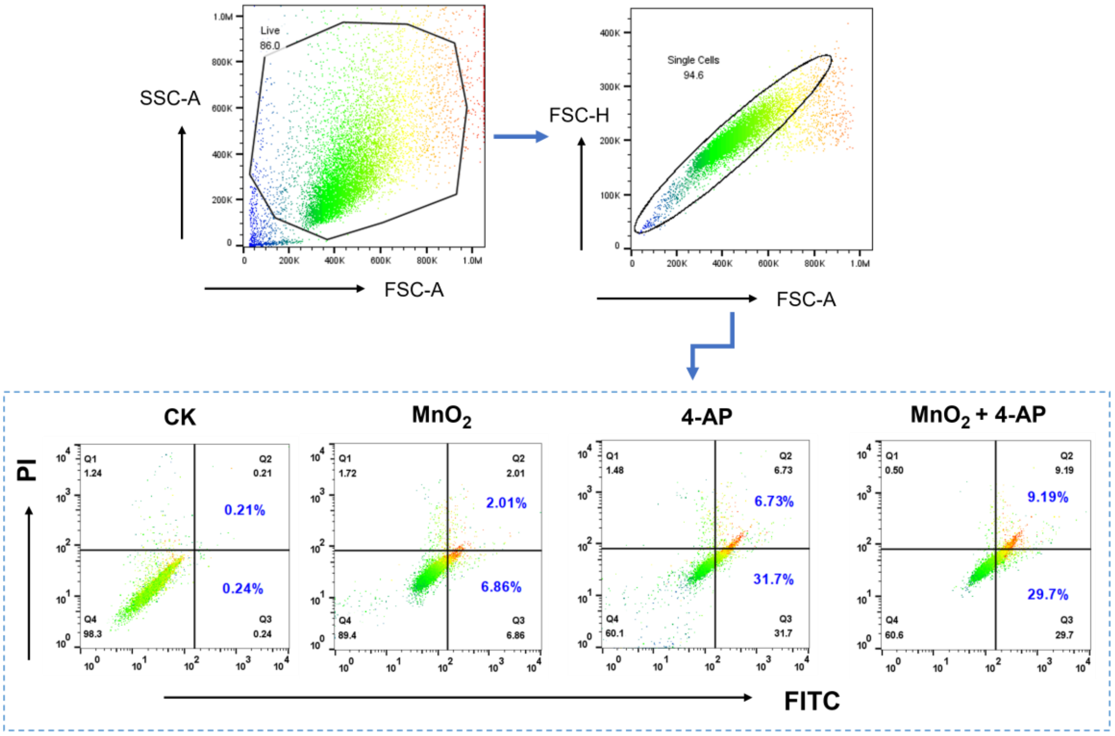

Supplementary Fig. 79 Flow cytometry analysis of the apoptosis level of SH-SY5Y cells after 24 h in different treatment groups. Experiments were repeated three times with similar results.

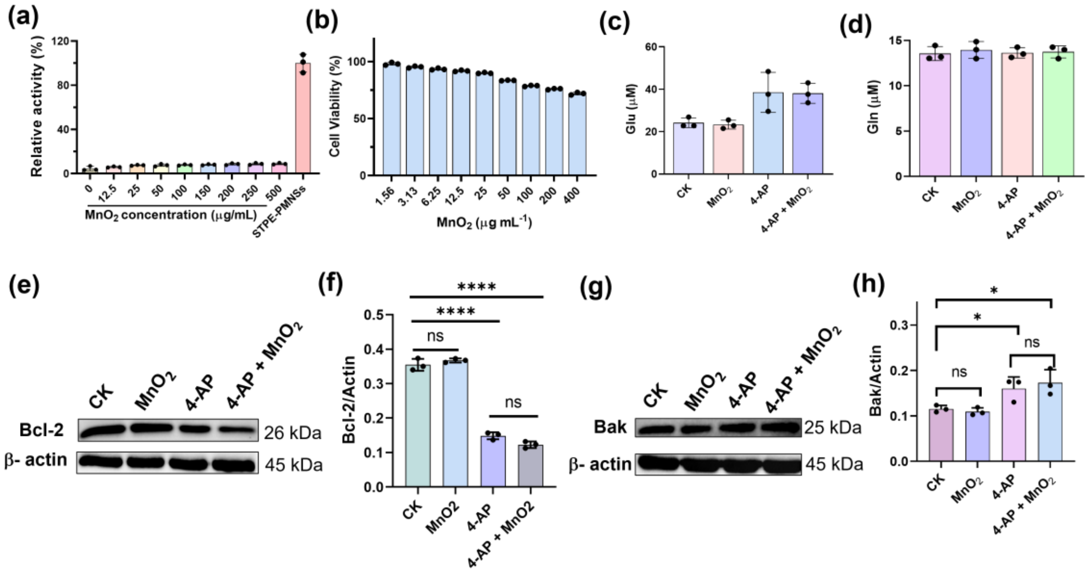

Supplementary Fig. 80 The extracellular catalytic activity study of MnO_2. (a) Intracellular GS-like activity of MnO_2. (b) Cell viability of SH-SY5Y cells treated with different concentrations of MnO_2 for 24 h. (c-d) The content of intracellular Glu and Gln in different treatment groups after 24 h. (e-f) Bcl-2 expression levels in SH-SY5Y cells after 24 h in different treatment groups. (g-h) Bax expression levels in SH-SY5Y cells after 24 h in different treatment groups. Data points presented in figures a-d, g, and i represent three (n=3) independent experiments for each experimental group and are displayed as mean ± standard deviation. $^{****}p<0.0001$, $^{*}p$ (4-AP *vs* CK) = 0.0452, $^{*}p$ (4-AP + MnO_2 *vs* CK) = 0.0280. The samples in figures e and g were derived from the same experiment and blots were processed in parallel.

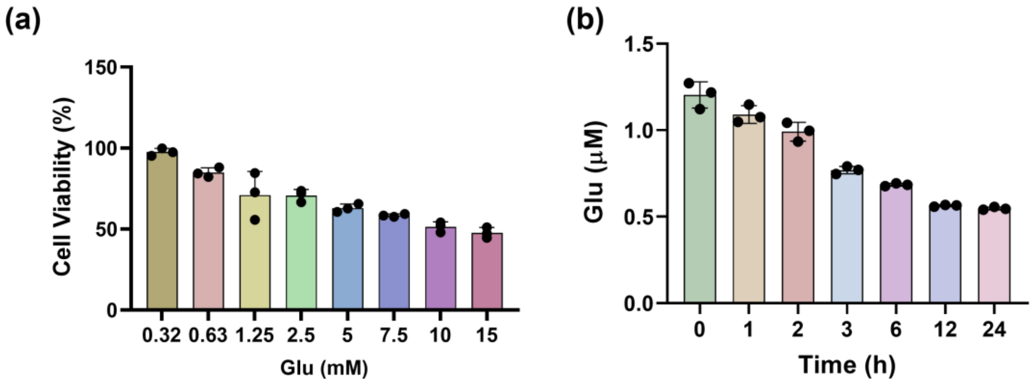

Supplementary Fig. 81 (a) Cell viability of SH-SY5Y cells treated with various concentrations of Glu for 24 h. (b) Relationship between Glu levels in the medium and incubation time of STPE-PMNSs. Data points presented in this figure represent three

(n=3) independent experiments for each experimental group and are displayed as mean ± standard deviation.

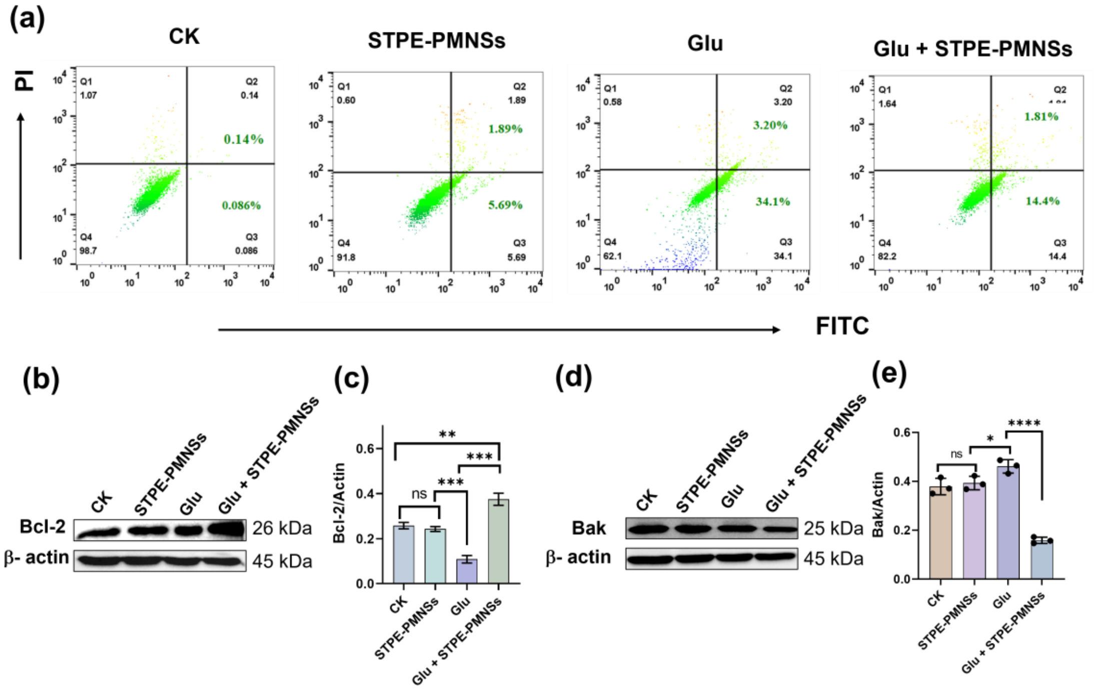

Supplementary Fig. 82 Extracellular Glu scavenging ability of STPE-PMNSs. (a) The apoptosis level of SH-SY5Y cells after 24 h in different treatment groups. (b-c) Bcl-2 expression levels in SH-SY5Y cells after 24 h in different treatment groups. $^{***}p$ (Glu *vs* CK) = 0.0003, $^{***}p$ (Glu + STPE-PMNSs *vs* Glu) = 0.0001, $^{**}p$ (Glu + STPE-PMNSs *vs* CK) = 0.00026. (d-e) Bax expression levels in SH-SY5Y cells after 24 h in different treatment groups. $^{***}p$ (Glu *vs* CK) = 0.0004, $^{****}p$ (Glu + STPE-PMNSs *vs* Glu) < 0.0001. Data points presented in figures c and e represent three (n=3) independent experiments for each experimental group and are displayed as mean ± standard deviation. *p* values of (c) and (d) were determined by a two-sided *t-test*. The samples in figures b and d were derived from the same experiment and blots were processed in parallel. Experiments were repeated three times with similar results.

Supplementary References

1. Munch, H., Hansen, J.S., Pittelkow, M., Christensen, J.B. & Boas, U. A new efficient synthesis of isothiocyanates from amines using di-tert-butyl dicarbonate. *Tetrahedron Lett.* **49**, 3117-3119 (2008).
2. Ooyama, Y. *et al.* Aggregation-induced emission (AIE) characteristic of water-soluble tetraphenylethene (TPE) bearing four sulfonate salts. *New J. Chem.* **41**, 4747-4749 (2017).
3. Fernandes-Cunha, G.M. *et al.* Delivery of Inorganic Polyphosphate into Cells Using Amphipathic Oligocarbonate Transporters. *ACS Cent. Sci.* **4**, 1394-1402 (2018).
4. Kresse, G. & Furthmüller, J. Efficiency of ab-initio total energy calculations for metals and semiconductors using a plane-wave basis set. *Comp. Mater. Sci.* **6**, 15-50 (1996).
5. Kresse, G. & Furthmüller, J. Efficient iterative schemes for ab initio total-energy calculations using a plane-wave basis set. *Phys. Rev. B* **54**, 11169-11186 (1996).
6. Ernzerhof, M. & Perdew, J.P. Generalized gradient approximation to the angle- and system-averaged exchange hole. *J. Chem. Phys.* **109**, 3313-3320 (1998).
7. Grimme, S., Antony, J., Ehrlich, S. & Krieg, H. A consistent and accurate ab initio parametrization of density functional dispersion correction (DFT-D) for the 94 elements H-Pu. *J. Chem. Physi.* **132**, 154104 (2010).
8. Grimme, S., Hansen, A., Ehlert, S. & Mewes, J.-M. r2SCAN-3c: A "Swiss army knife" composite electronic-structure method. *J. Chem. Phy.* **154** (2021).
9. Neese, F. The ORCA quantum chemistry program package. *WIREs Comput Mol Sci* **2**, 73-78 (2012).
10. Barone, V. & Cossi, M. Quantum Calculation of Molecular Energies and Energy Gradients in Solution by a Conductor Solvent Model. *J. Phys. Chem. A* **102**, 1995-2001 (1998).
11. Weigend, F. Accurate Coulomb-fitting basis sets for H to Rn. *Phys. Chem. Chem. Phys.* **8**, 1057-1065 (2006).
12. Perdew, J.P., Burke, K. & Ernzerhof, M. Generalized Gradient Approximation Made Simple. *Phys. Rev. Lett.* **77**, 3865-3868 (1996).
13. Perdew, J.P., Burke, K. & Ernzerhof, M. Generalized Gradient Approximation Made Simple [Phys. Rev. Lett. 77, 3865 (1996)]. *Phys. Rev. Lett.* **78**, 1396-1396 (1997).
14. Frisch, M.J. *et al.* Gaussian 16 Rev. C.01.
15. Walkey, C.D., Olsen, J.B., Guo, H., Emili, A. & Chan, W.C.W. Nanoparticle Size and Surface Chemistry Determine Serum Protein Adsorption and Macrophage Uptake. *J. Am. Chem. Soc.* **134**, 2139-2147 (2012).
