# Supplementary material for: Biocompatible aggregation-induced emission active polyphosphate-manganese nanosheets with glutamine synthetase-like activity in excitotoxic nerve cells
Source: Nat Commun. 2024 Apr 26;15:3534. doi: 10.1038/s41467-024-47947-5 (PMC11053040; doi:10.1038/s41467-024-47947-5)
Supplement: Supplementary file 2 — Reporting Summary [file 41467_2024_47947_MOESM2_ESM.pdf]

nature portfolio

Corresponding author(s): Jing Zhao

Last updated by author(s): Mar 7, 2024

Reporting Summary

Nature Portfolio wishes to improve the reproducibility of the work that we publish. This form provides structure for consistency and transparency in reporting. For further information on Nature Portfolio policies, see our Editorial Policies and the Editorial Policy Checklist.

Statistics

For all statistical analyses, confirm that the following items are present in the figure legend, table legend, main text, or Methods section.

n/a	Confirmed	
☐	☒	The exact sample size (*n*) for each experimental group/condition, given as a discrete number and unit of measurement
☐	☒	A statement on whether measurements were taken from distinct samples or whether the same sample was measured repeatedly
☐	☒	The statistical test(s) used AND whether they are one- or two-sided<br>*Only common tests should be described solely by name; describe more complex techniques in the Methods section.*
☒	☐	A description of all covariates tested
☒	☐	A description of any assumptions or corrections, such as tests of normality and adjustment for multiple comparisons
☐	☒	A full description of the statistical parameters including central tendency (e.g. means) or other basic estimates (e.g. regression coefficient) AND variation (e.g. standard deviation) or associated estimates of uncertainty (e.g. confidence intervals)
☐	☒	For null hypothesis testing, the test statistic (e.g. *F*, *t*, *r*) with confidence intervals, effect sizes, degrees of freedom and *P* value noted<br>*Give P values as exact values whenever suitable.*
☒	☐	For Bayesian analysis, information on the choice of priors and Markov chain Monte Carlo settings
☒	☐	For hierarchical and complex designs, identification of the appropriate level for tests and full reporting of outcomes
☒	☐	Estimates of effect sizes (e.g. Cohen's *d*, Pearson's *r*), indicating how they were calculated

Our web collection on statistics for biologists contains articles on many of the points above.

Software and code

Policy information about availability of computer code

Data collection	NMR spectroscopy was collected using Bruker IconNMR software. HRMS data was collected on Thermo Scientific Xcalibur software. Fluorescence data was collected using Shimadzu LabSolutions RF software. Cell imaging data was collected using cellSens Dimension. The absorbance was collected i-control 1.11 software. Flow cytometry was collected using BD FACScalibur. Western blot was collected using ChemiDoc Touch Imaging System. DFT calculation was collected using VASP.
Data analysis	Most of data was analyzed by OriginPro 2016 or Graph Pad Prism 9; Cell imaging data was partly analyzed using imageJ. Flow cytometry was analyzed by FlowJo v10.8.1. NMR spectroscopy was analyzed by MestReNova 12.0. Western blot was analyzed by image lab 5.2.1. DFT calculation was analyzed by vasp.6.3.0.

For manuscripts utilizing custom algorithms or software that are central to the research but not yet described in published literature, software must be made available to editors and reviewers. We strongly encourage code deposition in a community repository (e.g. GitHub). See the Nature Portfolio guidelines for submitting code & software for further information.

Data

Policy information about availability of data

All manuscripts must include a data availability statement. This statement should provide the following information, where applicable:

- Accession codes, unique identifiers, or web links for publicly available datasets
- A description of any restrictions on data availability
- For clinical datasets or third party data, please ensure that the statement adheres to our policy

The data that support the findings of this study are available from the corresponding author upon request. The data generated in this study are provided in the Supplementary Information and Source Data file.

Research involving human participants, their data, or biological material

Policy information about studies with human participants or human data. See also policy information about sex, gender (identity/presentation), and sexual orientation and race, ethnicity and racism.

Reporting on sex and gender	This work does not involves sex and gender.
Reporting on race, ethnicity, or other socially relevant groupings	This work does not address racial, ethnic, or other socially related groupings.
Population characteristics	This work does not involve population characteristics.
Recruitment	This work did not recruit any participants
Ethics oversight	This work does not involves Ethics oversight.

Note that full information on the approval of the study protocol must also be provided in the manuscript.

Field-specific reporting

Please select the one below that is the best fit for your research. If you are not sure, read the appropriate sections before making your selection.

☒ Life sciences ☐ Behavioural & social sciences ☐ Ecological, evolutionary & environmental sciences

For a reference copy of the document with all sections, see nature.com/documents/nr-reporting-summary-flat.pdf

Life sciences study design

All studies must disclose on these points even when the disclosure is negative.

Sample size	Sample sizes were indicated in the legend of each Figure and Supplementary Figure. At least 3 technical and/or biological replicates for each sample was done.
Data exclusions	No data were excluded from the analyses.
Replication	We repeated the experimental results 3 times to confirm the reproducibility, all attempts at replication were successful.
Randomization	All samples were randomly allocated into experimental groups.
Blinding	This is not relevant to this study, which does not involve clinical trials.

Reporting for specific materials, systems and methods

We require information from authors about some types of materials, experimental systems and methods used in many studies. Here, indicate whether each material, system or method listed is relevant to your study. If you are not sure if a list item applies to your research, read the appropriate section before selecting a response.

Materials & experimental systems

n/a	Involved in the study	
☐	☒	Antibodies
☐	☒	Eukaryotic cell lines
☒	☐	Palaeontology and archaeology
☒	☐	Animals and other organisms
☒	☐	Clinical data
☒	☐	Dual use research of concern
☒	☐	Plants

Methods

n/a	Involved in the study	
☒	☐	ChIP-seq
☐	☒	Flow cytometry
☒	☐	MRI-based neuroimaging

Antibodies

Antibodies used	Primary antibodies against the following proteins were used: bata-actin (4970T), Bcl-2 (3498S), and Bak (3814S), were purchased from Cell Signaling Technology. The appropriate secondary antibodies were also purchased from Cell Signaling Technology.
Validation	Beta-actin (13E5) Rabbit mAb reacts with mouse, human. It is suitable for WB, IP, IHC. Bak Rabbit antibody is suitable for WB and it reacts with mouse, human. Bcl-2 rabbit mAb is suitable for WB, IP and it reacts with human and mouse.

Eukaryotic cell lines

Policy information about cell lines and Sex and Gender in Research

Cell line source(s)	SH-SY5Y cells were purchased National Collection of Authenticated Cell Cultures.
Authentication	Cell lines were previously authenticated by cytochrome c oxidase subunit I (COI) and short tandem repeat (STR) testing.
Mycoplasma contamination	Cell lines were tested negative for mycoplasm contamination.
Commonly misidentified lines (See ICLAC register)	No commonly misidentified cell lines were used.

Plants

Seed stocks	*Report on the source of all seed stocks or other plant material used. If applicable, state the seed stock centre and catalogue number. If plant specimens were collected from the field, describe the collection location, date and sampling procedures.*
Novel plant genotypes	*Describe the methods by which all novel plant genotypes were produced. This includes those generated by transgenic approaches, gene editing, chemical/radiation-based mutagenesis and hybridization. For transgenic lines, describe the transformation method, the number of independent lines analyzed and the generation upon which experiments were performed. For gene-edited lines, describe the editor used, the endogenous sequence targeted for editing, the targeting guide RNA sequence (if applicable) and how the editor was applied.*
Authentication	*Describe any authentication procedures for each seed stock used or novel genotype generated. Describe any experiments used to assess the effect of a mutation and, where applicable, how potential secondary effects (e.g. second site T-DNA insertions, mosiacism, off-target gene editing) were examined.*

Flow Cytometry

Plots

Confirm that:

☒ The axis labels state the marker and fluorochrome used (e.g. CD4-FITC).

☒ The axis scales are clearly visible. Include numbers along axes only for bottom left plot of group (a 'group' is an analysis of identical markers).

☒ All plots are contour plots with outliers or pseudocolor plots.

☒ A numerical value for number of cells or percentage (with statistics) is provided.

Methodology

Sample preparation	SH-SY5Y cells were seeded in 6-well tissue culture plates at density of 1.5×105 cells/mL for 24 h. After treatment with STPE-PMNSs for 24 h, the cells were collected, wash and incubated with Annexin V-FITC and PI according to the instructions of the Annexin V-FITC Apoptosis Detection Kit. Then, the cells were further washed and analyzed by flow cytometry.
Instrument	BD FACScalibur

Software	FlowJo v10.8.1.
Cell population abundance	Control group: positive population: 11.22%, negative population: 88.77%; STPE-PMNSs group: positive population: 13.03%, negative population: 86.95%,; 4-AP group: positive population: 35.9%, negative population: 64.07%; 4-AP + STPE-PMNSs group: positive population: 19.01%, negative population: 80.99%.
Gating strategy	Forward and side scatter gating.

☒ Tick this box to confirm that a figure exemplifying the gating strategy is provided in the Supplementary Information.
